# Morphological, ecological and geographic differences between diploids and tetraploids of *Symphytum officinale* (Boraginaceae) justify both cytotypes as separate species

**DOI:** 10.1093/aobpla/plac028

**Published:** 2022-06-21

**Authors:** Lucie Kobrlová, Martin Duchoslav, Michal Hroneš

**Affiliations:** Plant Biosystematics & Ecology RG, Department of Botany, Faculty of Science, Palacký University, Šlechtitelů 27, 783 71 Olomouc, Czech Republic; Plant Biosystematics & Ecology RG, Department of Botany, Faculty of Science, Palacký University, Šlechtitelů 27, 783 71 Olomouc, Czech Republic; Plant Biosystematics & Ecology RG, Department of Botany, Faculty of Science, Palacký University, Šlechtitelů 27, 783 71 Olomouc, Czech Republic

**Keywords:** Autopolyploidy, Boraginaceae, cytogeography, flow cytometry, niche modelling, taxonomy

## Abstract

Polyploidization is generally considered to be an important evolutionary driver affecting the genetic diversity, that can alter the morphology, phenology, physiology or ecology of plants, which in turn may make the taxonomy of polyploids more difficult. One such example is the *Symphytum officinale* complex, a polyploid species group represented by three major cytotypes: tetraploids (2*n* = 48), less common, geographically restricted diploids (2*n* = 24) and hypotetraploids (2*n* = 40). In most European floras only one polymorphic species, *S. officinale*, is widely recognized, while the particular cytotypes are usually considered conspecific. Our study provided a thorough evaluation of the ploidy level diversity, morphological and ecological variation, with a special attempt to clarify the status of ‘white-flowered’ diploids. Using flow cytometry, we identified three cytotypes: widespread tetraploids (76.1 %); less frequent diploids (23.6 %) with scattered distribution across the range of tetraploids and confined only to several areas of Europe; and extremely rare triploids (0.3 %). Diploids and tetraploids showed diffuse parapatric pattern of distribution, with only four mixed-cytotype populations (2.7 %) found, but almost entirely without triploids, suggesting reproductive isolation between di- and tetraploids. Niche of diploids falls nearly completely within the niche of tetraploids that showed niche expansion. Tetraploids also showed a shift in niche optimum towards a less continental and colder climate, coupled with expansion to more disturbance-prone sites with higher nutrient availability. Diploids were clearly distinguishable morphologically from tetraploids. The morphological differentiation of studied cytotypes appears to be taxonomically significant, especially in combination with ecological differences and the apparent presence of hybridization barriers. Both cytotypes should be treated as separate species (i.e. *S. bohemicum* and *S. officinale* s. str.).

## Teaser

Polyploid complexes are of major interest for biologists as they provide insight into microevolutionary processes in plant populations. One such polyploid complex is common comfrey (*Symphytum officinale*). Only one polymorphic species, *S. officinale*, is widely recognized in European floras. Our study provided an insight into its cytotype diversity and connected morphological and ecological variation. We identified four cytotypes: widespread tetraploids, less frequent diploids, rare hypotetraploids and extremely rare triploids. The morphological differentiation and ecological segregation of di- and tetraploids and the apparent presence of hybridization barriers justify recognition of both major cytotypes as separate species (i.e. diploid *S. bohemicum*and tetraploid *S. officinale*).

## Introduction

Polyploidization is generally considered as a major evolutionary force in higher plants (Otto and Whitton 2000). Chromosome doubling acts as an immediate strong reproductive barrier and affects many important processes and traits at different levels of organization from genome to individual plant (Levin 2002). After formation, polyploids often diverge from their diploid progenitors in morphology, physiology and ecology, which may affect their distribution pattern, resulting in shifts in range between diploid and polyploid relatives (Ramsey and Schemske 2002; te Beest *et al.* 2012; van de Peer *et al.* 2017). However, frequently reported wider ranges or more extreme niches of polyploids are not a general trend in plants, and in many mixed-ploidy complexes, even the opposite relationship is known to occur (Husband *et al.* 2013; Visger *et al.* 2016; Spoelhof *et al.* 2017). One likely reason for the various distribution patterns of the cytotypes might be the different routes leading to polyploidization. Two main paths are usually considered, autopolyploidization (polyploidization on intraspecific level) and allopolyploidization (polyploidization coupled with interspecific hybridization). Autopolyploidy, in contrast to allopolyploidy, does not inevitably produce transgressive traits to boost adaptive ecological divergence (Parisod *et al.* 2010) and autopolyploids might escape from minority cytotype disadvantage (Levin 1975) and achieve establishment alternatively also by spatial separation unaccompanied by niche divergence, e.g. by a chance colonization of recently opened (disturbed) habitat (te Beest *et al.* 2012; Godsoe *et al.* 2013). Moreover, there is still different perception of allo- and autopolyploids in taxonomy, as allopolyploids are usually considered as different taxa given their divergent morphology from the diploid ancestors while autopolyploids are regarded as conspecific with diploids due to their high morphological similarity (Soltis *et al.* 2007). In their review, Soltis *et al.* (2007) argued for taxonomic recognition of autopolyploids after a careful examination of the studied complex. However, such studies are still relatively sparse.

In this study, we focus on the *Symphytum officinale* complex, which includes the most widespread *Symphytum* (Boraginaceae, Boragineae; Chacón *et al.* 2016) representatives in Europe. An extensive cytological variation has been observed in this complex which corresponds to three main cytotypes: diploid (2*n* = 24), tetraploid (2*n* = 48) and dysploid (hypotetraploid, 2*n* = 40). Tetraploids are presumably of autopolyploid origin (Gadella and Kliphuis 1972) and represent the most frequently documented ploidy level covering the whole range of the complex, whereas the reports of diploids are rather solitary and scattered across Europe (Basler 1972; Gadella and Kliphuis 1972; Wille 1998; Peruzzi *et al.* 2001). Zones with occasional sympatric growth of diploids and tetraploids have been observed in some parts of Europe (Gadella and Kliphuis 1967; Wille 1998), but there are almost no records of triploids (2*n* = 36; Basler 1972). The hypotetraploids are the rarest of the three main ploidy levels and they also have very scattered distribution (Gadella and Kliphuis 1967; Májovský and Uhríková 1985; Peruzzi *et al.* 2001).

**Figure 1.**
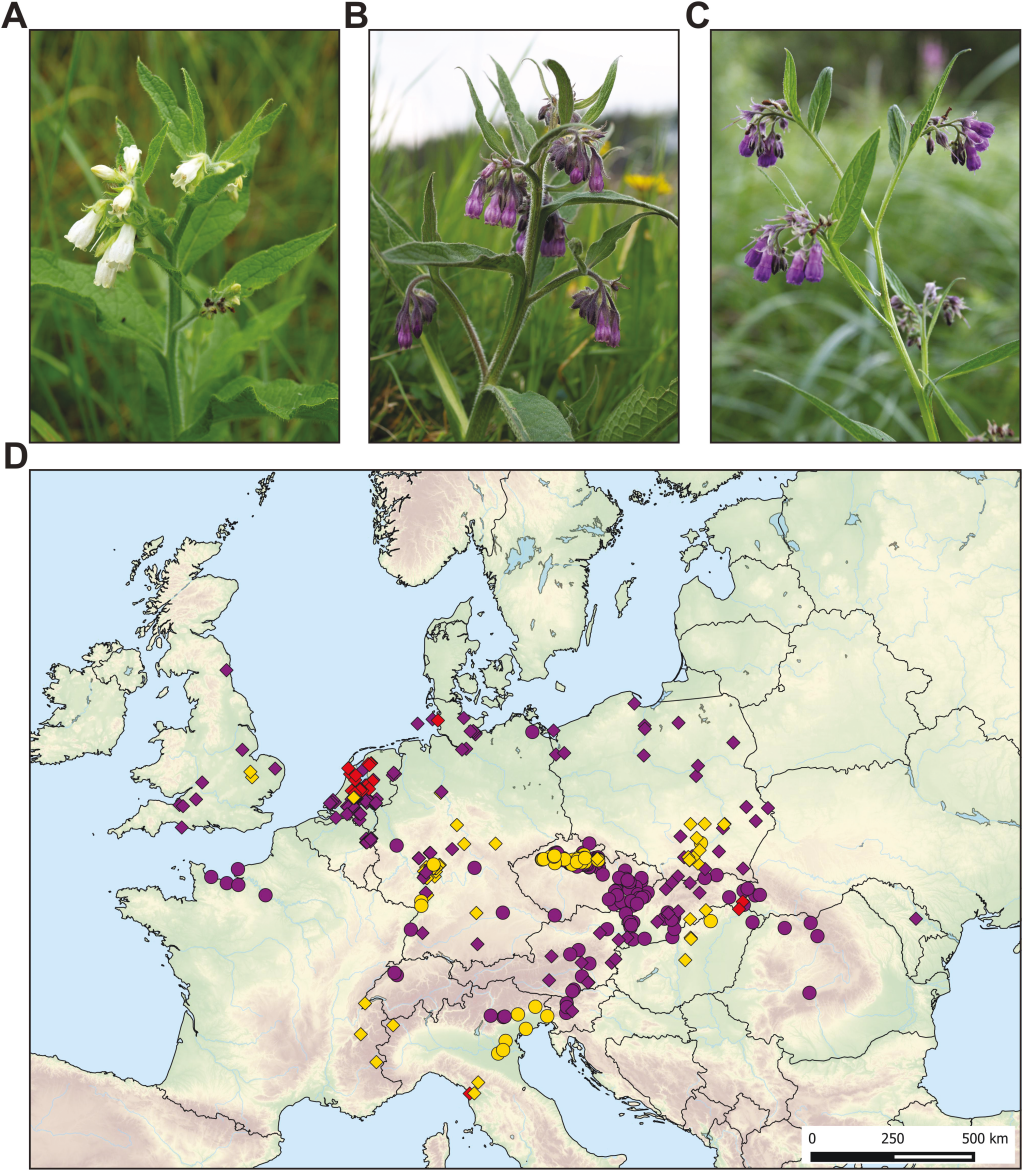
The members of the *Symphytum officinale* complex. (A) *Symphytum bohemicum* (2*x*), (B) *S. officinale* (4*x*), (C) *S. tanaicense* (4*x*−). (D) Distribution of cytotypes of *S. officinale* complex in Europe. Diamonds represent chromosome number reports, whereas flow cytometric data are marked with dots. Yellow—diploids; violet—tetraploids; red—hypotetraploids. Authors of photographs: L. Kobrlová (A), M. Duchoslav (B), D. Dítě (C).

The complex is known for its high morphological variability that led to confusion and non-uniformity of taxonomic concepts across European floras (Table 1). Flower colour varying from pure white to dark purple, corolla shape and size, and decurrency of leaves to stem are considered the most important characters for taxonomy of this group (Smejkal 1978; Májovský and Hegedüšová 1993; Peruzzi *et al.* 2001). However, it is not always clear how the morphology is connected with a particular ploidy level. In the Czech Republic and Slovakia, the diploids are linked to the name *S. bohemicum* (Fig. 1A) and tetraploids to *S. officinale* s. str. (Fig. 1B). Elsewhere in Europe, diploids and tetraploids are mainly considered as mere cytotypes of *S. officinale*, while hypotetraploids are almost exclusively called *S. tanaicense* (Table 1; Fig. 1C). Dysploidy contrary to autopolyploidy is generally considered a strong reproductive barrier (Mandáková and Lysák 2018); therefore, based on chromosome number and morphological differences, *S. tanaicense* is generally considered as a separate species (Table 1).

**Table 1.** Historical overview of taxonomic treatments in *Symphytum officinale* complex.

Kuznetsov (1910) Pawłowski (1961) Gadella and Kliphuis (1967, 1972) Perring (1975) Gadella *et al.* (1983) Fischer *et al.* (2008) Jäger (2009) Király *et al.* (2011) Gracia and Castroviejo (2012)	Bucknall (1913) Gadella (1972) Sandbrink *et al.* (1990) Peruzzi *et al.* (2001) Cecchi and Selvi (2015, 2017)	Popov (1953)	Pawłowski (1963)	Pawłowski (1972)	Smejkal (1978) Májovský and Hegedüšová (1993) Wille (1998) Fedorov (2001) Czerepanov (2007)	Schmeil and Fitschen (1988) Martinčič (2007)	Slavík (2000) Danihelka *et al.* (2012)	Stace (2010)
*S. officinale*	*S. officinale*	*S. officinale* var. *purpureum*	*S. officinale* subsp. *officinale* var. *officinale*	*S. officinale* subsp. *officinale*	*S. officinale*	*S. officinale* subsp. *officinale*	*S. officinale*	*S. officinale* subsp. *officinale*
—	—	*S. officinale* var. *ochroleucum*	*S. officinale* subsp. *officinale* var. *bohemicum*	—	*S. bohemicum*	*S. officinale* subsp. *bohemicum*	*S. bohemicum*	*S. officinale* subsp. *bohemicum*
—	*S. tanaicense*/*S. uliginosum*	*S. officinale* var. *lanceolatum*	*S. officinale* subsp. *uliginosum*	*S. officinale* subsp. *uliginosum*	*S. tanaicense*	*S. officinale* subsp. *uliginosum*	—	—

Here, our objective was to explore the cytotype diversity of the *S. officinale* complex in Europe and its implications for taxonomy with respect to diploids and tetraploids. We summarized the geographic distribution of the cytotypes and asked whether the observed spatial patterns might be explained by a combination of abiotic factors. In addition, we investigated whether morphology correlates with the established ploidy levels and therefore can be used for unequivocal determination of taxa. For that, we (i) revised published chromosomal counts, (ii) investigated the diversity and distribution of cytotypes throughout Europe using flow cytometry, (iii) examined the morphological differences between cytotypes using multivariate morphometrics and (iv) studied the ecological differences between cytotypes on the continental scale using niche analyses and on local spatial scales in the area of sympatry using records of vegetation surrounding occurrence points. More specifically, we placed particular emphasis on the white-flowered plants and their relation to the name *S. bohemicum*, in order to deal with the taxonomic chaos that is connected with this taxon.

## Materials and Methods

### Study species

The members of the *S. officinale* complex are traditionally placed in the sect. *Symphytum* characterized by fusiform, ± vertical rhizomes, decurrent leaves, broadly triangular lanceolate, acute, densely papillate faucal scales, stamens with connectives projecting beyond thecae and smooth, shiny nutlets (Pawłowski 1961). The complex consists of widespread *S. officinale* (Fig. 1B) and several local taxa sometimes recognized in regional floras (Table 1), i.e. *S. tanaicense* (Fig. 1C) from the Don river delta in the south-western Russia, *S. uliginosum* from Hungary and *S. bohemicum* (Fig. 1A) from the Elbe basin in the Czech Republic. The conspecificity of *S. tanaicense* and *S. uliginosum* has already been discussed by Degen (1930) who identified the name *S. tanaicense* as the oldest validly described name. *Symphytum officinale* s. str. represents the widest ranging member of the whole genus, growing in most of Europe to Western Siberia and Central Asia (Meusel *et al.* 1978; Hultén and Fries 1986). It is also cultivated worldwide as a nectar source, fodder plant or green manure, sometimes escapes from cultivation and becomes naturalized, e.g. in North and South America, China and New Zealand (Gadella 1984; Hultén and Fries 1986; Zhu *et al.* 1995; Jørgensen *et al.* 2014). The possible interploidy hybridization between diploids and tetraploids, based on the intermediate morphology of plants, is rarely documented (Smejkal 1978; Májovský and Hegedüšová 1993; Buch *et al.* 2007), and such plants have been described as *S. × rakosiense*. However, chromosomes of any of these plants have never been counted.

### Plant material

Samples were collected between 2014 and 2021 in Europe, with special attention to Central Europe. In total, 156 populations and 776 individuals were sampled **[see****Supporting Information—Table S1****]**. Hypotetraploids (i.e. *S. tanaicense*) have not been subject of a detailed study. The DNA-ploidy level was determined for all sampled individuals by flow cytometry. The number of individuals sampled per population varied from 1 to 15 (mean ± SD: 5 ± 2). Voucher specimens are deposited in the Herbarium of Palacký University in Olomouc (OL).

### Flow cytometry and chromosome number review

DNA-ploidy level (Suda *et al.* 2006) and absolute genome size (AGS; Greilhuber *et al.* 2005) were estimated using flow cytometry. Generally, fresh leaf tissue has been used (for both the relative genome size, RGS and AGS), but in some cases silica-dried material has also been analysed (only for RGS). Samples were prepared according to the protocol described by Kobrlová *et al.* (2016) and were carried out on the following flow cytometers using two different fluorochromes staining: (i) BD Accuri C6 (BD Biosciences, San Jose, CA, USA)—propidium iodide (PI); (ii) Partec PAS (Partec GmbH, Münster, Germany)—PI; (iii) Partec Cy Flow ML (Partec GmbH)—4,6-diamidino-2-phenylindole (DAPI). *Pisum sativum* ‘Ctirad’ (2C = 9.09 pg; Doležel *et al.* 1998) and *Zea mays* ‘CE-777’ (2C = 5.92 pg, the value recalculated to the primary standard *P. sativum*) were used as internal references. The ploidy level of each sample was determined by the position of its G0/G1 peak relative to the G0/G1 peak of an internal standard. Generally, for measurements using both PI and DAPI, histograms with coefficients of variation (CV) for the G0/G1 peaks of the analysed sample and the standard less than 5 % were accepted. For each sample, the fluorescence intensity of 3000 and 5000 particles was recorded for DNA-ploidy level (RGS) and for AGS (expressed as 2C value) estimations, respectively. For AGS estimation, each sample was prepared and analysed three times. The general rule that the variation between the highest and the lowest obtained AGS value for one sample does not exceed 2 % has been followed (see Doležel *et al.* 2007). The ploidy level was calibrated using population ID 38 from which previous chromosome record exists (Murín and Májovský 1982).

In addition, a complete bibliographic review of published chromosome counts was performed **[see****Supporting Information—Table S2****]** to find out the karyological variability of the complex. Together with flow cytometric data, the compiled chromosome counts were used to build a distribution map of the *S. officinale* complex. Only data with given localities were used and georeferenced.

### Estimation of niches in environmental space at large spatial scale and distribution modelling analyses

Climatic and soil data related to different ecophysiological constraints of plant species were downloaded from various open-source databases. The WorldClim 2.1 database (Fick and Hijmans 2017) was used for the extraction of annual trends and extreme limiting conditions related to precipitation, temperature and solar radiation (bio1–19 variables; mean annual solar radiation [kW·m^−2^]). Quantitative physical and chemical soil variables were downloaded from the SoilGrid database (Hengl *et al.* 2017). All downloaded variables had a resolution of 30 arcseconds (~1 km).

All data handling and calculations were conducted in R 4.4.4 (R Development Core Team 2021). To trim the predictor set to reduce collinearity, all downloaded environmental variables were examined for pairwise correlations in ENMTools 1.0 (Warren *et al.* 2021), using data from the entire study area (5°W–35°E, 40°N–60°N). After evaluation, 14 variables not highly correlated (|*r*| ≤ 0.75) were retained (Table 2) and used in further analyses.

**Table 2.** Mean relative contribution (%) of each environmental variable for mean MaxEnt model predicting the probability of di- (2*x*) and tetraploid (4*x*) occurrences in studied range. The percentages are based on a heuristic method that estimates the proportional contribution of each variable to the model training gain for every iteration during model fitting. Values of three variables with highest average relative contribution to the model training for each cytotype are in bold.

Variable	Relative contribution (%)	
	2*x*	4*x*
Mean annual solar radiation (SRAD)	**25.9**	**32.6**
Annual mean temperature (bio1)	**17.6**	**15.3**
Max temperature of warmest month (bio5)	**11.3**	0.4
Mean temperature of wettest quarter (bio8)	8.6	2.9
Weight percentage of the clay particles in soil (<0.0002 mm) (clyppt)	7.9	8.0
Temperature annual range (bio7)	6.4	12.2
Precipitation seasonality (bio15)	5.2	**12.3**
Min temperature of coldest month (bio6)	5.1	2.6
Mean diurnal range (bio2)	4.0	7.6
Cation exchange capacity of soil (cecsol)	2.5	1.9
Available soil water capacity (volumetric fraction) until wilting point (WWP)	2.3	0.7
Precipitation of driest quarter (bio17)	2.3	1.0
Volumetric percentage of coarse fragments (>2 mm) (crfvol)	0.7	1.4
pH index measured in water solution (pH)	0.3	1.2

Georeferenced location data showed highly unequal sampling. After preliminary analyses, different thinning settings were selected for each cytotype. To remove aggregation and to obtain reasonable number of both di- and tetraploid occurrences, occurrences closer than a distance of 5/15 km (diploids/tetraploids) from each other were removed, separately for each cytotype, in Humboldt 1.0 (Brown and Carnaval 2019). This resulted in 224 localities (2*x*, *n* = 77, 4*x*, *n* = 147), which were used for subsequent analyses.

The environmental niche space occupied by diploids and tetraploids was constructed using environmental principal component analysis (PCAenv; Broennimann *et al.* 2012). Niche overlap was estimated by Schoener’s *D* calculated directly from environmental niche space (Warren *et al.* 2008). The background area was taken from 200-km buffer zones around thinned occurrences. The number of background points equalled 10 000 per cytotype. Niche equivalency and similarity between diploids and tetraploids were tested by niche equivalency and similarity tests (Broennimann *et al.* 2012).

To compare the niches in terms of optima and breadths, 100 random pixels, weighted by density along PC1 and PC2, were sampled in the niche of each cytotype and their scores were extracted (Broennimann *et al.* 2012). The niche optimum and the niche breadth were calculated as the median and the variance of the sampled scores along the PCA axes. This procedure was repeated 100 times. The distributions of values of niche optimum and breadth for each PCA axis were compared between cytotypes using randomization test using *t*-statistics and 999 permutations. Niche change of tetraploids relative to diploids was estimated using the indices of niche change (Petitpierre *et al.* 2012; Guisan *et al.* 2014): niche expansion (*E*), i.e. proportion of the niche space of the tetraploids not overlapping the niche of the diploids; niche unfilling (*U*), i.e. proportion of the niche of the diploids not overlapping the niche of the tetraploids; and niche stability (*S*_*n*_, *S*_*e*_), i.e. proportion of the niche of either diploids (*S*_*n*_) or tetraploids (*S*_*e*_), shared with the other cytotype. All environmental niche analyses were performed using ecospat 3.2 (Di Cola *et al.* 2017).

Cytotype distribution modelling analyses were performed with maximum entropy modelling (MaxEnt) using MAxEnt 3.4.4 (Phillips *et al.* 2006, 2008). Spatial predictive models were created based on the same subset of environmental variables and occurrence data as PCAenv, plus 10 000 pseudo-absences sampled randomly within the predefined study area based on known distribution of *S. officinale* complex, separately for each cytotype. To reduce uncertainty and to produce robust models, we used 10 replicate runs with cross-validation. The presence localities of each cytotype were divided randomly into training (80 %) and test (20 %) subsets. We used the default settings of the program. Models were evaluated based on the area under curve of the receiver operator characteristic (AUC of ROC), and combined final model is presented for each cytotype. Relative contribution of each environmental variable to the MaxEnt model was determined for each run and averaged over replicated runs (Table 2). Response curves of selected environmental variables with high average percent contribution to the models for both or one of cytotypes were reported. To visualize the relative suitability within studied range, final models with the log-log (clog-log) format were used as model output for each cytotype. Subsequently, the final models were converted to a binary format using the threshold rule ‘maximum training sensitivity and specificity’ (Liu *et al.* 2016) and visualized.

### Ecological differences between cytotypes on the local spatial scale

To test ecological differentiation of cytotypes on local spatial scales, the Elbe basin area (Central Bohemia, Czech Republic) has been selected. We acquired 3809 phytosociological relevés from the Czech National Phytosociological Database (Chytrý and Rafajová 2003) with the presence of either *S. bohemicum* or *S. officinale*, which correspond to diploids and tetraploids, as indicated by our results. Subsequently, only the relevés recorded in the localities with the confirmed occurrence of *S. bohemicum* (see Kaplan *et al.* 2016) were selected, resulting in 54 relevés. When more than two relevés from the same locality were available, only two relevés were randomly selected. Relevés with the occurrence of *S. officinale* were then selected from the data set based on their position within an approximately 20 km radius from the nearest *S. bohemicum* relevé (considered as sympatric occurrences to *S. bohemicum* relevés), resulting in additional 78 relevés.

The ecological differences of both cytotypes were established using Ellenberg-type indicator values (EIVs) derived for the Czech flora (Chytrý *et al.* 2018). Ellenberg-type indicator values for nutrients, light, temperature, moisture, soil reaction and salinity were calculated for each relevé in Juice 7.1 (Tichý 2002), excluding EIVs for both *Symphytum* taxa from the calculation. Differences in cover-unweighted average EIVs between relevés with the presence of either *S. bohemicum* or *S. officinale* were analysed using one-way ANOVA with the modified permutation test with 499 permutations using MoPeT 1.2 (Zelený and Schaffers 2012).

### Morphometric analyses

In total, 151 plants (40 diploids, 111 tetraploids) from 18 populations (5 diploid, 13 tetraploid) were morphologically investigated **[see****Supporting Information—Table S1****]**. Only well-developed plants with at least five flowers were collected. For each plant, 37 quantitative and four qualitative (Table 3) morphological characters were measured *in situ* using a digital calliper or retractable meter. Nine additional ratios were calculated and several measured characters were thus excluded from the analyses (Table 3).

**Table 3.** Morphological differentiation of diploids (2*x*) and tetraploids (4*x*) of *S. officinale* complex. *t*—results of *t*-tests. χ^2^—results of χ^2^ tests. *P*-values in bold indicate significant difference after Bonferroni correction. Numbers are rounded to mm, minor inaccuracies in the totals may occur.

Quantitative morphological character		Abbreviation	2*x*			4*x*			*t*	*P*
			Mean (mm)	± SD	Min–max (mm)	Mean (mm)	± SD	Min–max (mm)		
Height of stem		ST_h	734	272	389–1512	692	176	401–1152	1.092	0.276
Width of stem		ST_w	8	3	4–16	9	2	6–13	−4.272	**<0.001**
Width of stem cavity		CAV_w	2	2	0–7	3	1	0–6	−0.500	0.618
Width of stem wall		STW_w	5	1	2–9	6	1	3–10	−5.364	**<0.001**
Stem/cavity width ratio		r_ST_CAV	4	3	0–15	3	2	0–11	0.812	0.418
Number of branches		no_BR	4	2	0–11	5	2	1–10	−4.254	**<0.001**
Length of rosette leaf		R_LF_l	363	96	168–644	401	128	160–784	−1.715	0.088
Length/width ratio of rosette lamina^B^		R_LAM_r	3	1	2–5	2	0	1–4	2.782	0.006
Width of lower petiole wing		PET_WING_w	3	1	1–5	4	2	2–19	−2.873	0.005
Length of lower leaf		L_LF_l	325	124	119–687	302	93	146–686	1.243	0.216
Length/width ratio of lower leaf lamina^B^		L_LAM_r	3	1	2–5	3	0	2–4	−0.043	0.966
Internode length/wing width below lower leaf ratio^B^		r_LINT_WING	2	1	0–7	2	2	0–15	0.265	0.791
Width of wing below lower leaf		WING_L_w	1	1	0–2	2	1	0–5	−4.241	**<0.001**
Length of middle leaf		M_LF_l	227	65	96–402	225	56	118–386	0.133	0.894
Length/width ratio of middle leaf lamina^B^		M_LAM_r	3	1	2–5	3	1	2–5	−2.314	0.022
Internode length/wing width below middle leaf ratio^B^		r_MINT_WING	1	0	0–2	1	1	0–3	1.512	0.133
Width of wing below middle leaf		WING_M_w	1	1	0–3	3	2	0–8	−6.868	**<0.001**
Length of upper leaf		U_LF_l	147	44	75–255	138	32	62–217	1.238	0.218
Length/width ratio of upper leaf lamina^B^		U_LAM_r	3	1	2–7	4	1	2–5	−2.972	0.003
Internode length/wing width below upper leaf ratio^B^		r_UINT_WING	1	0	0–2	1	0	0–4	3.222	0.002
Width of wing below upper leaf		WING_U_w	2	1	0–3	3	1	0–8	−5.531	**<0.001**
Number of flowers		no_FLW	51	24	22–108	58	16	32–96	−2.145	0.034
Length of peduncle		PED_l	5	1	3–7	8	1	4–13	−11.253	**<0.001**
Length of calyx		CAL_l	7	1	6–9	10	1	7–13	−12.454	**<0.001**
Length of calyx lobe^A^		CALL_l	5	1	4–7	7	1	5–9	−10.583	**<0.001**
Width of calyx lobe		CALL_w	2	0	2–3	3	0	2–4	−6.565	**<0.001**
Length of corolla		COR_l	13	1	10–16	15	1	13–18	−9.417	**<0.001**
Length of lowered corolla part^A^		CORT_l	7	1	5–9	9	1	7–10	−11.073	**<0.001**
Width of corolla		COR_w	6	1	5–8	7	1	5–9	−8.919	**<0.001**
Width of lowered corolla part		CORT_w	4	0	3–5	5	1	3–6	−10.205	**<0.001**
Length of style		STY_l	15	1	12–18	16	1	13–9	−7.164	**<0.001**
Corolla/style length ratio		r_COR_STY	1	0	1–1	1	0	1–1	−2.657	0.009

Qualitative morphological character			Proportion (%)			Proportion (%)			χ^2^	*P*
Colour of plant	Light green	PL_col	87.5			28.0			42.411	**<0.001**
	Dark green		12.5			72.0				
Branching^A^	Unbranched	BR	2.5			3.6			36.662	**<0.001**
	Upper stem		50.0			10.8				
	Middle stem		32.5			21.6				
	Lower stem		15.0			64.0				
Flower colour	Yellowish white	FLW_col	100			0.0			151.000	**<0.001**
	Pure white		0.0			23.0				
	Other		0.0			77.0				
Shape of style	Straight	STY_s	37.5			60.4			17.874	**<0.001**
	Intermediate		12.5			23.4				
	Hooked		50.0			16.2				

Excluded from multivariate analyses.

Calculated from length and width of rosette, lower, middle and upper leaves, and length of internode and respective petiole wing below lower, middle and upper leaf. These characters were excluded from all analyses and left only as ratios.

Descriptive statistics were calculated for each quantitative character and each cytotype. Intercytotype differences in quantitative traits were tested using *t*-test and proportional differences in qualitative traits were tested using χ^2^ tests. Bonferroni correction was applied to adjust the *P-*values of these tests.

The correlations of quantitative characters of the initial data matrix were tested using the Pearson’s correlation coefficient. One character of pair of highly correlated characters (|*r*| ≥ 0.85) was excluded from further analyses (Table 3). Principal component analysis was run to provide insight into the overall pattern of morphological variation and to observe the grouping of individuals in the ordination space. We performed PCA both with and without qualitative data and the results were almost identical (not shown); therefore, only PCA with qualitative data included is shown here. Canonical discriminant analysis (CDA; Legendre and Legendre 1998) was performed to determine the extent of morphological separation between cytotypes. A step-wise forward selection of characters with 999 permutations was used to find a set of most important characters used for discrimination. A multistate qualitative character flower colour was excluded from the data set prior to CDA performance. Principal component analysis and CDA were performed in Morphotools 1.1 (Koutecký 2015) in R 4.4.4. All analyses used individuals as operational taxonomic units (OTUs).

## Results

### Ploidy variation, genome size and cytogeography

Bibliographic review of 298 chromosome counts **[see****Supporting Information—Table S2****]** from the *S. officinale* complex confirmed the occurrence of three major cytotypes in Europe: (i) diploids (2*n* = 2*x* = 24 + 0–4 B); (ii) tetraploids (2*n* = 4*x* = 48) and (iii) hypotetraploids (2*n* = 4*x*− = 40; Fig. 1D) and additional 12 rare cytotypes. The most common and widespread cytotype is tetraploid (154 records, 51.7 %) that occurred across Europe. On the contrary, diploids (76 records, 25.5 %) have a very scattered distribution through Europe and have been reported from Great Britain, France, The Netherlands, Italy, Germany, Czech Republic, Poland, Slovakia and Hungary. Hypotetraploid cytotype (25 records, 8.4 %) has been detected in the Netherlands, Germany, Slovakia and Italy. Furthermore, several aneuploid chromosome counts have also been documented (29 records, 9.7 %: 1 record from diploid and 28 records from tetraploid populations, respectively), and additional 14 chromosome reports (4.7 %) were assessed as unclear and mostly belonging to other taxa than *S. officinale* complex **[see****Supporting Information—Table S2****]**.

**Figure 2.**
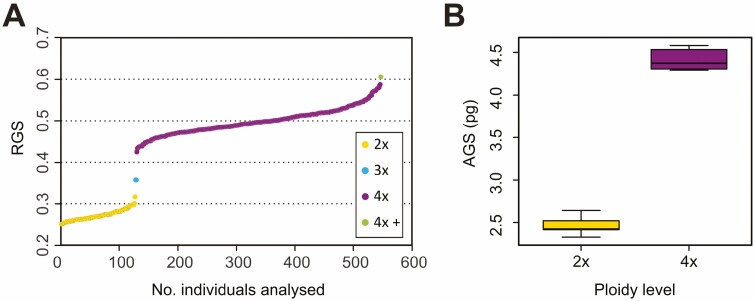
Variation of the nuclear DNA amount of diploids and tetraploids of the *Symphytum officinale* complex. (A) Variation in RGS (the relative genome size), sorted according to increasing relative 2C values **[see****Supporting Information—Table S2****]**, and (B) difference in AGS (the absolute genome size, 2C values) of the respective cytotypes (2*x*, 4*x*).

Three DNA-ploidy levels were detected by flow cytometry: diploids (183 plants/35 populations), triploids (2 plants/1 population) and tetraploids (591 plants/118 populations; **see****Supporting Information—Table S3**). Tetraploids have been confirmed in France, Switzerland, Italy, Slovenia, Germany, Austria, Czech Republic, Slovakia, Hungary, Romania and Ukraine, while diploids have been found in Germany, Czech Republic, Hungary and Italy (Fig. 1D; **see****Supporting Information—Table S1**). We were unable to confirm the presence of hypotetraploids due to the lack of *S. tanaicense* populations in our sampling as we did not focus the sampling on them. The RGS of all ploidy levels formed non-overlapping groups (Fig. 2A) which allowed all individuals to be unambiguously assigned to their ploidy level. Furthermore, in one tetraploid population (ID 68) an individual with ca. 30 % RGS higher than regular was recorded (see 4*x*+ in Fig. 2A; **see****Supporting Information—Table S1**; note that a pentaploid ploidy level cannot be excluded). Considering populations with at least two individuals analysed (149 populations, 95.5 %), most of them comprised a single cytotype and only four mixed-ploidy populations (ID 9, 76, 130—2*x* + 4*x*; ID 133—2*x* + 3*x*) were discovered **[see****Supporting Information—Table S1****]**.

The mean AGS was 2.46 ± 0.10 pg in diploids and 4.41 ± 0.13 pg in tetraploids, with the mean monoploid genome size (1C*x* value) 1.23 pg and 1.10 pg, respectively (Fig. 2B; **see****Supporting Information—Table S4**).

### Environmental niches at large spatial scale and cytotype distribution modelling analyses

**Figure 3.**
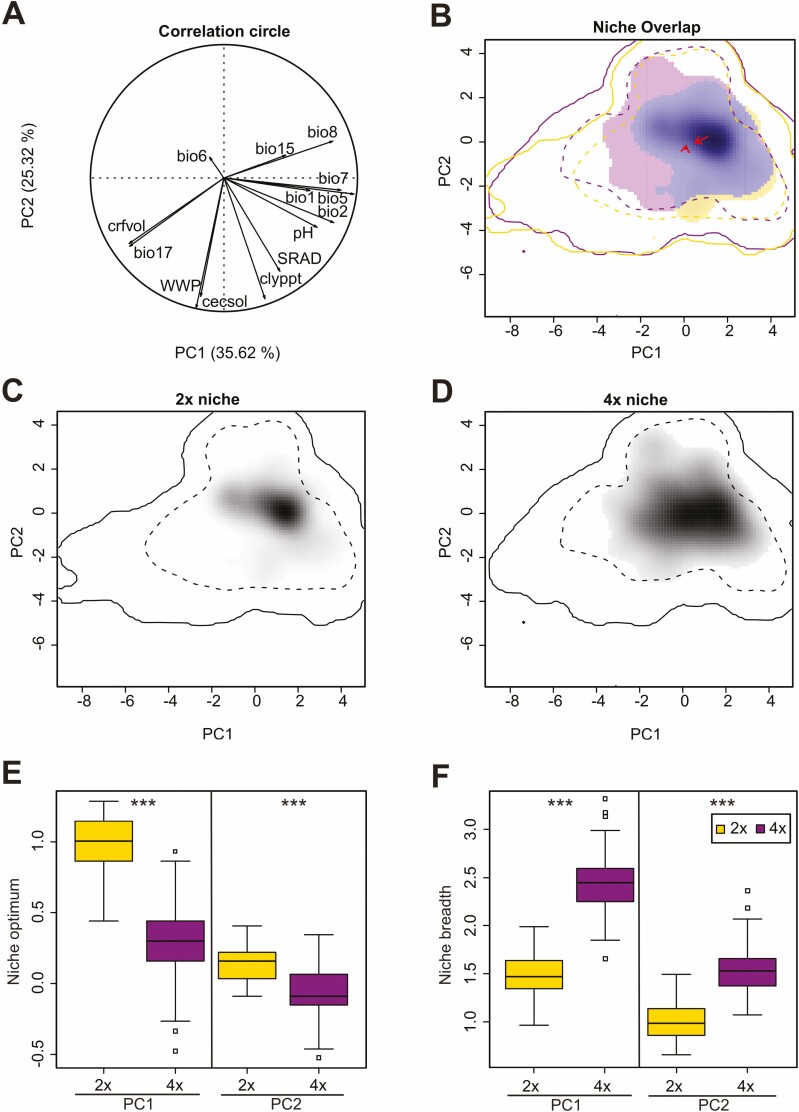
Niches of diploids (2*x*) and tetraploids (4*x*) of *Symphytum officinale* complex in the environmental space along the first two axes of PCA (PCAenv). (A) The correlation circle shows the loadings of the individual environmental variables to the first two PCA axes. bio1 (mean annual temperature), bio2 (mean diurnal temperature range), bio5 (maximal temperature of warmest month), bio6 (minimal temperature of coldest month), bio7 (temperature annual range), bio8 (mean temperature of wettest quarter), bio15 (precipitation seasonality), bio17 (precipitation of driest quarter), cecsol (cation exchange capacity of soil, mmol(c) kg^−1^), clyppt (weight percentage of clay particles < 0.0002 mm), crfvol (volumetric percentage of coarse fragments > 2 mm), pH (soil acidity measured in KCl solution), SRAD (mean annual solar radiation, kW·m^−2^), WWP (available soil water capacity until wilting point, %). (B) The niche overlap between diploids and tetraploids and (C, D) niches of the respective cytotypes (2*x*, 4*x*). Niche overlap is shown in blue, and parts of niche of the one cytotype unfilled by that of the second are in yellow (2*x*) and purple (4*x*). Shading shows the density of the occurrences of the cytotype. Full and dashed contour lines illustrate 100 % and 50 %, respectively, of available (background) environments delimited by a 200-km buffer zone around the occurrence points of each cytotype. (E) Box plots of niche optima and (F) niche breadths of cytotypes along the first two PCA axes (PC1, PC2). Results of randomization tests comparing niche optima and niche breadth between cytotypes for each PCA axis are presented above box plots (***P < 0.001).

The first two PCAenv axes explained 35.6 % and 25.3 % of the total variation in the environmental space available within the studied ranges of cytotypes (Fig. 3A). The PC1 axis mirrored increasing mean (bio1, bio8) and maximal temperatures (bio5) and increasing seasonality in temperature (bio2, bio7) and precipitation (bio15) (Fig. 3A). Additionally, soil pH increased along PC1. The PC2 axis mirrored the gradient of soil physical variables, from more clayey soils (clyppt) with higher cation exchange capacity (cecsol) and higher available soil water capacity (WWP) to more sandy soils with lower WWP and cecsol. Both cytotypes avoided the coldest climatic conditions with less seasonality in temperature and precipitation of the available environmental space (Fig. 3C and D).

The niche overlap (Schoener’s *D*) between the cytotypes was 0.668, suggesting a moderate to high niche overlap. Niche equivalency test suggested marginally non-significant differences between the niches of di- and tetraploids (*P* = 0.054). Niche similarity tests suggested that the niches of cytotypes were significantly more similar than expected by chance, regardless of the direction of the test (all *P* < 0.006). The niche of diploids falls nearly completely within the niche of tetraploids (Fig. 3B), i.e. tetraploids have greater niche breadth than diploids (Fig. 3F). Consequently, tetraploids showed niche expansion (*E* = 0.182) and an almost complete filling of the diploid niche (*S*_*e*_ = 0.818, *U* = 0.026). Tetraploids also showed a shift in niche optimum (Fig. 3E) towards a less continental and colder climate, occasionally coupled with expansion to more sandy soils with lower cation exchange capacity.

**Figure 4.**
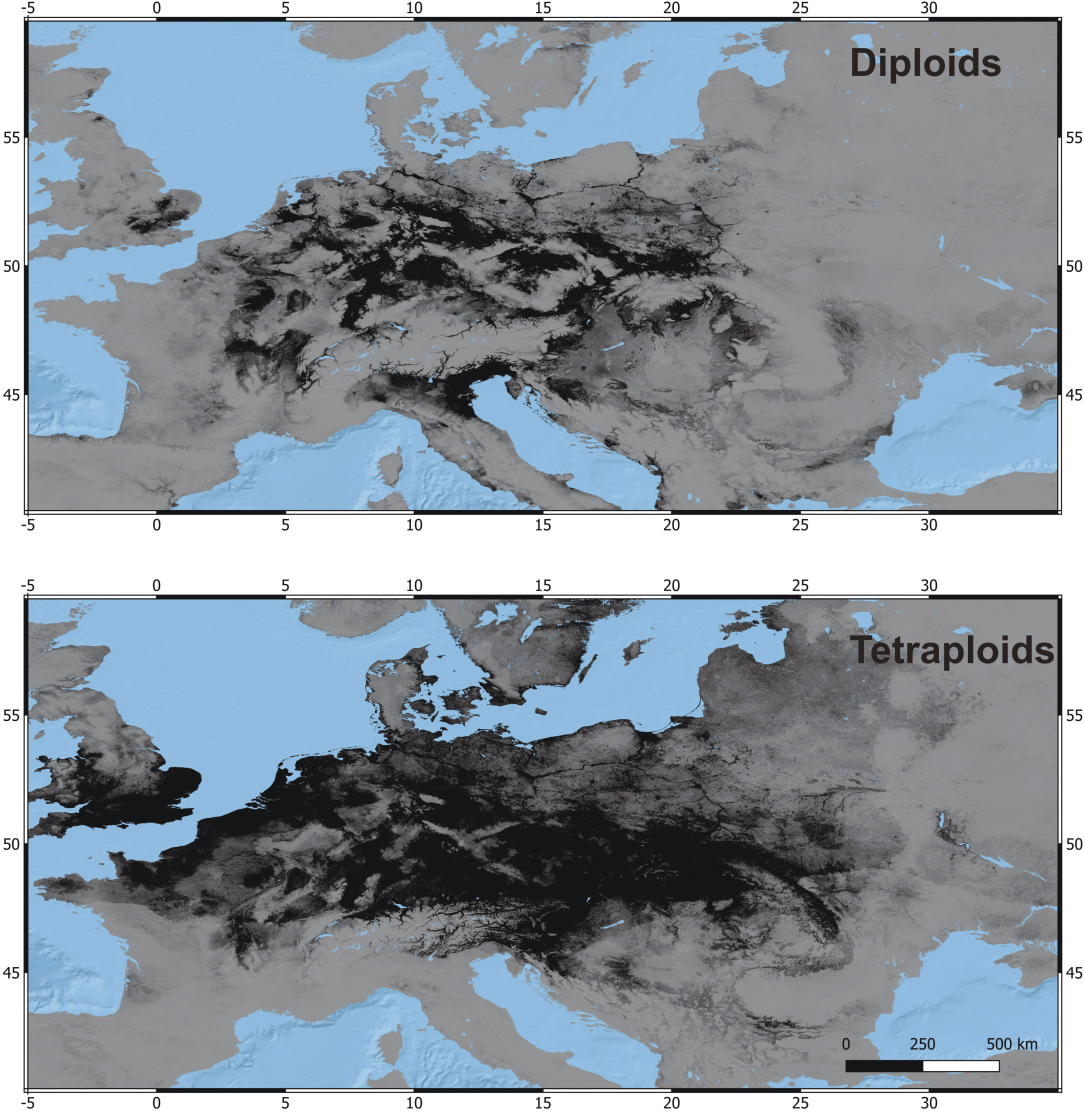
Predicted suitability areas (black) for the occurrence of diploids and tetraploids of *Symphytum officinale* complex over studied region (average of 10 replicate MaxEnt runs, binary output).

Ten averaged runs of MaxEnt models for diploids and tetraploids had mean (±SD) AUC of ROC values of 0.926 (±0.038) and 0.863 (±0.045), respectively, showing very good predictive ability. The predicted distributions showed the nestedness of the distribution of diploids within that of tetraploids (Fig. 4), except for the Veneto and Po regions in northern Italy, where only diploids were predicted. Across the geographic range studied, the model showed high habitat suitability for diploids in several lowland or mid-elevation regions of Central and Western Europe, but avoidance of Northern and Eastern Europe, and most part of South Europe (Fig. 4A). Concerning tetraploids, the model showed high habitat suitability for tetraploids over most part of Western, Central and the western part of Eastern Europe, but also for the Southern Scandinavia (Fig. 4B).

Two variables that contributed the most to the average model for both diploids and tetraploids were SRAD and bio1 (Table 2). SRAD (mean annual solar radiation) and bio1 (annual mean temperature) had negative and positive effect, respectively, on the predicted probability of presence changes in both cytotypes **[see****Supporting Information—Fig. S1****]**. However, tetraploids were predicted to occur, though with a lower probability, even in areas with low mean annual temperature, while diploids were not. Although tetraploids were predicted to occur in a wide range of bio5 (maximal temperature of the warmest month), the model showed a lower suitability of low bio5 values for diploids **[see****Supporting Information—Fig. S1****]**. Regarding bio7 (temperature annual range), diploids were predicted to occur more likely at intermediate values, while tetraploids were predicted to occur in a wide range of low and intermediate values **[see****Supporting Information—Fig. S1****]**. Soil variables had a generally low percentual contribution to the average models for both cytotypes (Table 2). Only the volumetric percentage of coarse fragments (crfvol) in the soil had a positive effect on the suitability for both cytotypes **[see****Supporting Information—Fig. S1****]**.

### Ecological differences between cytotypes on the local spatial scale

The mean site EIVs for nutrients and salinity but not for light, temperature, moisture and soil reaction differed significantly between cytotypes **[see****Supporting Information—Fig. S2****]**. Diploids grow on heavier and more mineral-rich (salinity EIV, *F* = 9.388, *P* < 0.05) and nutrient-poorer (nutrients EIV, *F* = 22.278, *P* < 0.01) soils than tetraploids.

### Morphological differences between cytotypes

**Figure 5.**
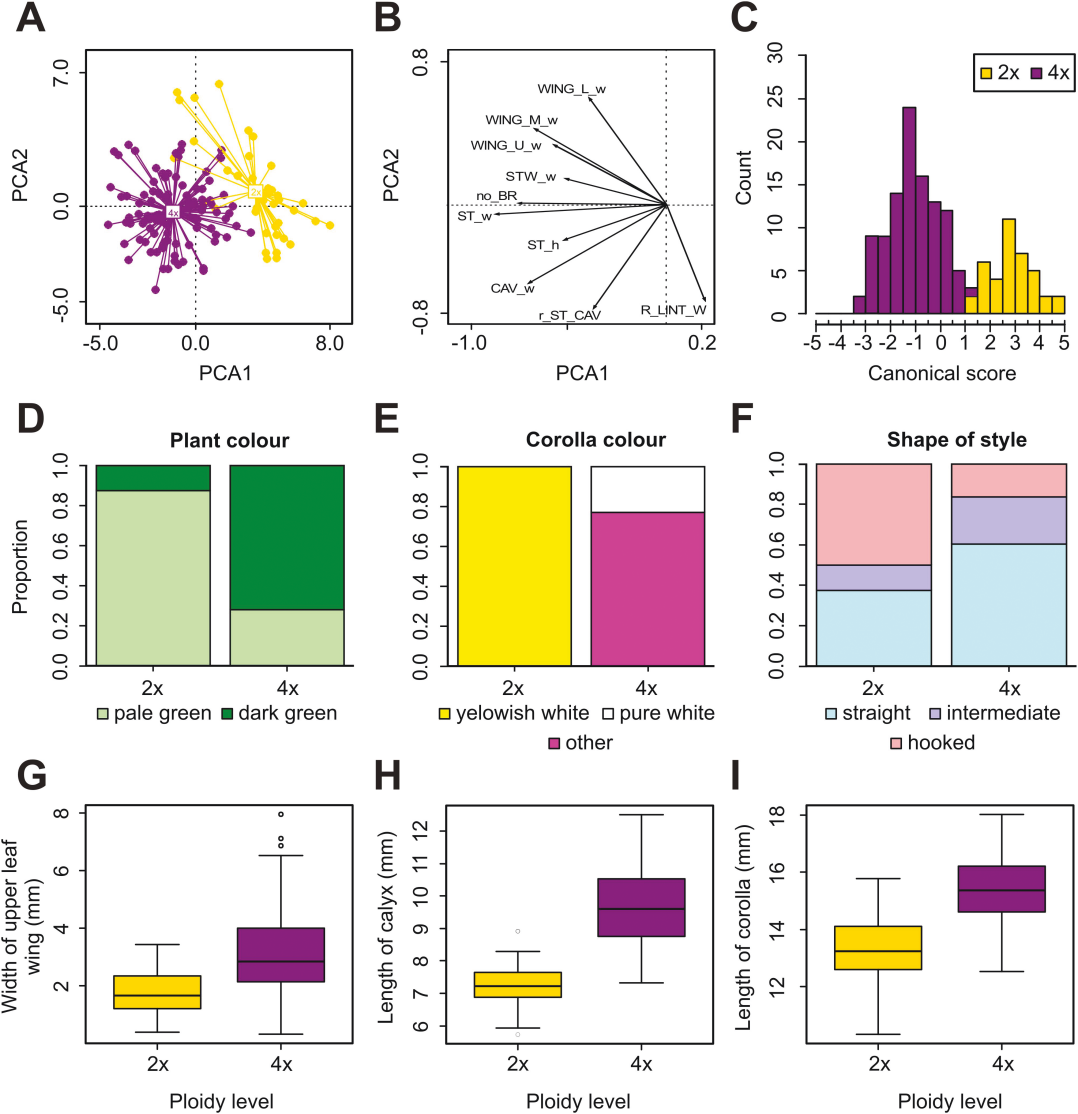
Morphological variation of diploids and tetraploids of the *Symphytum officinale* complex. (A) Spider plot PCA with individuals as OTUs (first and second axis explaining 23.1 % and 11.2 %, respectively, are displayed). (B) PCA of characters (for character abbreviations, see Table 3). (C) Histogram of canonical scores of CDA. (D–F) Percentage stacked bar charts of selected qualitative traits. (G–I) Representative box plots of selected quantitative morphological characters.

Principal component analysis revealed two groups distributed along the first axis corresponding to diploid and tetraploid cytotypes (Fig. 5A and B). The first two PCA axes explained 23.1 % and 11.2 % of the total variation. The colour of flowers and plants, calyx, corolla, peduncle, and style lengths and corolla width contributed most to the observed pattern (Fig. 5D–I). The cytotypes differed significantly from each other by 22 of 32 quantitative and all four qualitative morphological characters, and 15/4 characters remained significant even after Bonferroni correction, respectively (Table 2; see also **Supporting Information—Fig. S3**). However, the ranges of variation of all quantitative and qualitative (with one exception) characters overlap between cytotypes. Therefore, no single character can be used for the unambiguous determination of cytotypes except for the colour of flowers, where yellowish/greenish white corollas are confined solely to diploids, while pure white and all shades of purple corollas are confined to tetraploids (Fig. 5E). Canonical discriminant analysis resulted in a clear morphological separation between the two cytotypes (*F* = 11.692, *P* = 0.001; Fig. 5C). The contribution of individual characters to the observed pattern is given in **Supporting Information—Table S5**. The most important combination of characters for inclusion in one of the respective groups resulting from forward selection were length of calyx + plant colour + width of wing below lower leaf + length of peduncle + width of lowered part of corolla + width of wing below upper leaf + length/width ratio of middle leaf lamina.

## Discussion

### Cytotype diversity

Our flow cytometric ploidy screening and a review of published chromosome counts of *S. officinale* complex revealed the occurrence of 15 different chromosome counts with three main cytotypes, corresponding to diploids (2*n* = 24), hypotetraploids (2*n* = 40) and tetraploids (2*n* = 48).

As previously reported, the occurrence of triploids (2*n* = 36) is extremely rare, which is consistent with our discovery of only two triploids in a single diploid population. These two individuals probably resulted from the fusion of reduced and unreduced diploid gametes. Some of the few published reports of triploids (Strey 1931; Tischler 1935; Májovský 1974; Wille 1998) may not even be based on plants from *S. officinale* complex. As already noted by Gadella and Kliphuis (1972), at least some of these reports represent the hybrid taxon *S. × uplandicum* (*S. officinale* × *S. asperum*) or its backcrosses with one of the parental taxa. Thus, only reliable record of triploid occurrence besides our data is from mixed diploid–tetraploid population from the Netherlands (Basler 1972). The origin of these triploids may come from a cross between diploids and tetraploids as well as from a fusion of reduced and unreduced gametes of diploids.

Several other chromosome counts (2*n* = 54, 56) have also been published for *S. officinale* (Markowa and Iwanova 1970; Gadella and Kliphuis 1978; Gadella *et al.* 1983; van Loon 1987), although the origin of these plants is unclear (Gadella *et al.* 1983). Furthermore, aneuploid chromosome numbers ranging from 2*n* = 40 to 2*n* = 47 have been discovered in pure tetraploid populations (Gadella and Kliphuis 1967, 1978; Gadella *et al.* 1974; Shirato *et al.* 1985; Wille 1998). Similarly, the published chromosome record of 2*n* = 26 (Gadella and Kliphuis 1963) suggests the occurrence of aneuploidy in diploids; however, the same authors abandoned this view in their consequential studies and only reported the presence of B chromosomes (Gadella and Kliphuis 1967, 1970). Supernumerary B chromosomes have been repeatedly observed in the karyotype of diploids, occurring in various numbers (1–4; Gadella and Kliphuis 1967, 1970; Kamari *et al.* 2001; Peruzzi *et al.* 2001), and have never been identified in other cytotypes. However, since chromosomes of *S. officinale* complex are quite small (1.1–2.4 µm, Mekki *et al.* 1987), confusion with A chromosomes cannot be ruled out in other ploidy levels, particularly in tetraploids, where the aneuploid counts can, in fact, represent B chromosomes. Furthermore, the great variation in the nuclear DNA content within tetraploids was detected in our flow cytometric data (Fig. 2A), which may be caused by aneuploidy (reviewed in Šmarda and Bureš 2010) or the presence of B chromosomes. In some studies, the positive correlation between genome size and the presence of B chromosomes has even been found (Trivers *et al.* 2004; Levin *et al.* 2005). However, this variation could also be caused by other chromosomal polymorphisms (Greilhuber 1998) or differences in the content of repetitive DNA (Macas *et al.* 2015). Last but not least, methodological errors or the effect of secondary metabolites (Loureiro *et al.* 2006; Kolarčik *et al.* 2018; Kobrlová and Hroneš 2019) cannot be ruled out.

Genome size can serve as an additional tool for species identification and discrimination between closely related taxa (Zonneveld 2001; Suda *et al.* 2007; Prančl *et al.* 2014). Since AGS values estimated for diploids and tetraploids were non-overlapping (Fig. 2B), the nuclear DNA amount may be useful as a supportive marker for identification of morphologically problematic plants, e.g. white-flowered tetraploids, or plant determination in mixed population. So far, only three studies have been published considering the AGS of *S. officinale* complex (Veselý *et al.* 2013; Kobrlová and Hroneš 2019; Šmarda *et al.* 2019), and their results agree well with our estimates.

### Geographic distribution of cytotypes and population cytotype composition

Our study corroborates the common occurrence of tetraploids of the *S. officinale* complex in Europe, which is consistent with previously published chromosome counts **[see****Supporting Information—Table S2****]**. Compared to the broad geographic distribution of tetraploids, diploids have a scattered distribution throughout Europe and inhabit typically calcareous fens in floodplains and moist places in karst areas (Fig. 1D). The overall rarity of diploids may be caused by anthropogenic pressure and consequent loss of their habitats (Janssen *et al.* 2016) or by over competition by tetraploids (see below). We acknowledge that the distributional data presented here are partly geographically biased due to the lack of records from Eastern Europe, but clearly show the general large-scale distribution patterns of cytotypes of *S. officinale* complex. At the same time, it is possible that in some areas, diploids are overlooked and mistakenly associated with white-flowered tetraploids. In addition to these two major ploidy levels, hypotetraploids are even more scattered than diploids (except for the Netherlands) and occupy mineral-rich fens (Gadella and Kliphuis 1967, 1973; Gadella *et al.* 1983).

Geographic areas inhabited by two or more conspecific cytotypes are of particular interest (Mráz *et al.* 2008; Kolář *et al.* 2009; Duchoslav *et al.* 2020; Melichárková *et al.* 2021), providing the opportunity to study the evolutionary processes within polyploid complexes (Petit *et al.*, 1999; Kolář *et al.* 2017) and dynamics of ploidy coexistence (Čertner *et al.* 2017, 2022; Castro *et al.* 2018). Our results indicate that diploids and hypotetraploids occur primarily in mosaic regional parapatry (*sensu*Kolář *et al.* 2017) with tetraploids. Consequently, mixed-ploidy populations appear to be rare in *S. officinale* complex because out of 156, only three mixed diploid–tetraploid populations analysed by flow cytometry have been detected. At the same time, we have not detected triploids in these ploidy-mixed populations, so the possibility of gene flow appears to be excluded or extremely rare at present. This is consistent with previously published comprehensive cytotaxonomic studies of *S. officinale* complex in the Netherlands with no triploids detected in mixed diploid–tetraploid populations, indicating the existence of a strong reproductive barrier between these cytotypes (Gadella and Kliphuis 1967, 1972). The only exception is the study of Basler (1972), who detected two triploids (both white-flowered) in mixed diploid–tetraploid population in the Schleswig-Holstein region, Northern Germany. However, their origin has never been confirmed by molecular or experimental methods. Similarly, the crossing experiments resulted in extremely low reciprocal cross-ability of diploids and white-flowered tetraploids of Dutch origin, with only two triploids produced (i.e. 0.1 %), which were not able of flowering and thus producing viable seeds (Gadella and Kliphuis 1969; Gadella 1972).

### Niche differentiation between cytotypes at various spatial scales

Adaptation of newly established autopolyploids to new ecological niches is considered as a way to avoid competition with their diploid ancestors and consequently an important speciation mechanism (Fowler and Levin 1984). Therefore, it is hypothesized that polyploids will have wider niches and be better adapted to the abiotic extremes (Levin 2002) and this hypothesis was supported by several studies (Spoelhof *et al.* 2017; Baniaga *et al.* 2020). However, the niches of diploids and autopolyploids may differ (Arnold *et al.* 2015; Visger *et al.* 2016), but also overlap or even be equivalent (Godsoe *et al.* 2013; Casazza *et al.* 2016; Duchoslav *et al.* 2020). We found slight differences from niche equivalency between diploids and tetraploids of *S. officinale* complex. Visger *et al.* (2016) argued that even minor differences from niche equivalency in autopolyploids may be important for escape from the minority cytotype exclusion process. However, we also found that tetraploids have a much wider niche than diploids, with the niche of diploids almost entirely contained within the tetraploid niche. Moreover, observed shift towards more extreme abiotic conditions in tetraploids is pronounced by their tendency to occupy also colder areas with lower precipitations and their ability to inhabit also mineral-poor, sandy soils. Better tolerance to lower mean temperatures in polyploids is a commonly reported trait that strengthens their frequent occurrence at higher latitudes and/or altitudes (Husband *et al.* 2013; Rice *et al.* 2019).

Incorporating data from the local scale also suggests that tetraploids prefer nutrient-richer soils that are frequently associated with both natural and anthropically disturbed sites, such as gravel bars, riverbanks, road edges and various types of perennial ruderal vegetation on moist soils, as shown by Kobrlová (2017), who analysed data on habitat conditions of both cytotypes extracted from herbarium sheets collected in the Czech Republic. Tetraploids can be thus viewed as more generalist with tendency to occupy also places with higher nutrient (N, P) content, while diploids are a little bit more specialized to mineral-richer soils. This perfectly fits with estimated indices of ecological specialization for Czech flora (Zelený and Chytrý 2019) with diploids (i.e. *S. bohemicum*) being more specialized than tetraploids (*S. officinale*). The stronger synanthropic affinity of polyploids, in contrast to their diploid congeners, has recently been reported in several polyploid complexes (Zozomová-Lihová *et al.* 2014; Chung *et al.* 2015; Němečková *et al.* 2019; Rejlová *et al.* 2019; Urfus *et al.* 2021). Alternatively, observed pattern in niche differentiation between diploids and tetraploids may result from stronger competitive ability of tetraploids, unless they are confined to nutrient-poor and/or more-stressful substrates that do not allow the tetraploids to exploit their advantage.

### Morphological variation of *S. officinale* in the Czech Republic

There has been a long-lasting debate about the taxonomical identity of diploids that are exclusively ‘white-flowered’, but in most European floras no or only negligible taxonomic significance is attributed to them (Table 1). Most of the authors consider diploids to be morphologically indistinguishable from tetraploids, belonging to one polymorphic species *S. officinale* s. str. This is evidenced by Basler (1972), who provided a morphological evaluation of both cytotypes in the Schleswig-Holstein region (Northern Germany) and did not treat diploids and tetraploids as separate taxa; the only detected significant differences he found were some microscopic features (pollen, stomata, cell size). However, Wille (1998), evaluating a morphological variation of *S. officinale* complex in Southern and Central Hesse (Central Germany), distinguishes between tetraploids (*S. officinale* s. str.) and diploids (*S. bohemicum*) quite well, although not every individual can be unequivocally identified by its morphological characteristics. This difficulty can be easily avoided by evaluating the whole population (Kobrlová 2017). Rather surprisingly, within the framework of the long-time study of *S. officinale* complex provided in the Netherlands, authors never considered diploids as a separate taxon, but only as a morphotype of *S. officinale* (Gadella and Kliphuis 1967, 1972; Gadella *et al.* 1970, 1983; Gadella 1972).

Our results clearly show that diploids and tetraploids are morphologically distinct. The best morphological characters to discriminate between these two cytotypes are the colour of flowers and plants, the width of the wing below lower and upper leaf, the length/width ratio of the middle leaf lamina, the calyx, corolla, peduncle, and style lengths and corolla width (Fig. 5D–I; **see****Supporting Information—Fig. S3**). This morphological pattern has been observed within all investigated European populations, but it has been examined in more detail only within the Czech populations, due to a limited sampling outside the Czech Republic. The corollas of diploids are always yellowish to greenish white, never pure white as reported in most works (Gadella and Kliphuis 1967, 1969; Basler 1972; Peruzzi *et al.* 2001). In contrast, pure white corollas are rarely and randomly found only in tetraploids (Fig. 5E) and have never been observed in hypotetraploids of *S. tanaicense* (Gadella and Kliphuis 1973; Smejkal 1978; Májovský and Hegedüšová 1993; Peruzzi *et al.* 2001). The ignorance of the corolla colour differences (yellowish white vs. pure white) might stand behind the long-lasting neglection of diploids as a separate taxon. The combination of quantitative and qualitative morphological traits presented here has previously been successfully applied by the first author during the revision of herbarium vouchers of *S. officinale* complex in the Czech Republic (Kobrlová 2017).

In contrast, less abundant hypotetraploids that have flower colour similar to tetraploids (dark purple or purplish-violet) are distinguished as a separate taxon by most of the authors. Based on previous studies, they differ by generally unbranched stems, not or only very shortly decurrent leaves, both sparsely hairy to almost glabrous, and calyx lobes with long hairs along margins and at midribs (Gadella and Kliphuis 1973; Smejkal 1978; Májovský and Hegedüšová 1993; Peruzzi *et al.* 2001). The taxonomic treatment of polyploids, especially autopolyploids, has often been controversial, and different taxonomists may have various criteria (Soltis *et al.* 2007). On the basis of our findings, the morphological differentiation of all three major cytotypes is obvious and appears to be taxonomically significant, especially in combination with slight ecological segregation and the apparent presence of hybridization barriers (Gadella and Kliphuis 1969).

### Taxonomic implications

We showed that both major cytotypes of the *S. officinale* complex are morphologically well-differentiated. Although we did not morphologically evaluate diploids from other parts of their range, our field observations confirm that the diploids are readily distinguishable from tetraploids. As the different ploidy level act as a strong mating barrier, as indicated by rare occurrence of triploids, both cytotypes should be treated as separate species. As far as we know, the oldest validly published name for diploids is *S. bohemicum* (Kirschner *et al.* 2007). Subsequent studies should focus on the evolutionary pathways of the origin of the tetraploid cytotype (single or multiple, which may explain its broader niche) and also on detailed revision (morphology, ecology, etc.) of hypotetraploids, in connection with the name *S. tanaicense*, to support the taxonomic value of this species. The relationships between all cytotypes should be examined also by molecular approaches to shed light on the evolution of this complex and to clarify its taxonomic concept.

## Supporting Information

The following additional information is available in the online version of this article—

Table S1. Locality details for 156 *Symphytum officinale* agg. populations, including number of analysed plants, information on which populations have been subjected to morphometric analysis, detected ploidy level and detailed results of flow cytometry analyses.

Table S2. List of revised karyological data reported for *Symphytum officinale* complex.

Table S3. Relative DNA content of *Symphytum officinale* complex assessed using flow cytometry; propidium iodide (PI) and 4,6-diamidino-2-phenylindole (DAPI) staining.

Table S4. The genome size (2C value) of the *S. officinale* cytotypes.

Table S5. Total canonical structure expressing correlations of traits with canonical axes in discriminate analysis (CDA). Values that exceed the level of 0.2 are in bold.

Figure S1. Response curves that show how each environmental variable affects the MaxEnt prediction.

Figure S2. Variation of the mean site Ellenberg-type indicator values (EIVs) derived from phytocoenological relevés with results of one-way ANOVA with the modified permutation test with 499 permutations.

Figure S3. Box plots of quantitative morphological characters and their ratios. Box plot body define the 25th and 75th percentiles, horizontal lines show the median, whiskers are from the 10 to 90 percentiles, circles show extreme values.

plac028_suppl_Supplementary_Materials

## Data Availability

The original contributions presented in the study are included in the article/**Supporting Information**; further inquiries can be directed to the corresponding author.

## Notes

**Evolution & Diversity. Chief Editor**: Jeremy Beaulieu
