# Supplementary material for: Morphological, ecological and geographic differences between diploids and tetraploids of Symphytum officinale (Boraginaceae) justify both cytotypes as separate species
Source: AoB Plants. 2022 Jun 21;14(4):plac028. doi: 10.1093/aobpla/plac028 (PMC9297162; doi:10.1093/aobpla/plac028)
Supplement: plac028_suppl_Supplementary_Materials [file plac028_suppl_supplementary_materials.zip › Kobrlova_Symphytum_Supporting_Information_S5-S8.pdf]

Supporting Information

Article title: Morphological, ecological and geographic differences between diploids and tetraploids of *Symphytum officinale* (Boraginaceae) justify both cytotypes as separate species

Authors: Lucie Kobrlová, Martin Duchoslav & Michal Hroneš

Figure S5. Response curves that show how each environmental variable affects the Maxent prediction.

Below response curves of selected environmental variables for diploids (2*x*) and tetraploids (4*x*) of *Symphytum officinale* s.l. are reported. The curves show the mean response of the 10 replicate Maxent runs (red) and ± 1 SD (blue).

Environmental variable (abbreviation, x-label in the graphs)

Mean annual solar radiation (SRAD; SRAD)
Annual Mean Temperature (bio1; wcbio1)
Max Temperature of Warmest Month (bio5; wcbio5)
Temperature Annual Range (bio7; wcbio7)
Precipitation Seasonality (bio15; wcbio15)
Volumetric percentage of coarse fragments (>2 mm) (crfvol; crfvol_sr2)

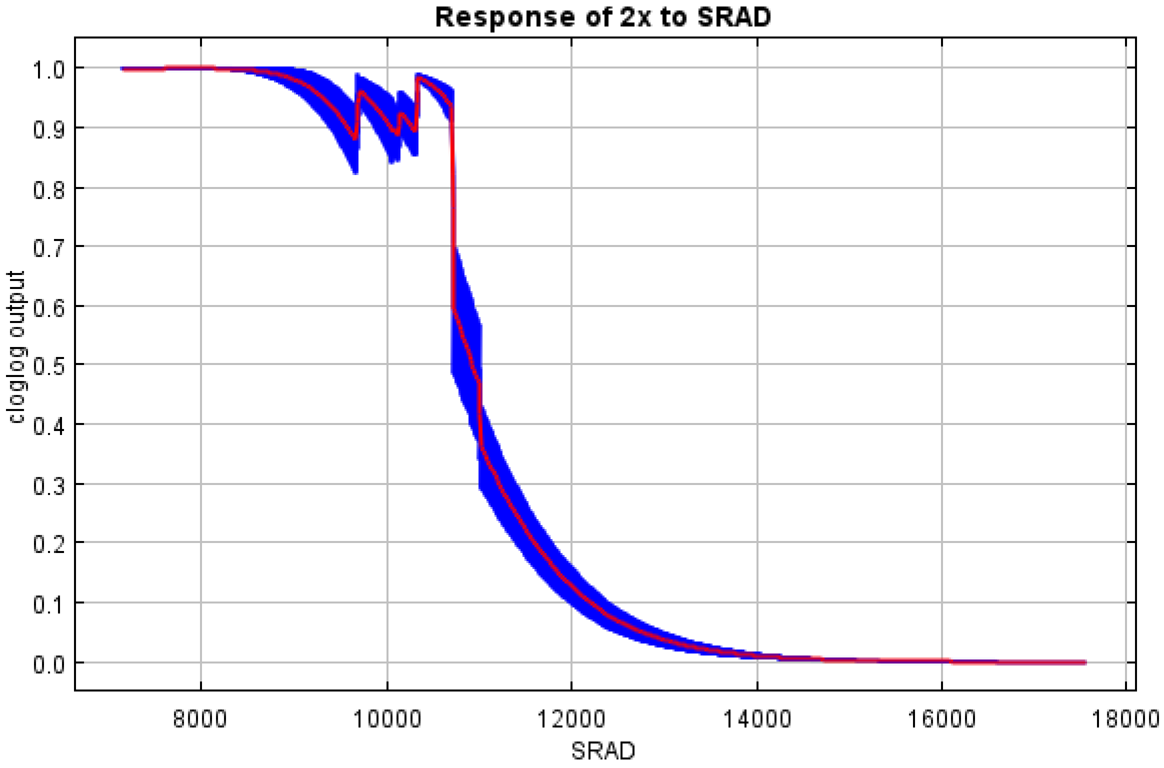
Response of 2x to SRAD
cloglog output
1.0
0.9
0.8
0.7
0.6
0.5
0.4
0.3
0.2
0.1
0.0
8000
10000
12000
14000
16000
18000
SRAD

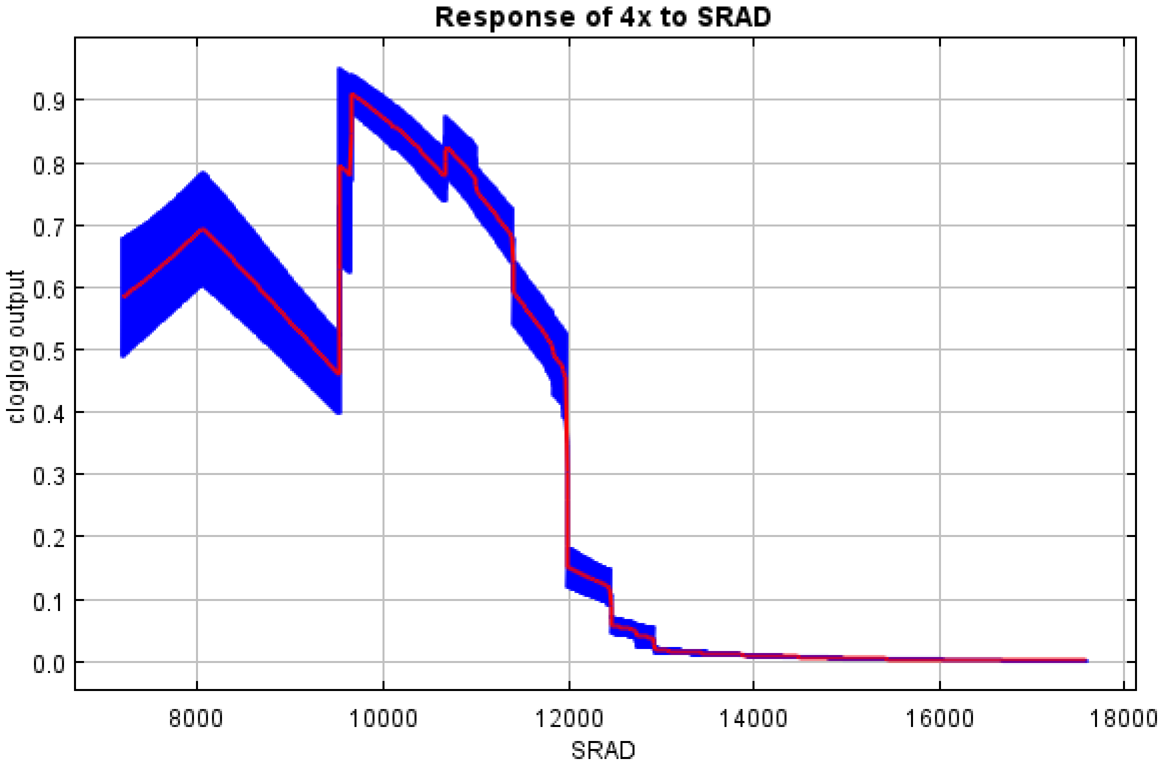
Response of 4x to SRAD
cloglog output
0.9
0.8
0.7
0.6
0.5
0.4
0.3
0.2
0.1
0.0
8000
10000
12000
14000
16000
18000
SRAD

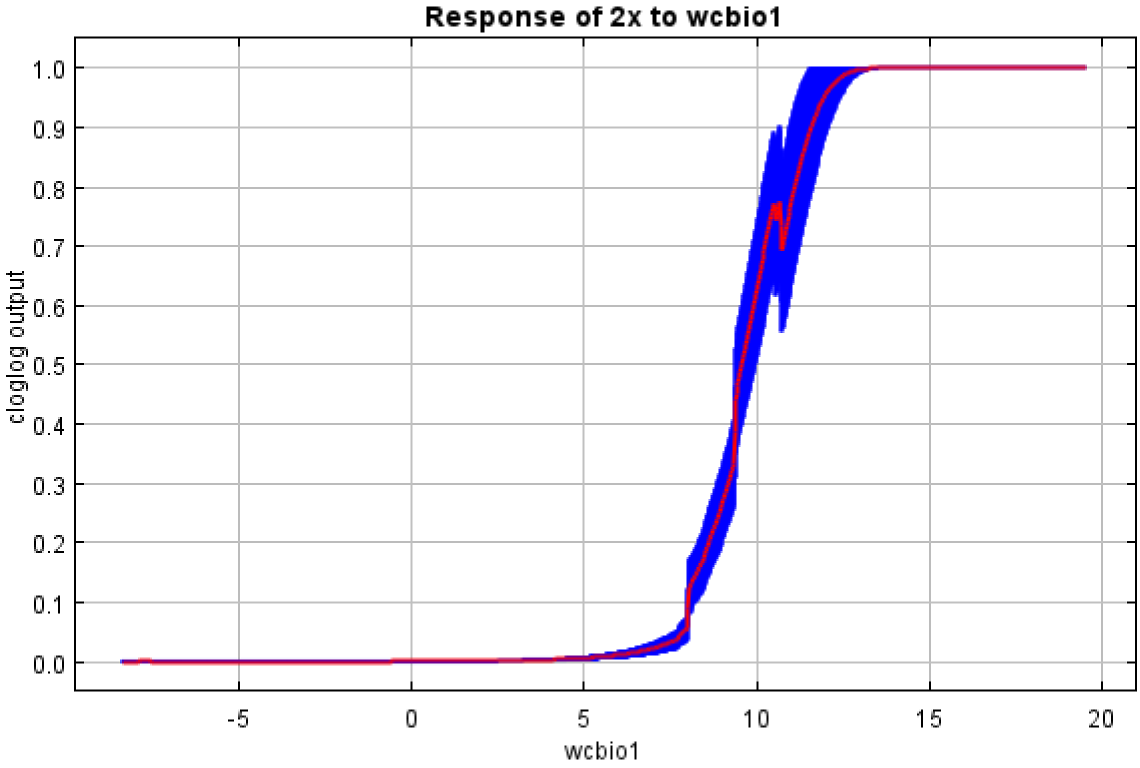

Response of 2x to wcbio1
cloglog output
1.0
0.9
0.8
0.7
0.6
0.5
0.4
0.3
0.2
0.1
0.0
-5
0
5
10
15
20
wcbio1

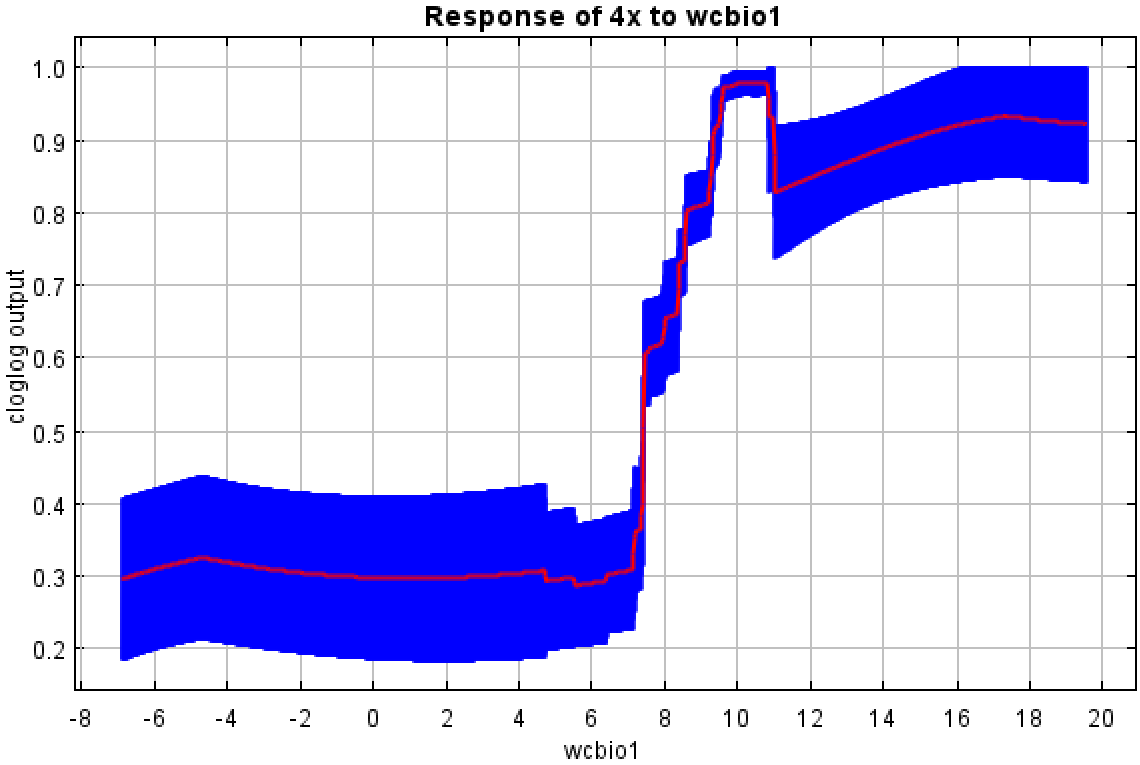

Response of 4x to wcbio1
cloglog output
1.0
0.9
0.8
0.7
0.6
0.5
0.4
0.3
0.2
-8
-6
-4
-2
0
2
4
6
8
10
12
14
16
18
20
wcbio1

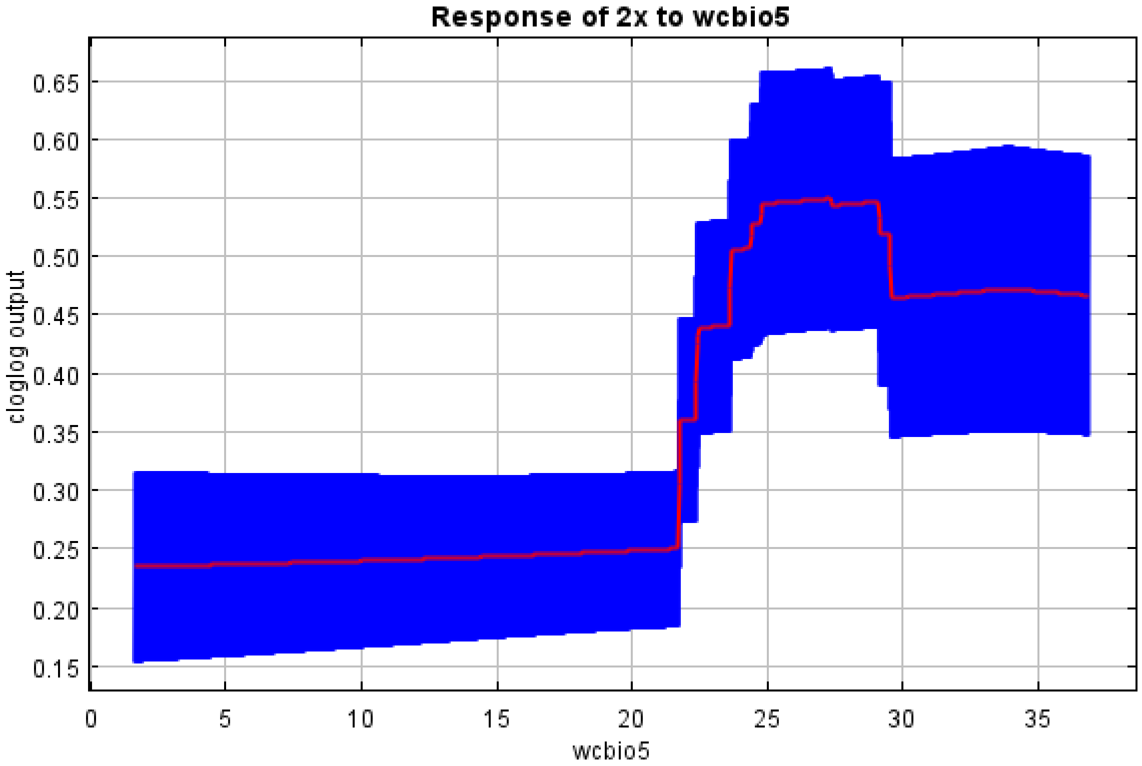

Response of 2x to wcbio5
cloglog output
0.65
0.60
0.55
0.50
0.45
0.40
0.35
0.30
0.25
0.20
0.15
0
5
10
15
20
25
30
35
wcbio5

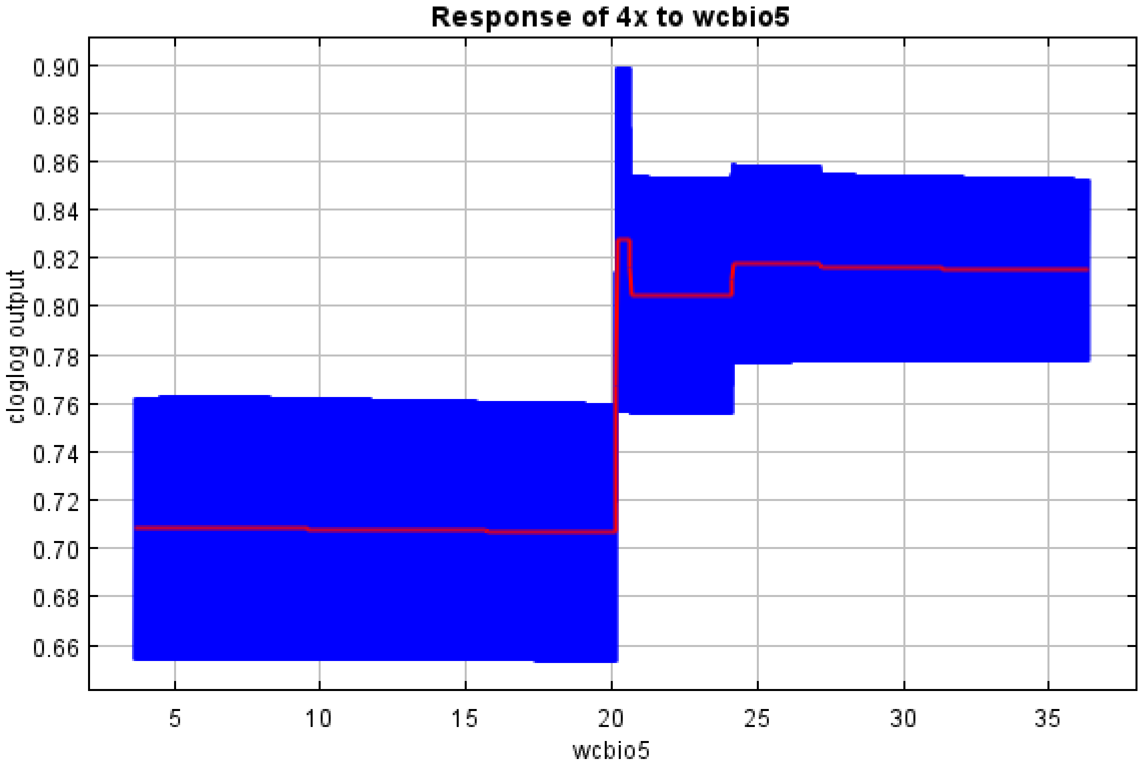

Response of 4x to wcbio5
cloglog output
0.90
0.88
0.86
0.84
0.82
0.80
0.78
0.76
0.74
0.72
0.70
0.68
0.66
5
10
15
20
25
30
35
wcbio5

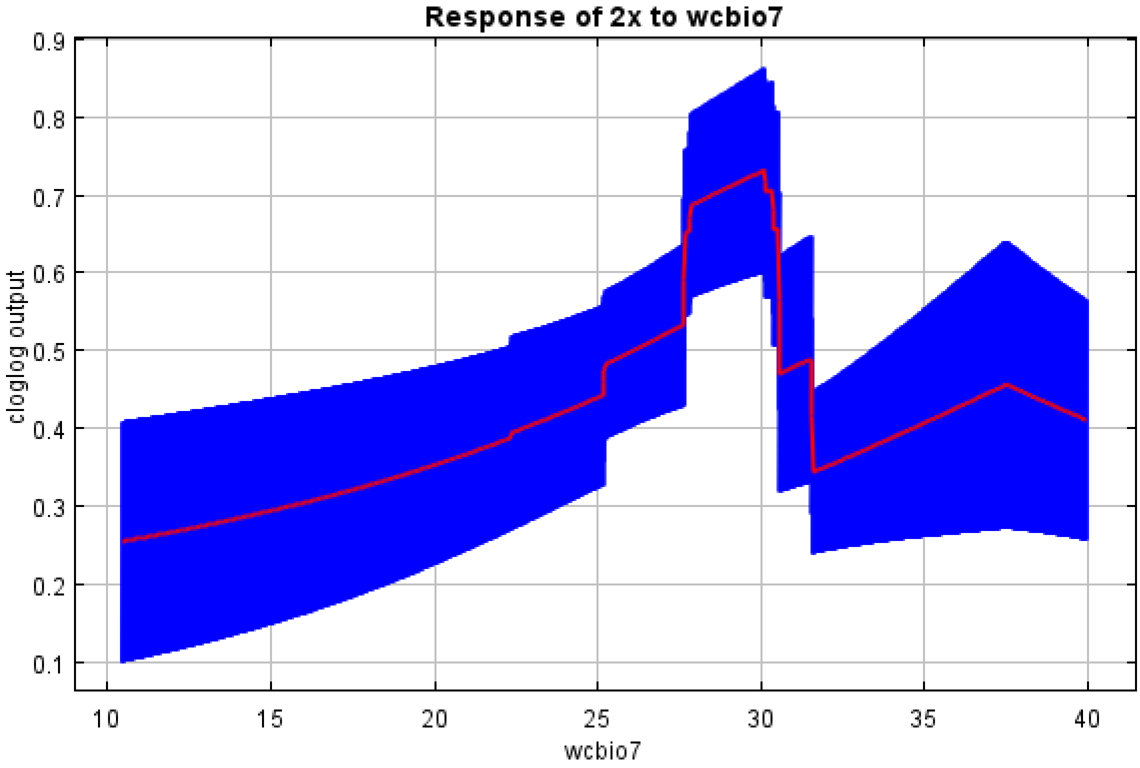

Response of 2x to wcbio7
cloglog output
wcbio7
0.1
0.2
0.3
0.4
0.5
0.6
0.7
0.8
0.9
10
15
20
25
30
35
40

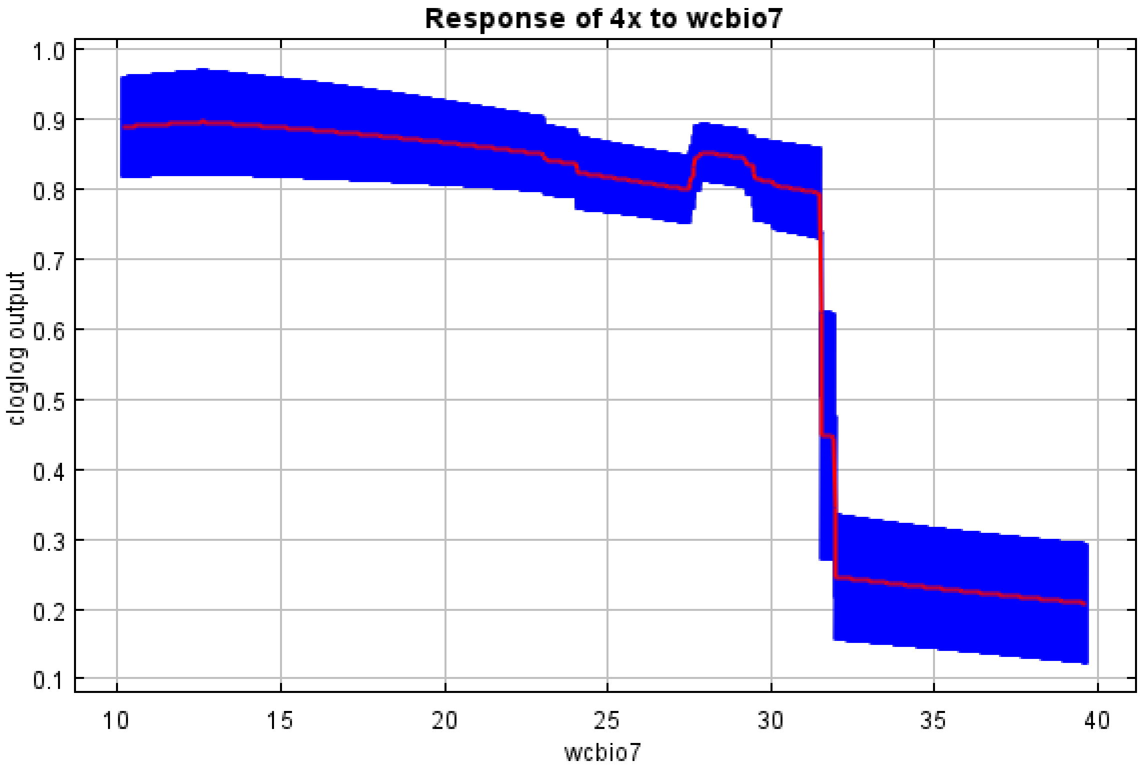

Response of 4x to wcbio7
cloglog output
wcbio7
0.1
0.2
0.3
0.4
0.5
0.6
0.7
0.8
0.9
1.0
10
15
20
25
30
35
40

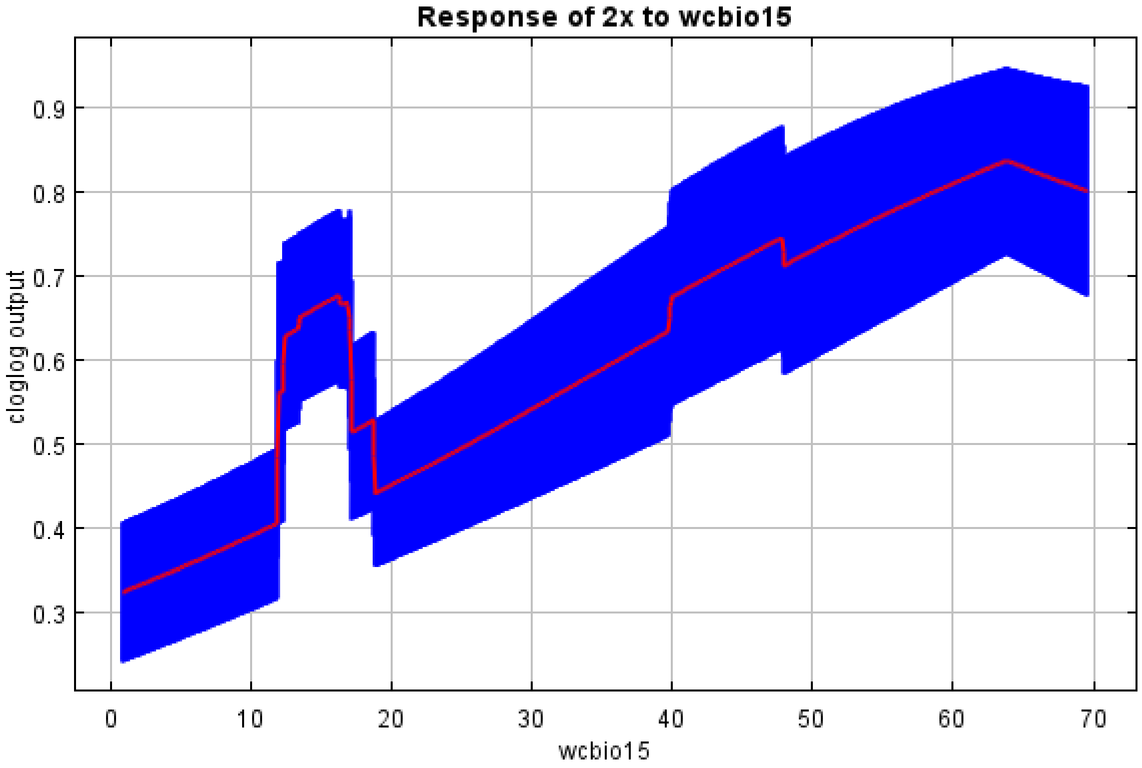
Response of 2x to wcbio15
cloglog output
0.3
0.4
0.5
0.6
0.7
0.8
0.9
0
10
20
30
40
50
60
70
wcbio15

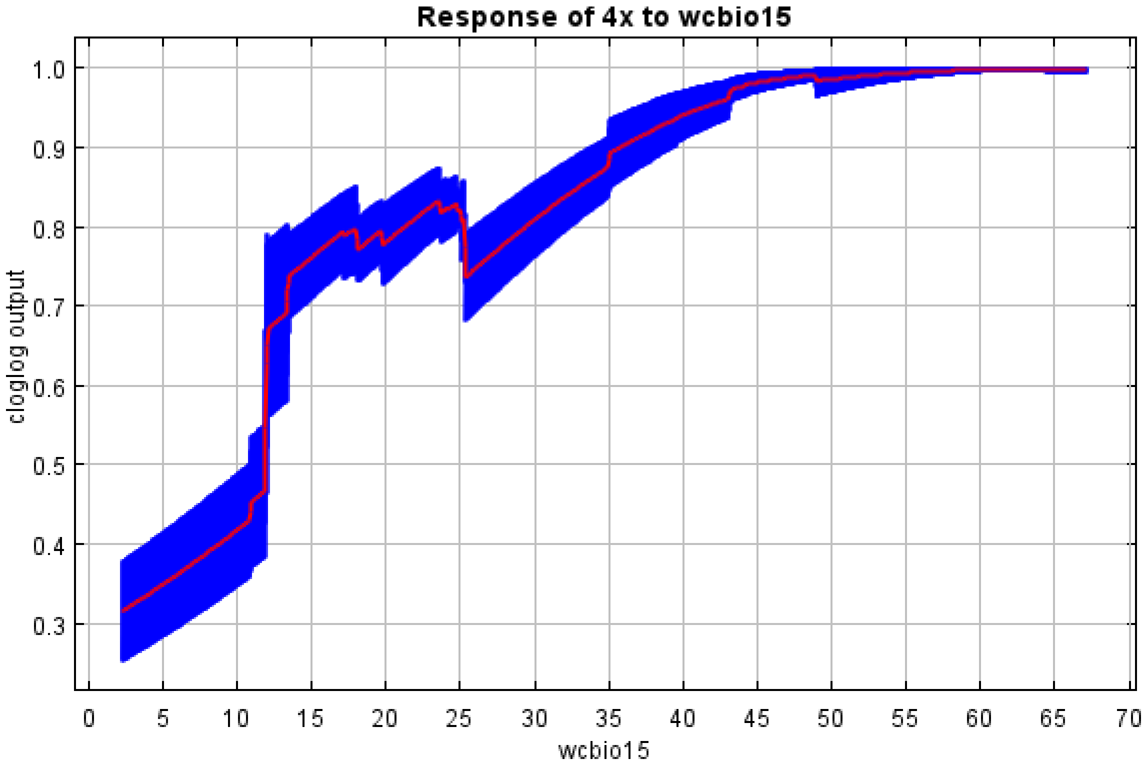
Response of 4x to wcbio15
cloglog output
0.3
0.4
0.5
0.6
0.7
0.8
0.9
1.0
0
5
10
15
20
25
30
35
40
45
50
55
60
65
70
wcbio15

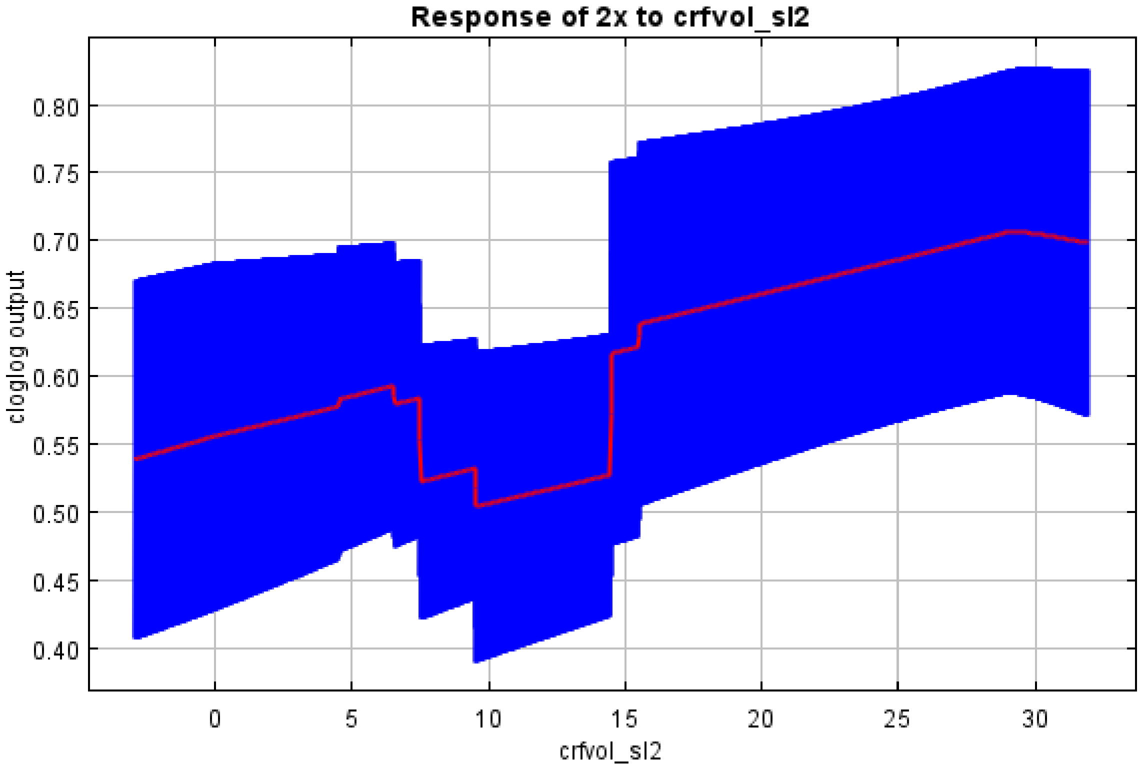
Response of 2x to crfvol_sl2
cloglog output
crfvol_sl2
0.40
0.45
0.50
0.55
0.60
0.65
0.70
0.75
0.80
0
5
10
15
20
25
30

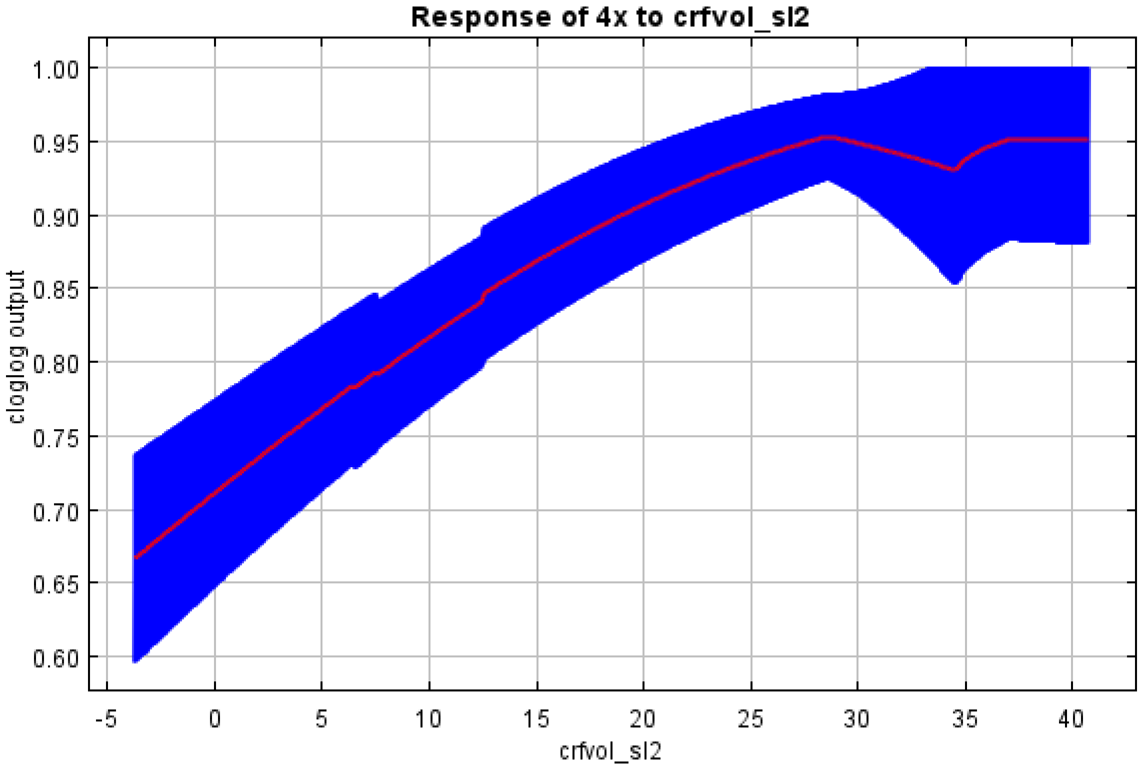
Response of 4x to crfvol_sl2
cloglog output
crfvol_sl2
0.60
0.65
0.70
0.75
0.80
0.85
0.90
0.95
1.00
-5
0
5
10
15
20
25
30
35
40

Figure S6. Variation of the mean site EIVs derived from phytocoenological relevés with results of one-way ANOVA with the modified permutation test with 499 permutations.

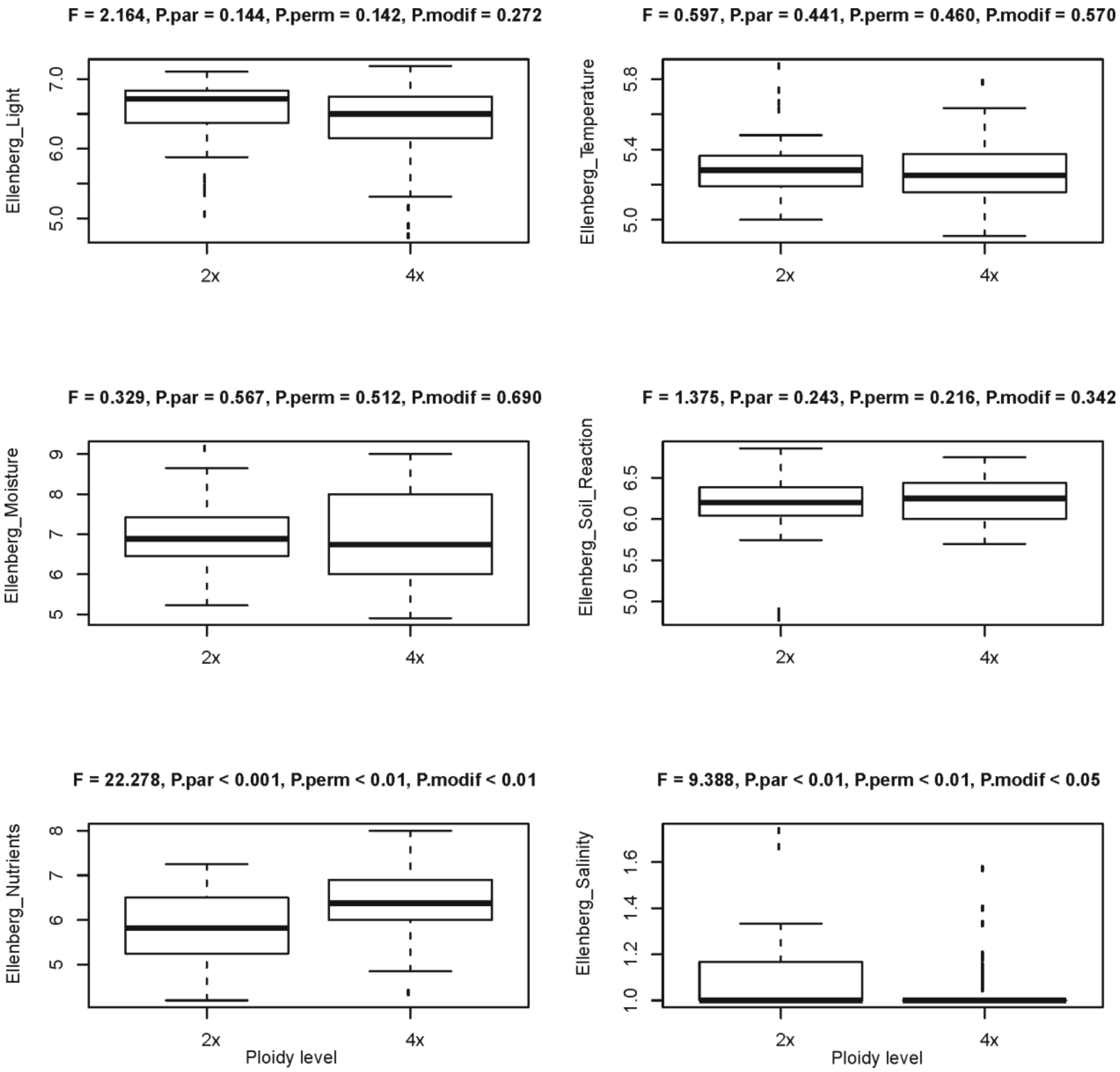

Figure S7. Box plots of quantitative morphological characters and their ratios. Box plot body define the 25th and 75th percentiles, horizontal lines show the median, whiskers are from the 10 to 90 percentiles, circles show extreme values).

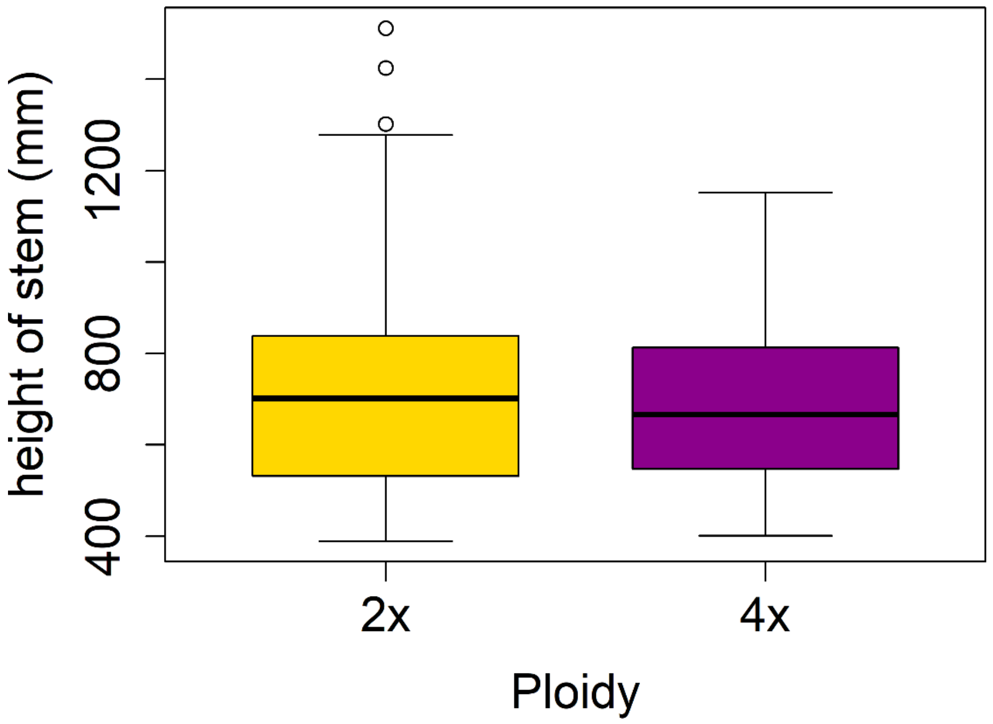

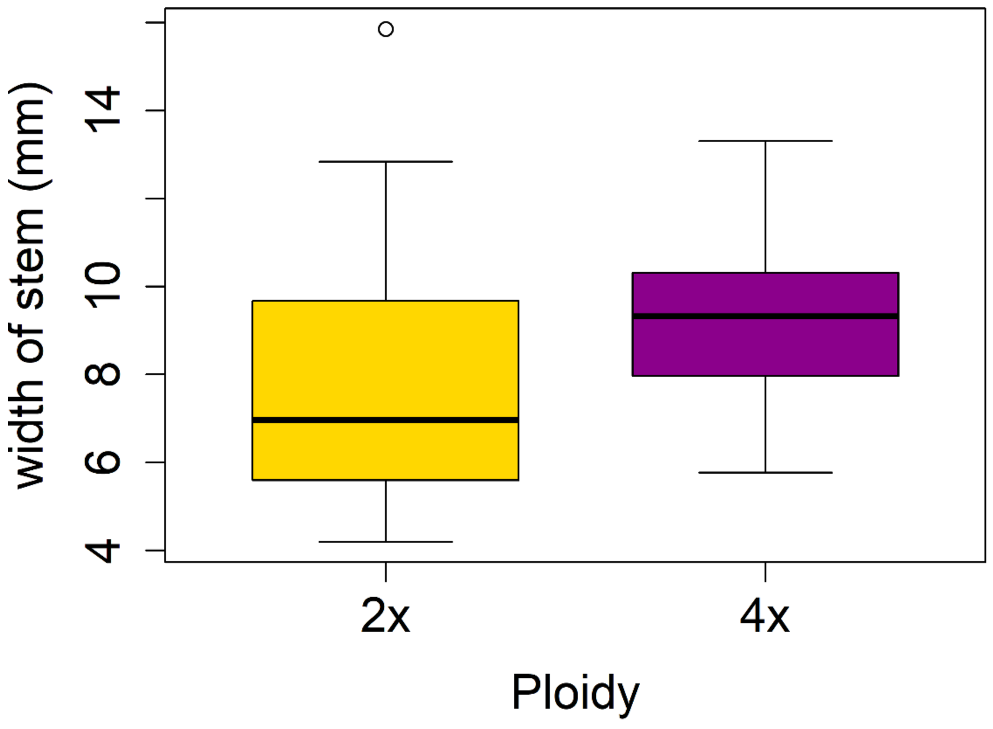

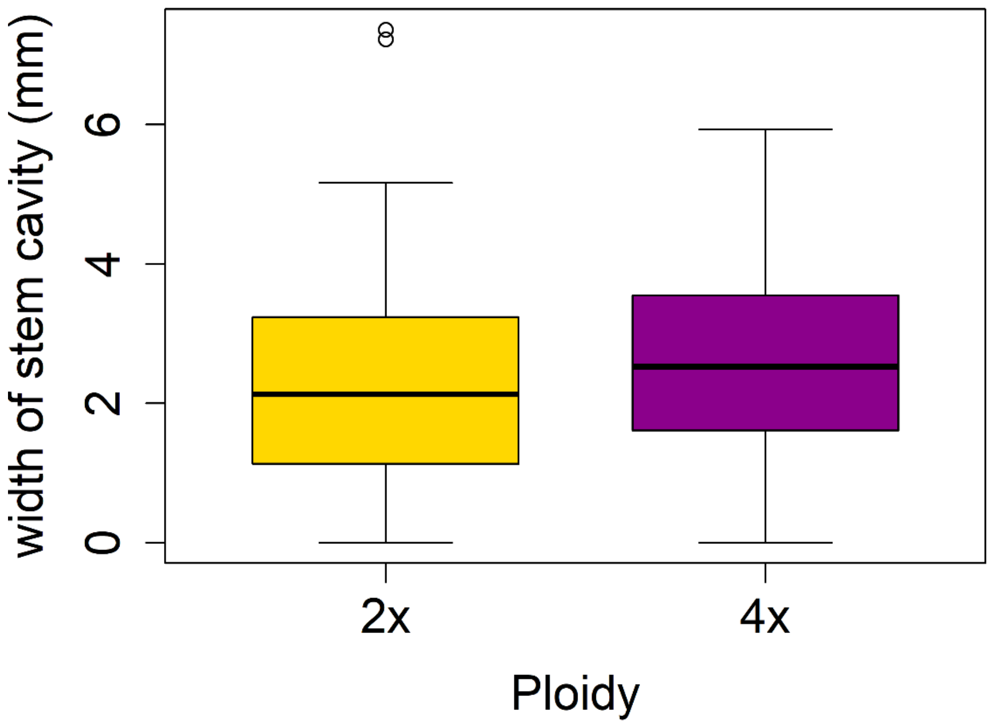
width of stem cavity (mm)
0
2
4
6
2x
4x
Ploidy

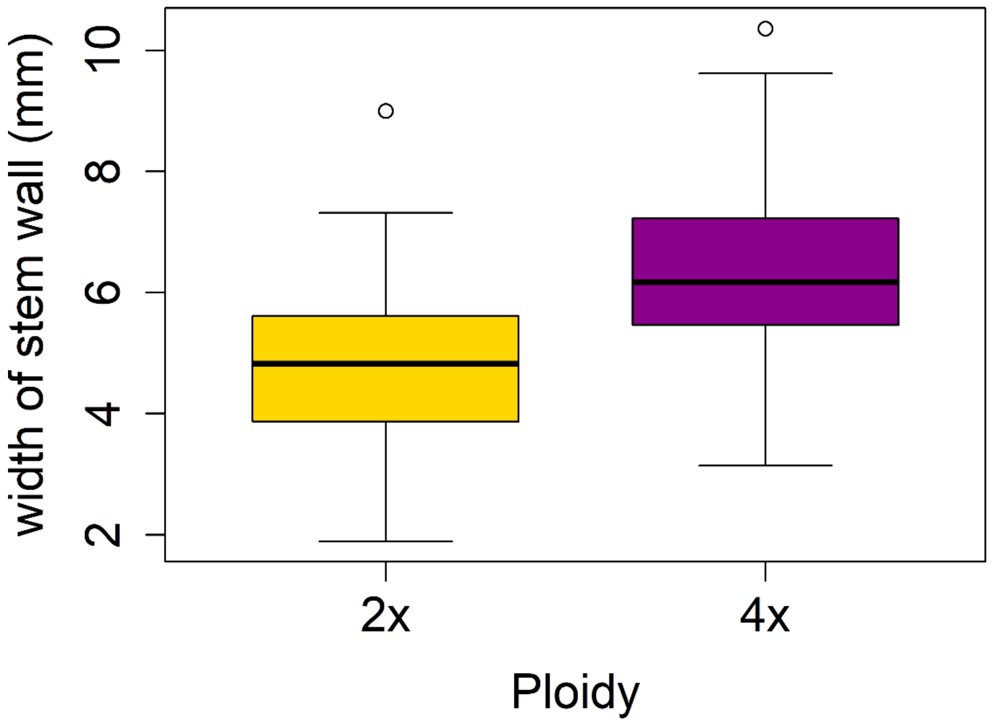
width of stem wall (mm)
2
4
6
8
10
2x
4x
Ploidy

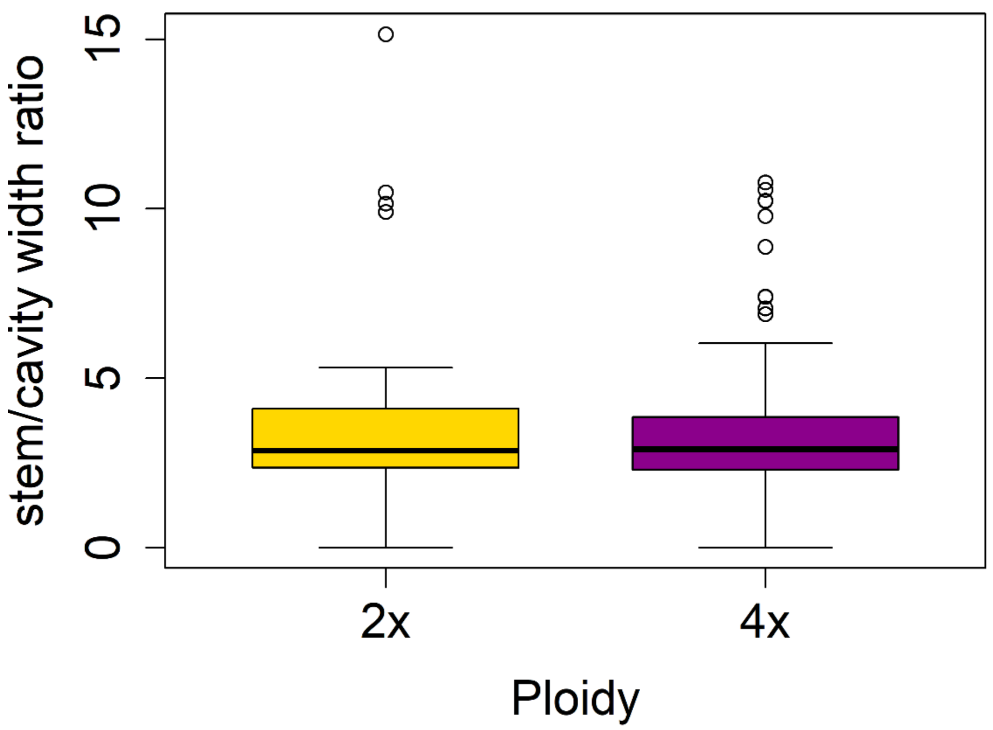

stem/cavity width ratio
0
5
10
15
2x
4x
Ploidy

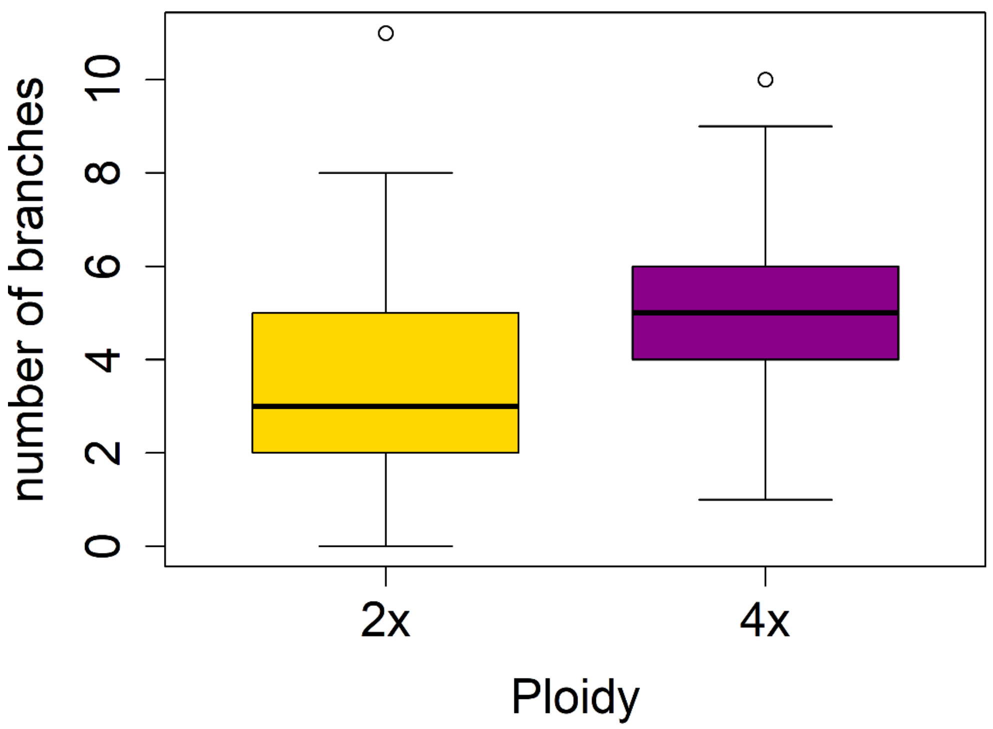

number of branches
0
2
4
6
8
10
2x
4x
Ploidy

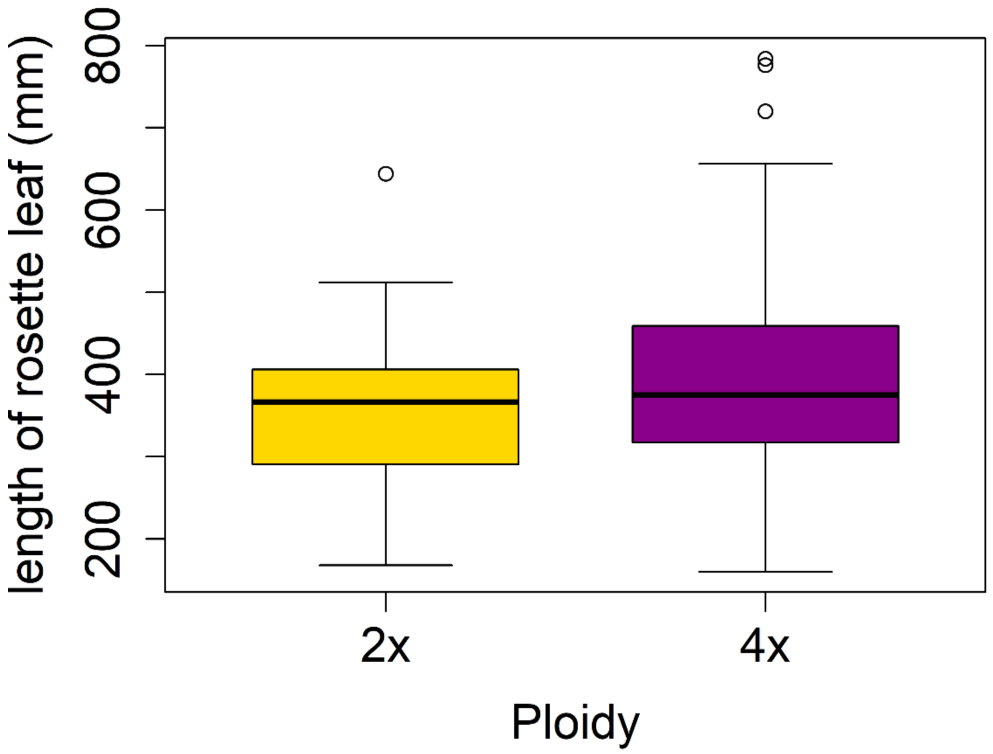

length of rosette leaf (mm)
200
400
600
800
2x
4x
Ploidy

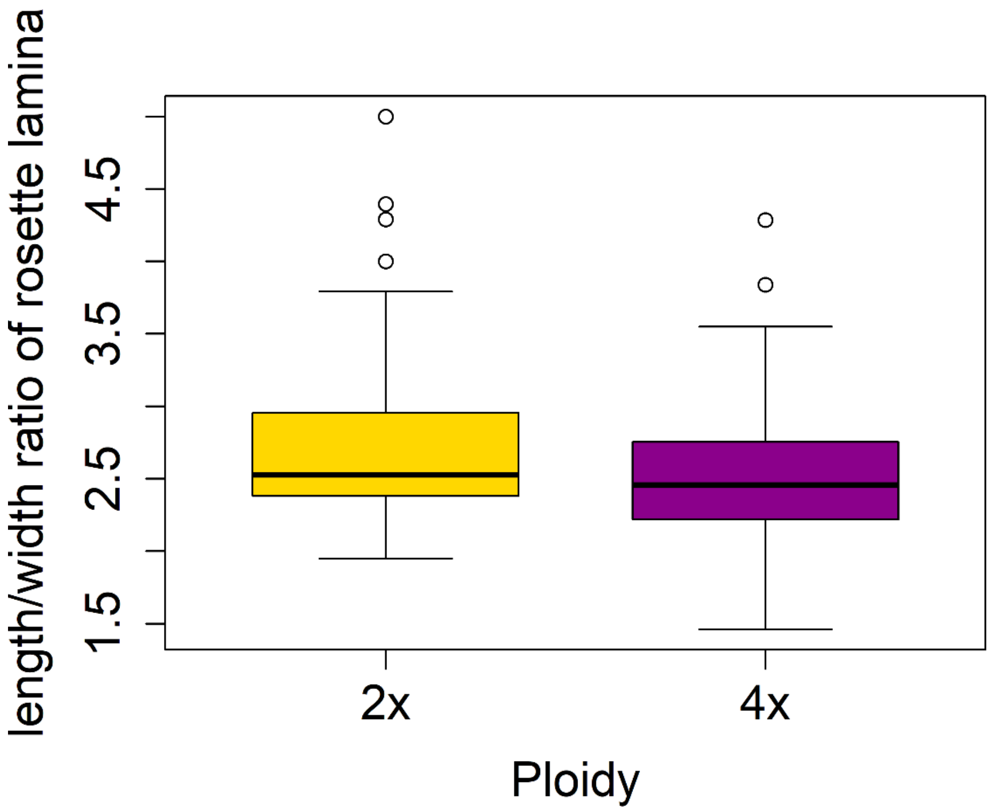

length/width ratio of rosette lamina
1.5
2.5
3.5
4.5
2x
4x
Ploidy

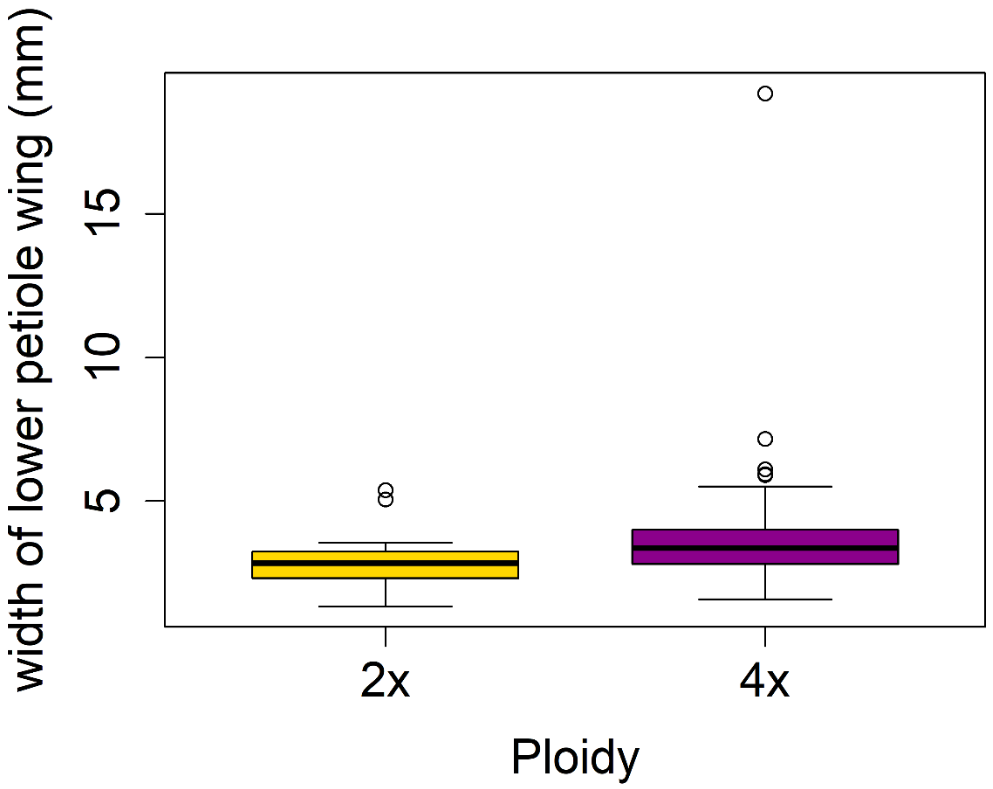
width of lower petiole wing (mm)
5
10
15
2x
4x
Ploidy

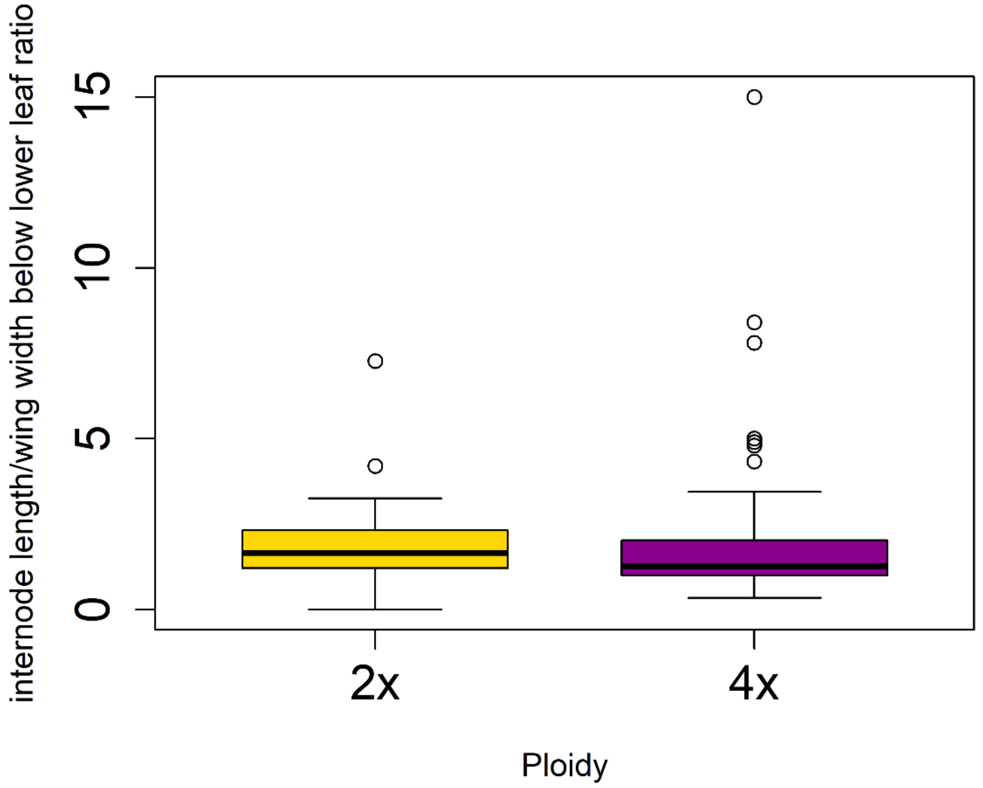
internode length/wing width below lower leaf ratio
0
5
10
15
2x
4x
Ploidy

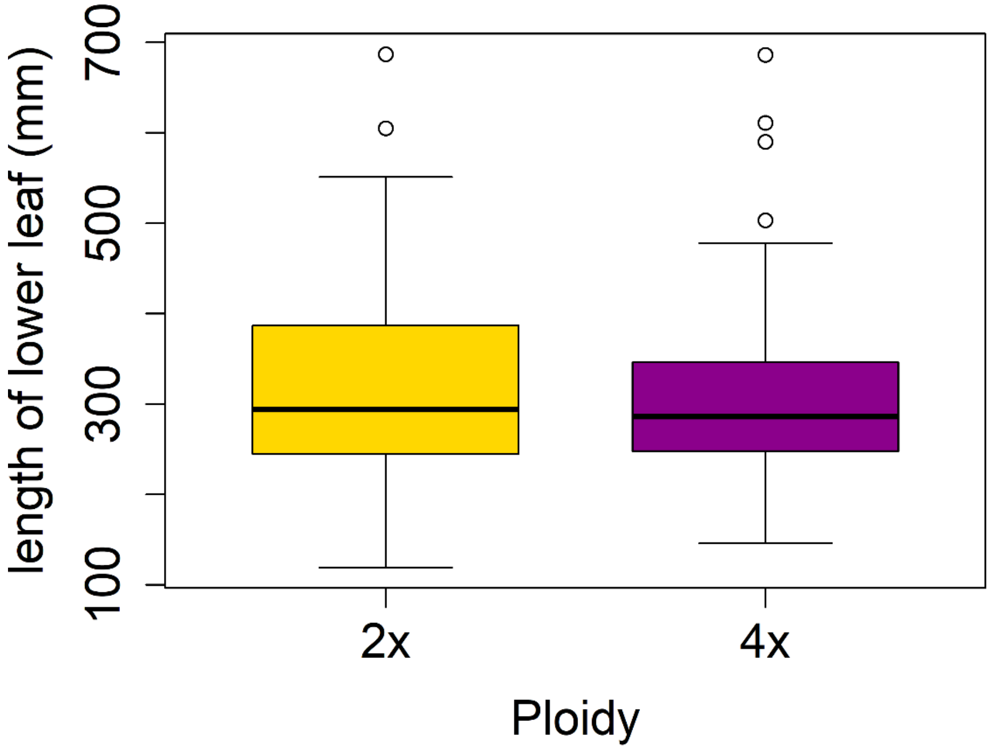

length of lower leaf (mm)
100
300
500
700
2x
4x
Ploidy

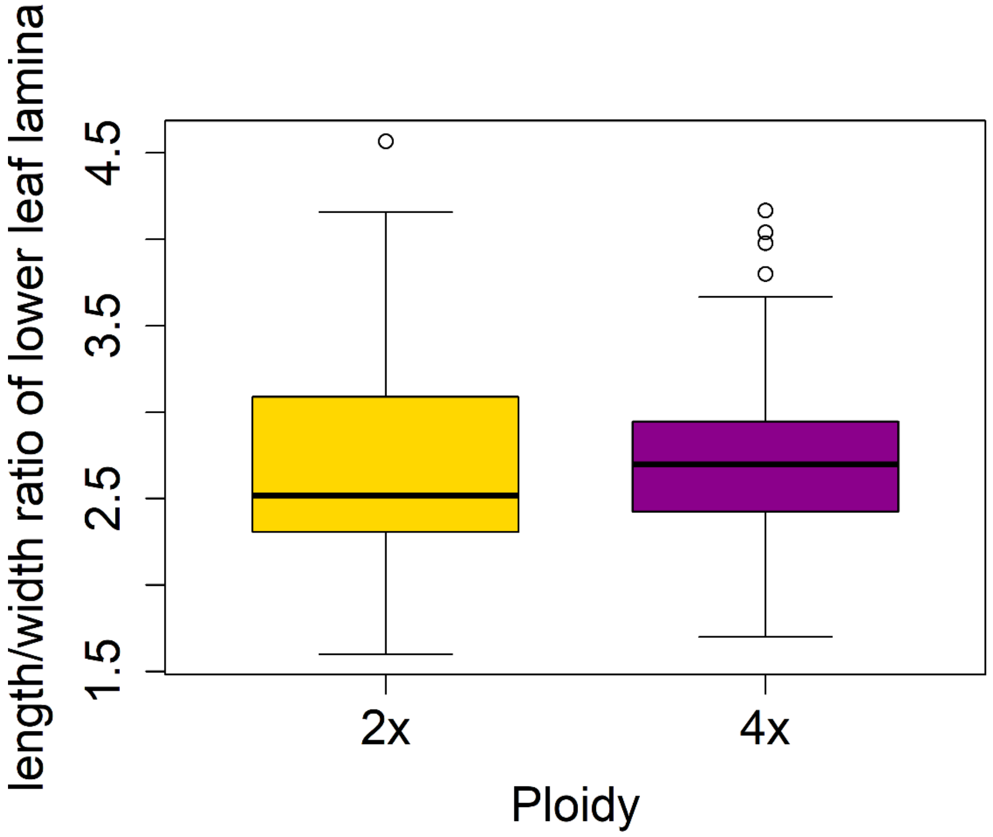

length/width ratio of lower leaf lamina
1.5
2.5
3.5
4.5
2x
4x
Ploidy

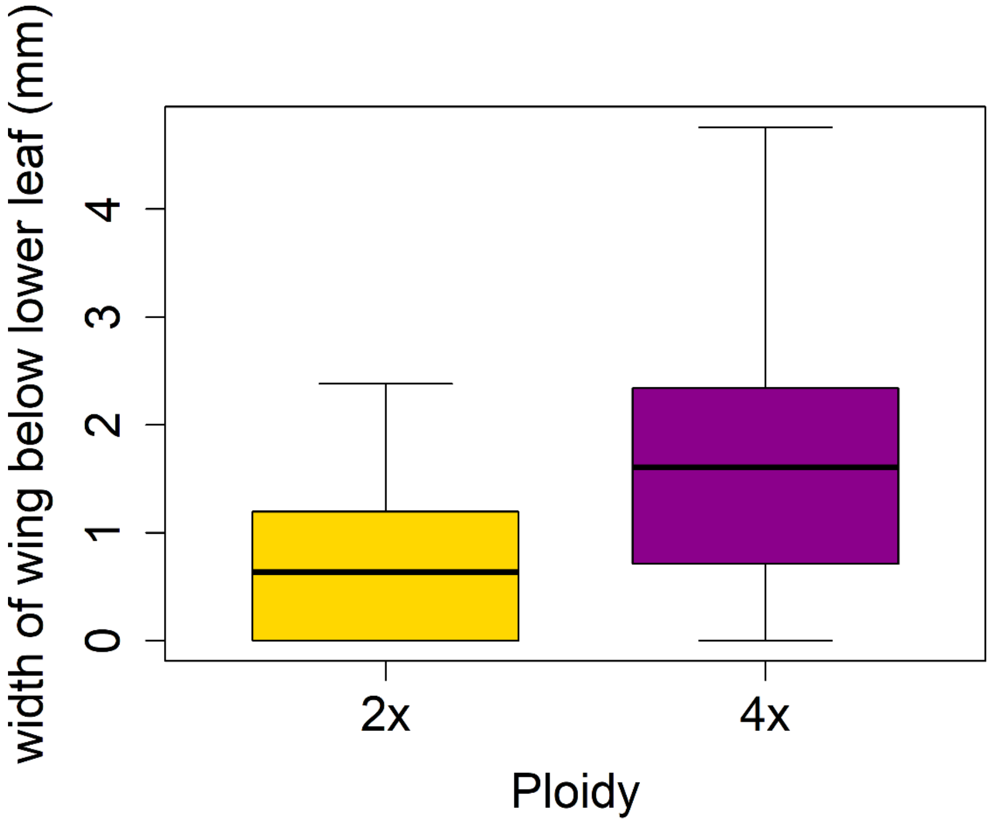
width of wing below lower leaf (mm)
0
1
2
3
4
2x
4x
Ploidy

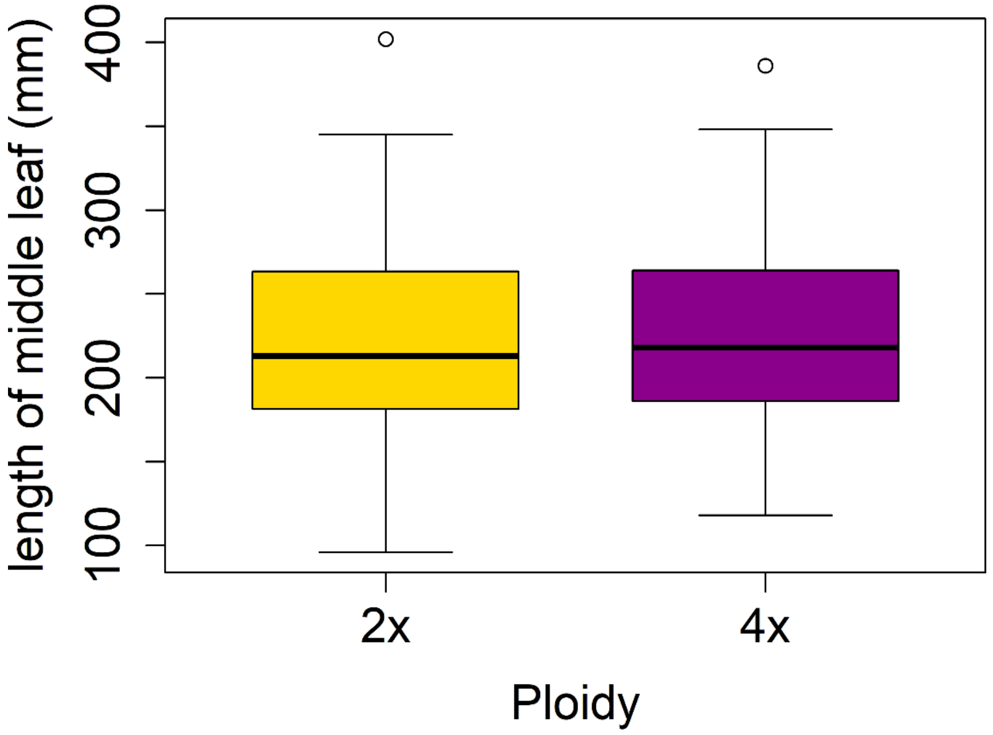
length of middle leaf (mm)
100
200
300
400
2x
4x
Ploidy

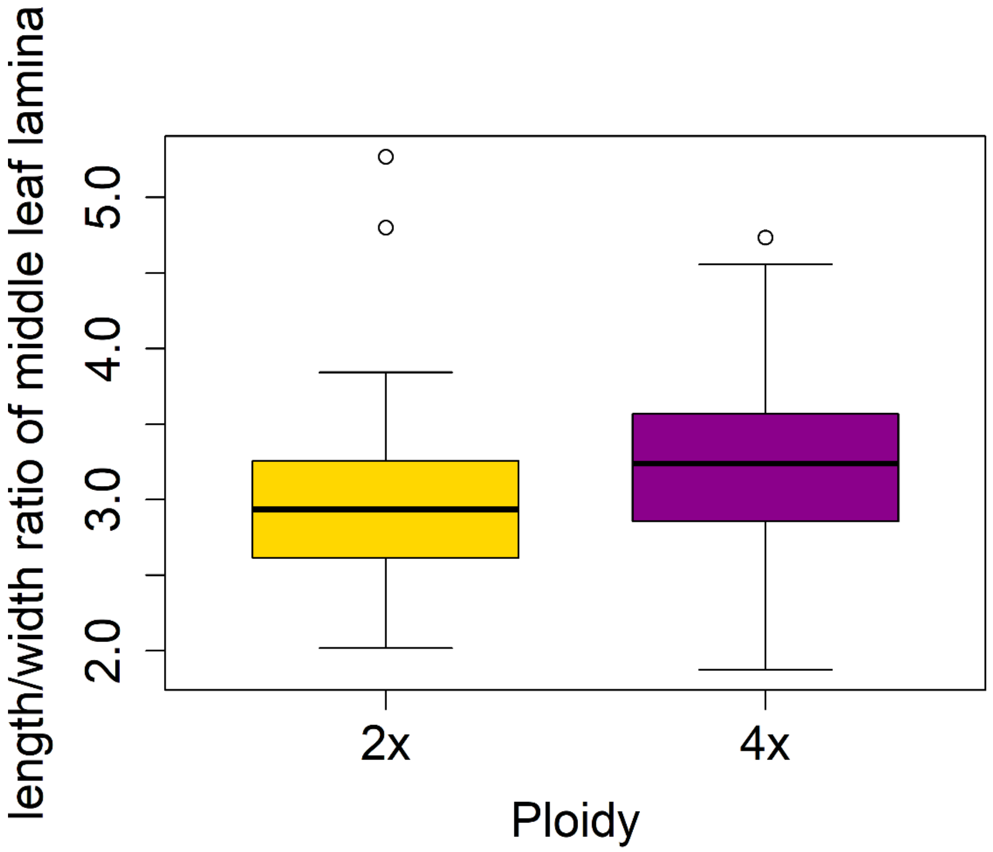
length/width ratio of middle leaf lamina
2.0
3.0
4.0
5.0
2x
4x
Ploidy

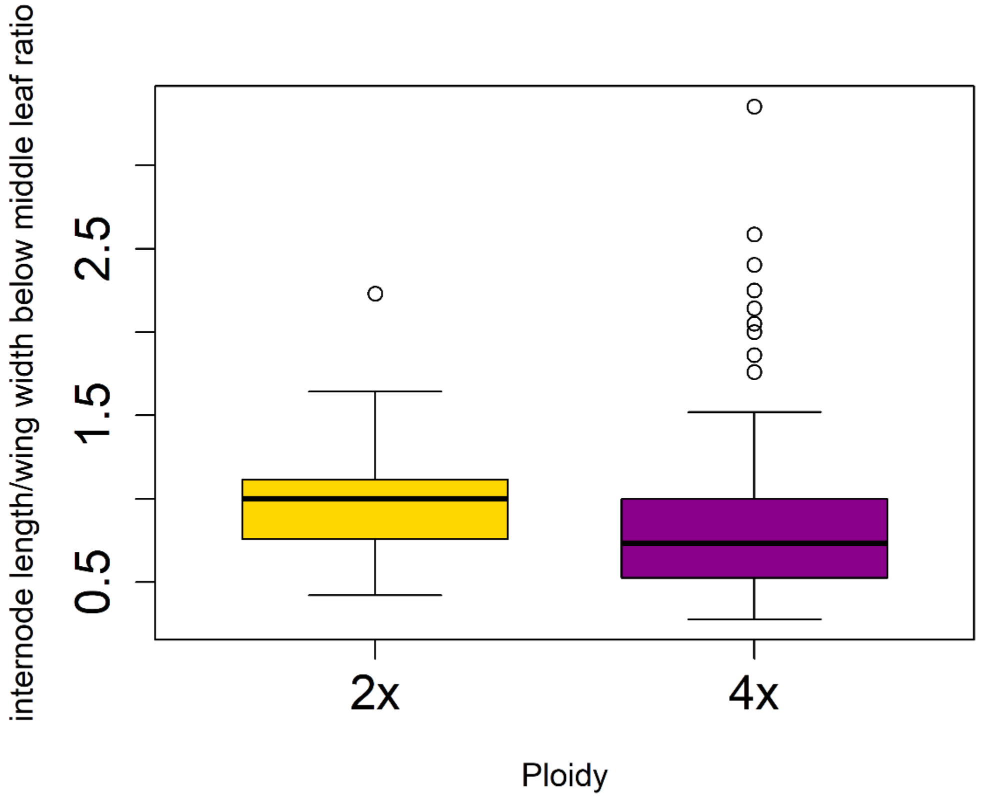
internode length/wing width below middle leaf ratio
0.5
1.5
2.5
2x
4x
Ploidy

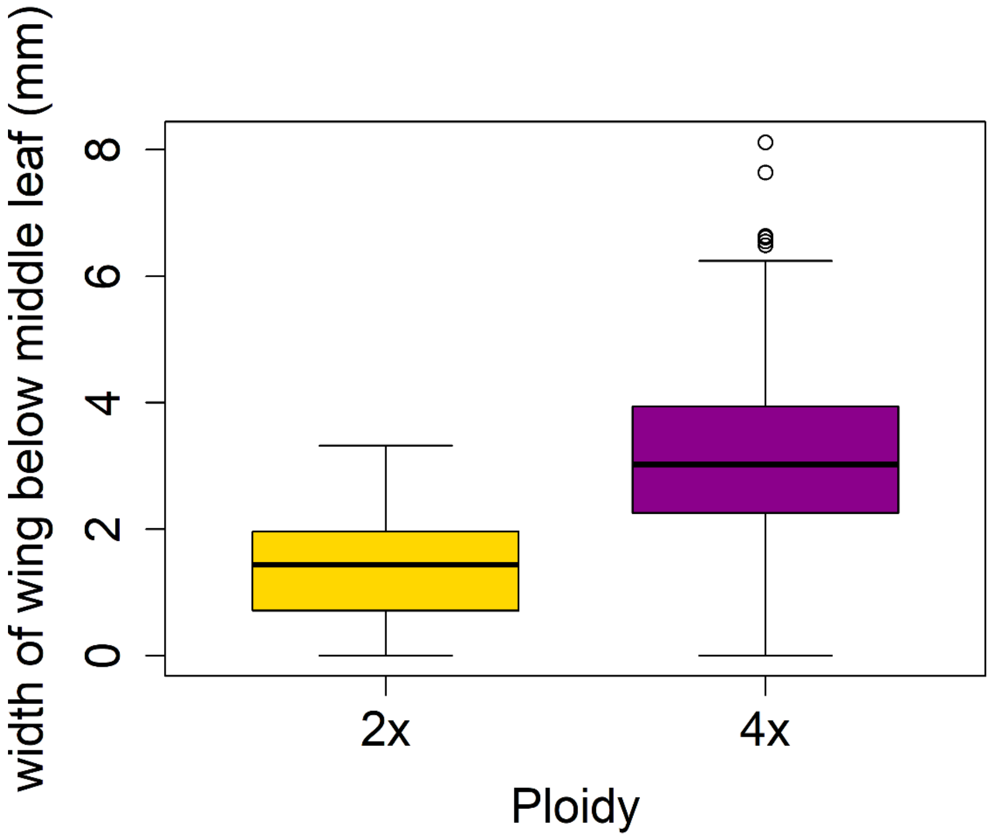

width of wing below middle leaf (mm)
0
2
4
6
8
2x
4x
Ploidy

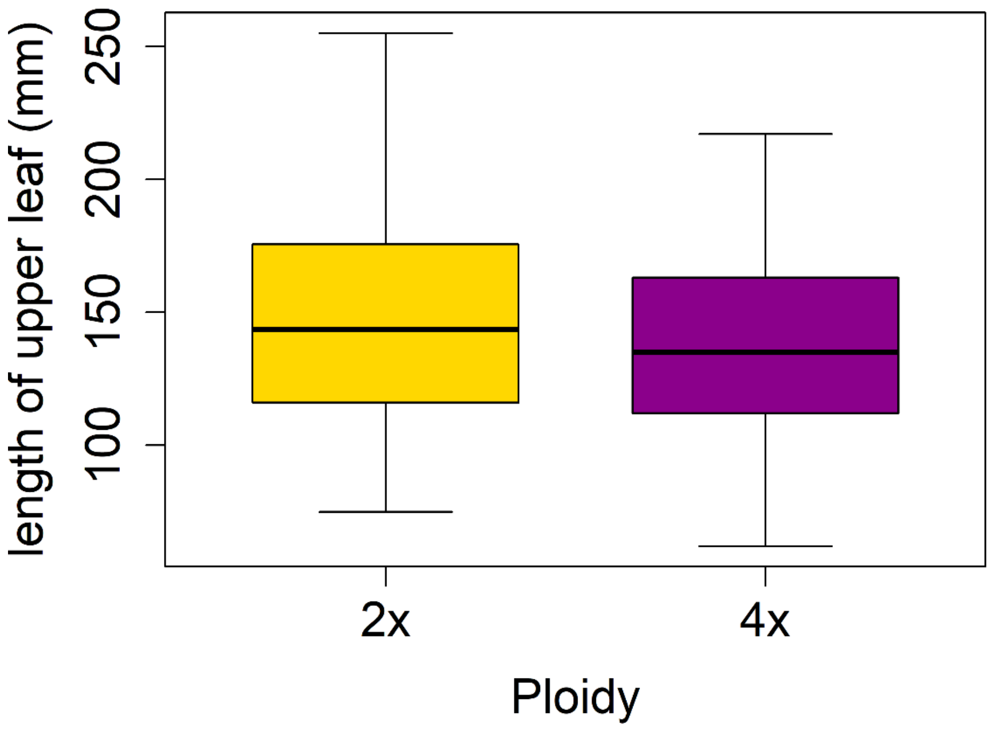

length of upper leaf (mm)
100
150
200
250
2x
4x
Ploidy

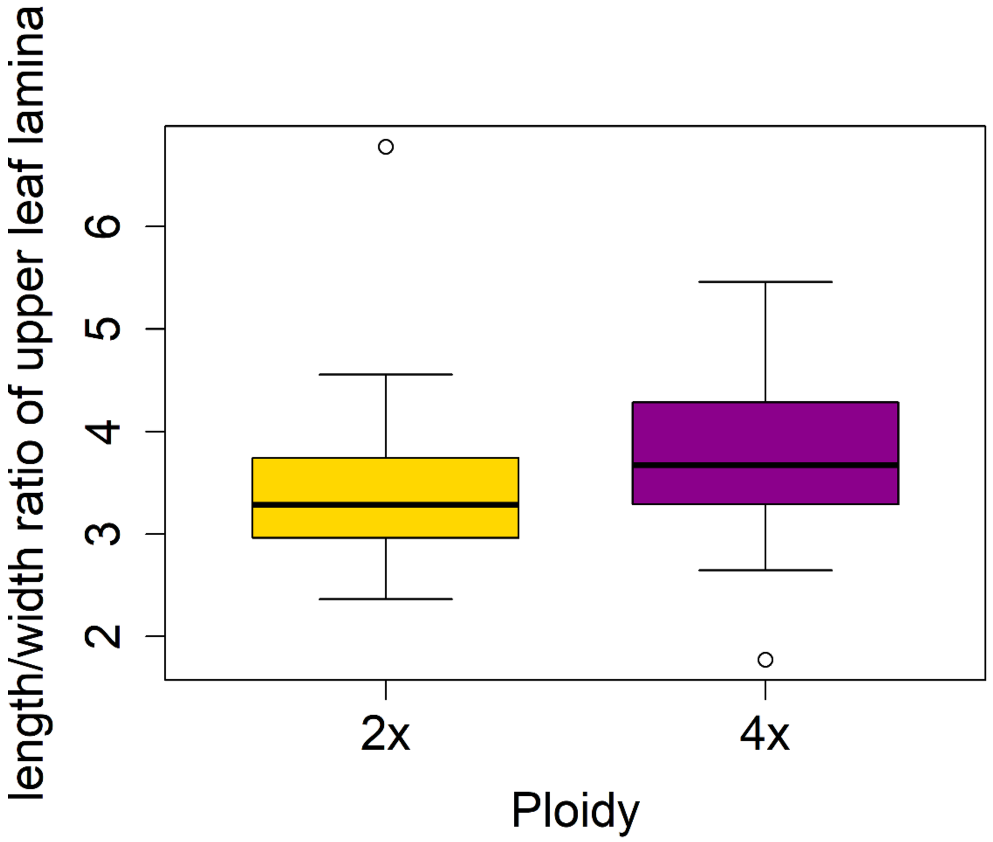
length/width ratio of upper leaf lamina
2
3
4
5
6
2x
4x
Ploidy

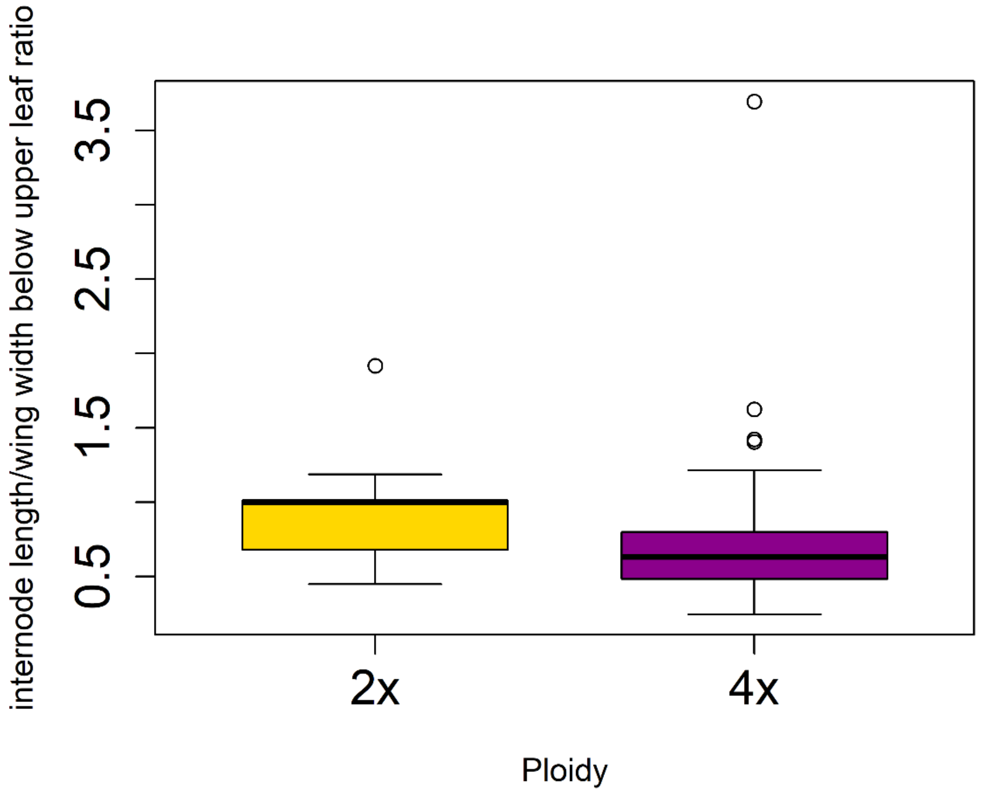
internode length/wing width below upper leaf ratio
0.5
1.5
2.5
3.5
2x
4x
Ploidy

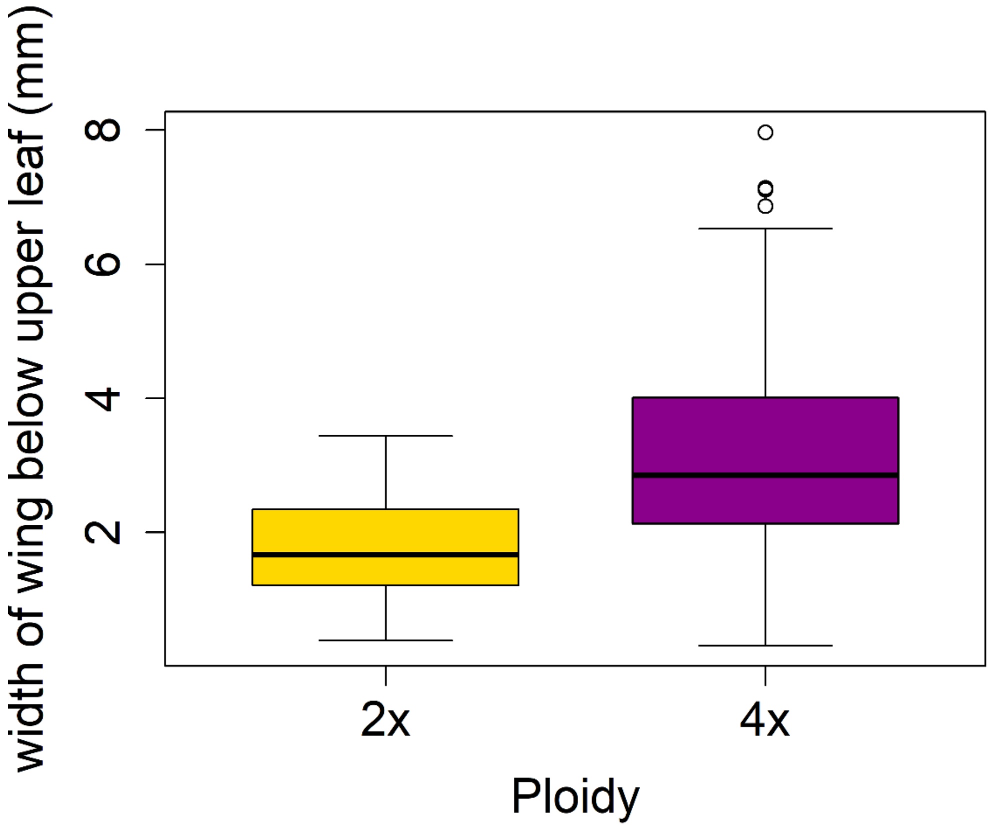

width of wing below upper leaf (mm)
2
4
6
8
2x
4x
Ploidy

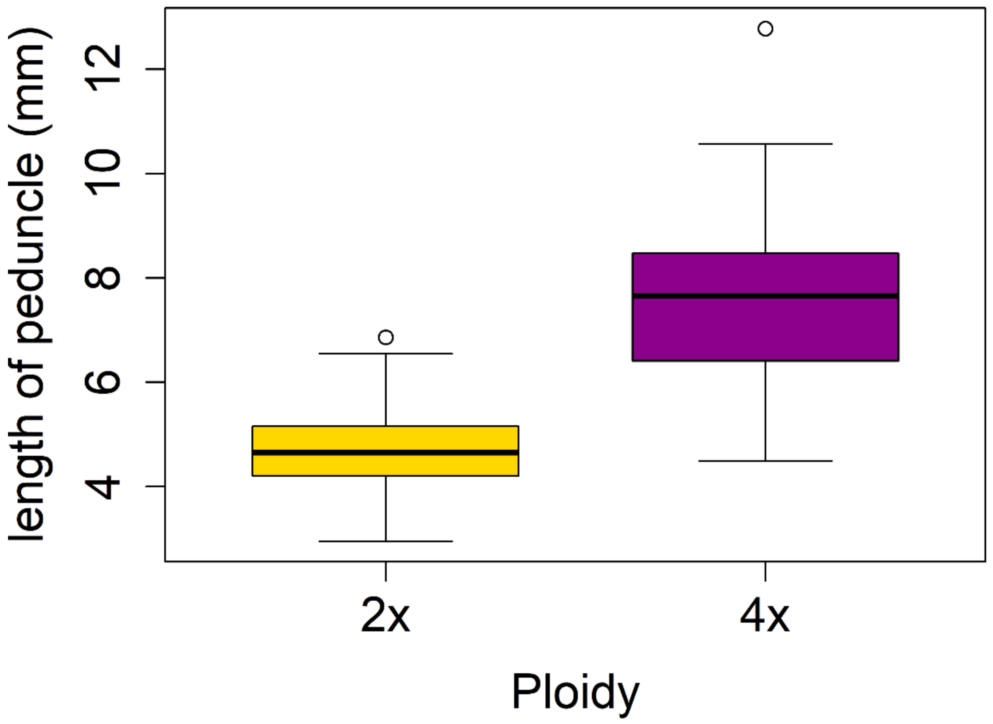

length of peduncle (mm)
4
6
8
10
12
2x
4x
Ploidy

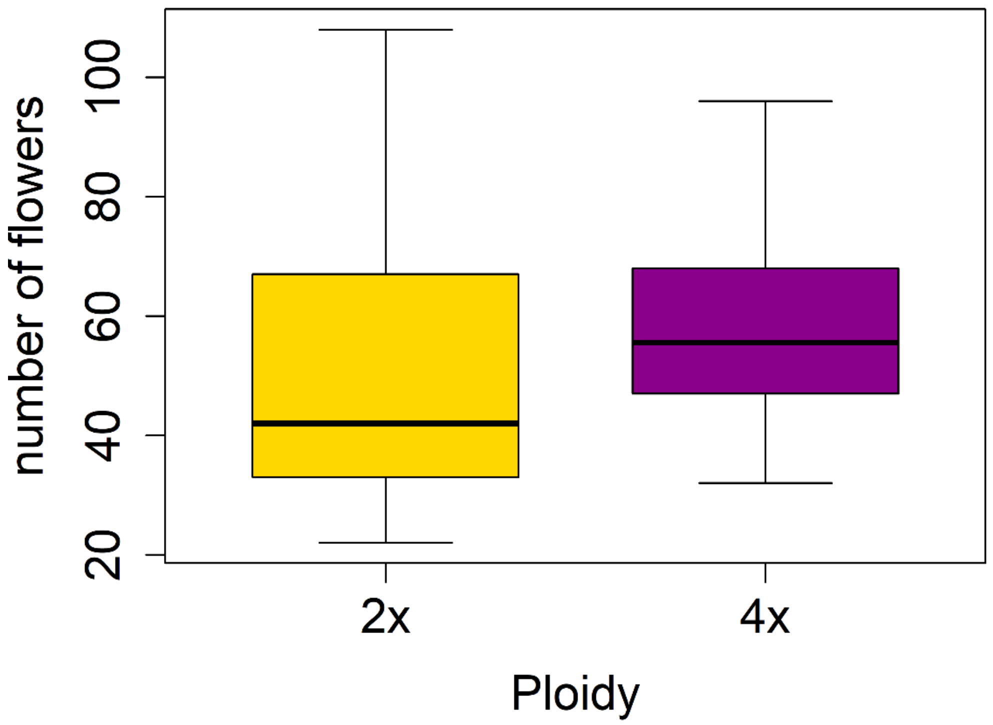

number of flowers
20
40
60
80
100
2x
4x
Ploidy

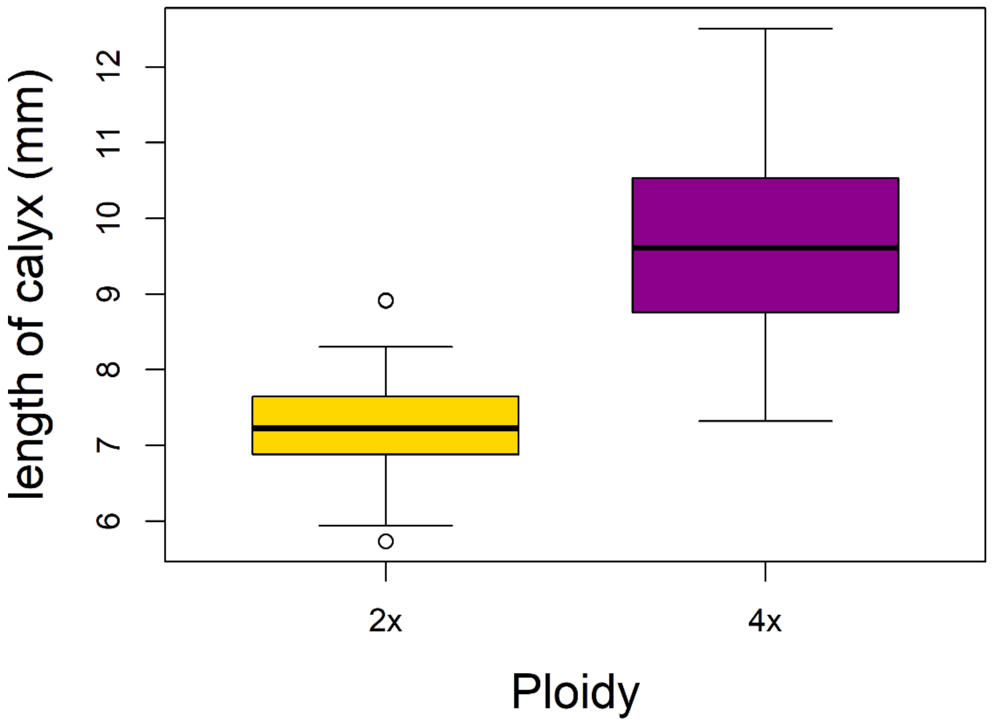

length of calyx (mm)
6
7
8
9
10
11
12
2x
4x
Ploidy

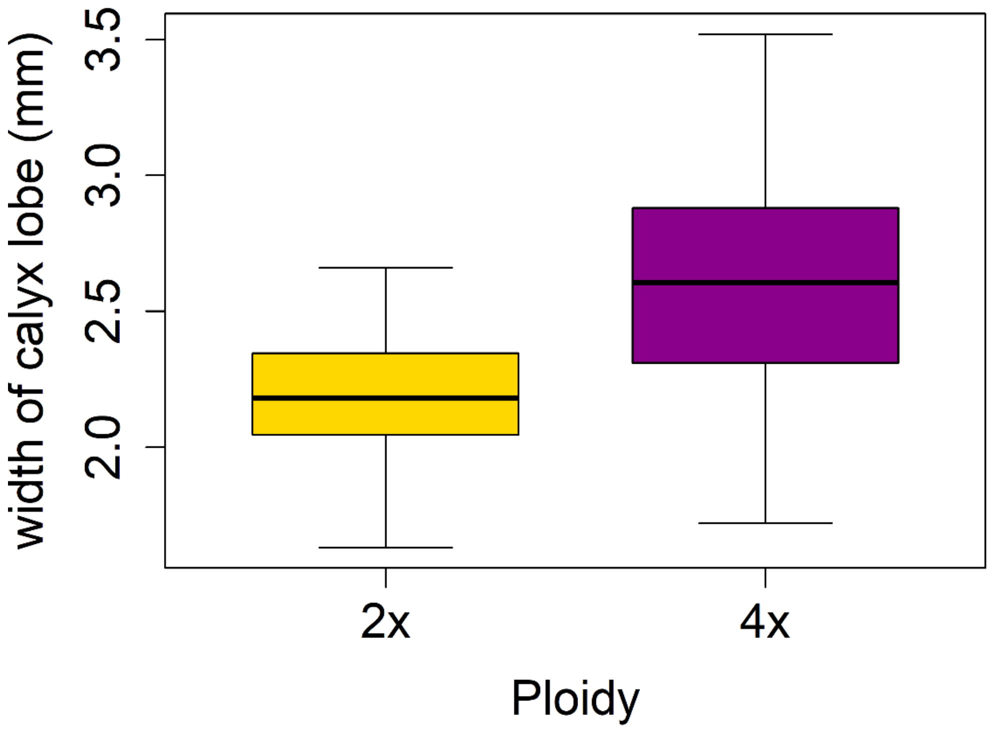
width of calyx lobe (mm)
3.5
3.0
2.5
2.0
2x
4x
Ploidy

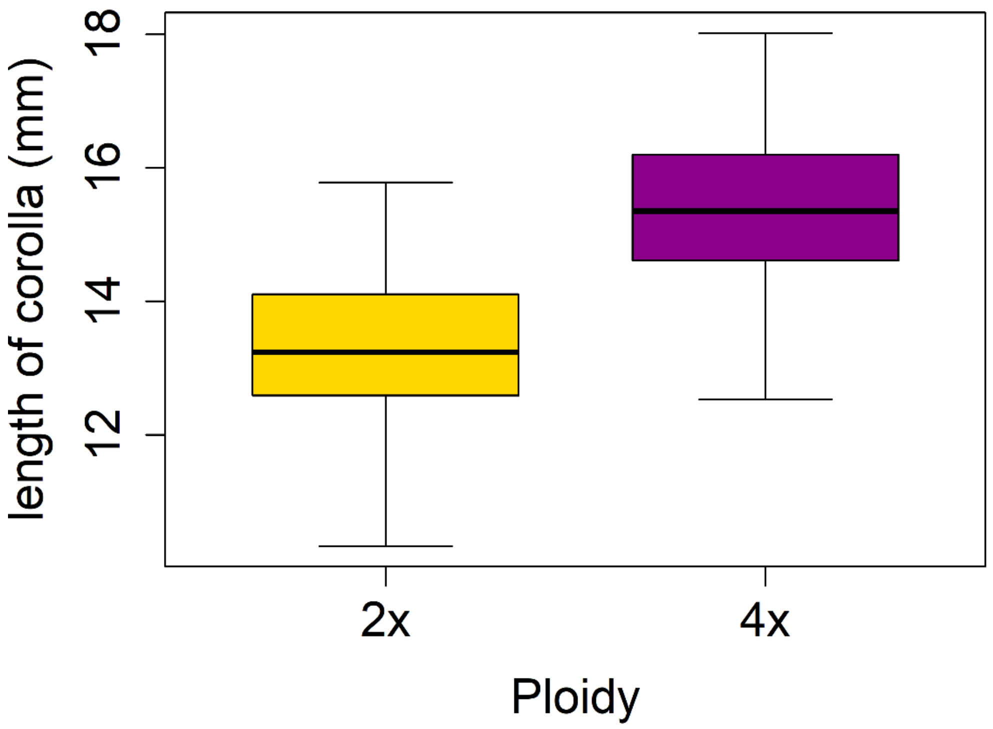
length of corolla (mm)
18
16
14
12
2x
4x
Ploidy

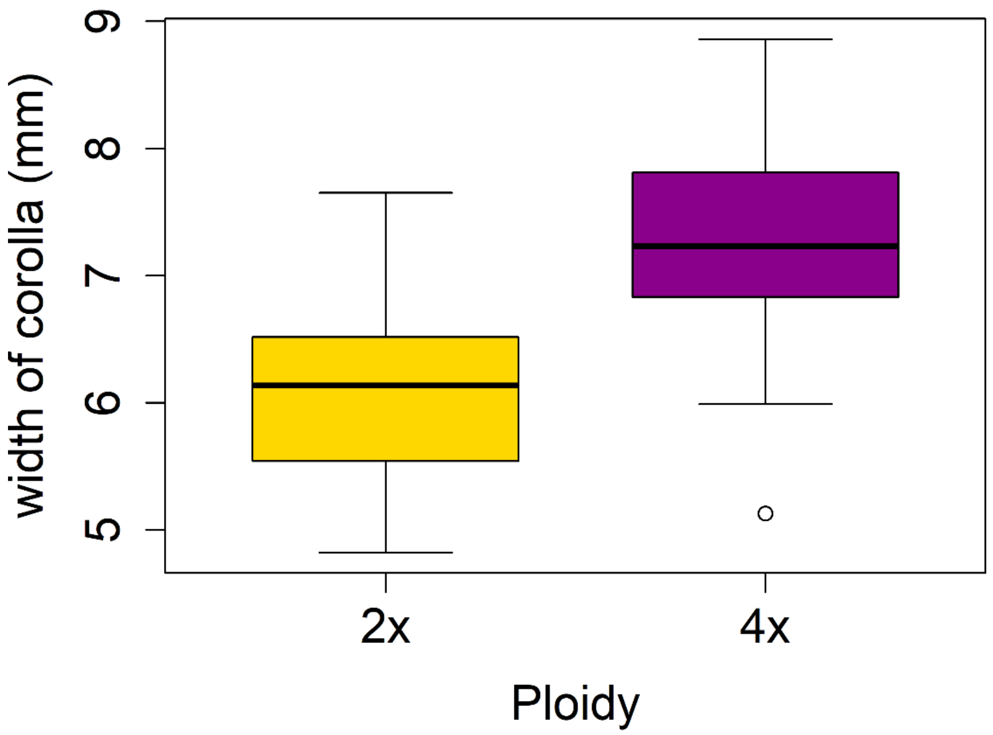

9
8
7
6
5
width of corolla (mm)
2x
4x
Ploidy

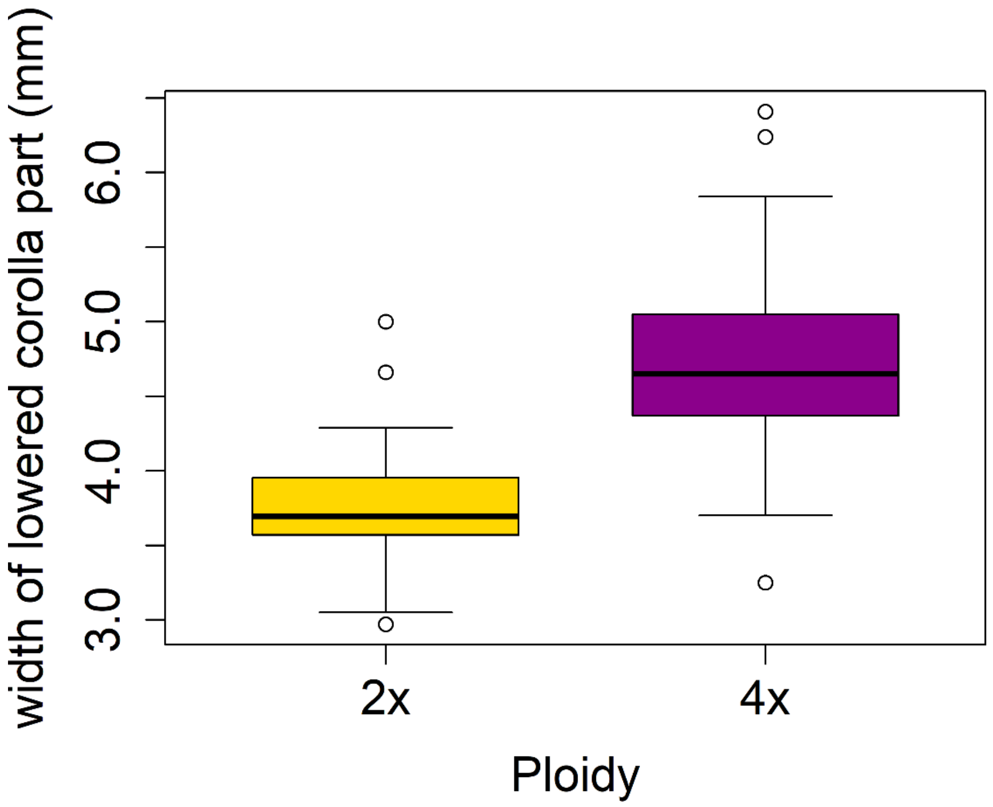

6.0
5.0
4.0
3.0
width of lowered corolla part (mm)
2x
4x
Ploidy

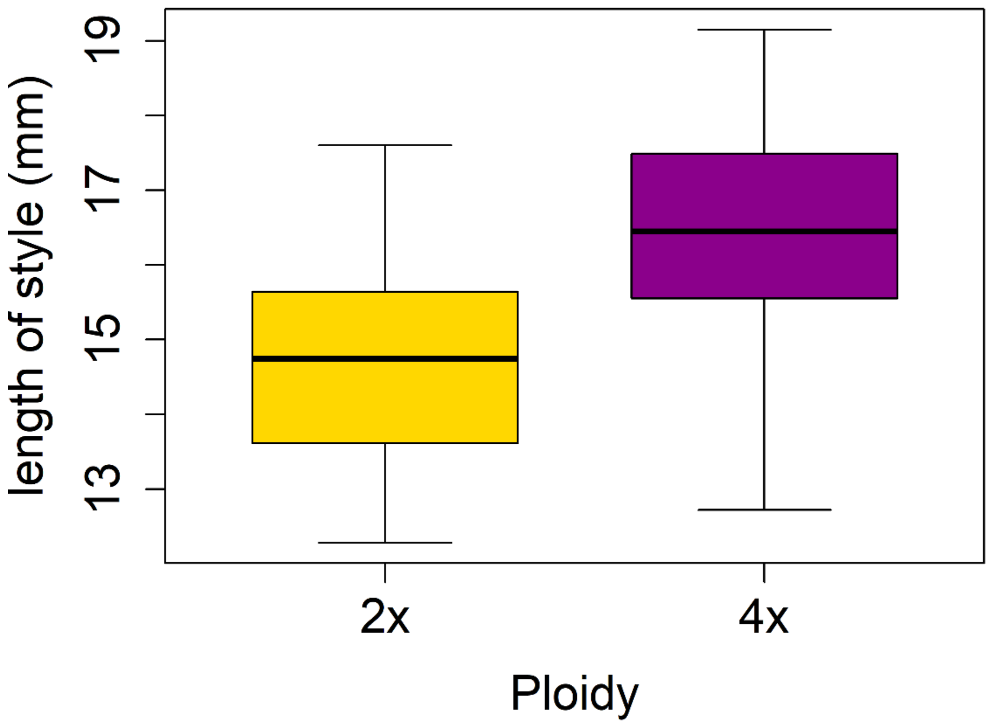

length of style (mm)
13
15
17
19
2x
4x
Ploidy

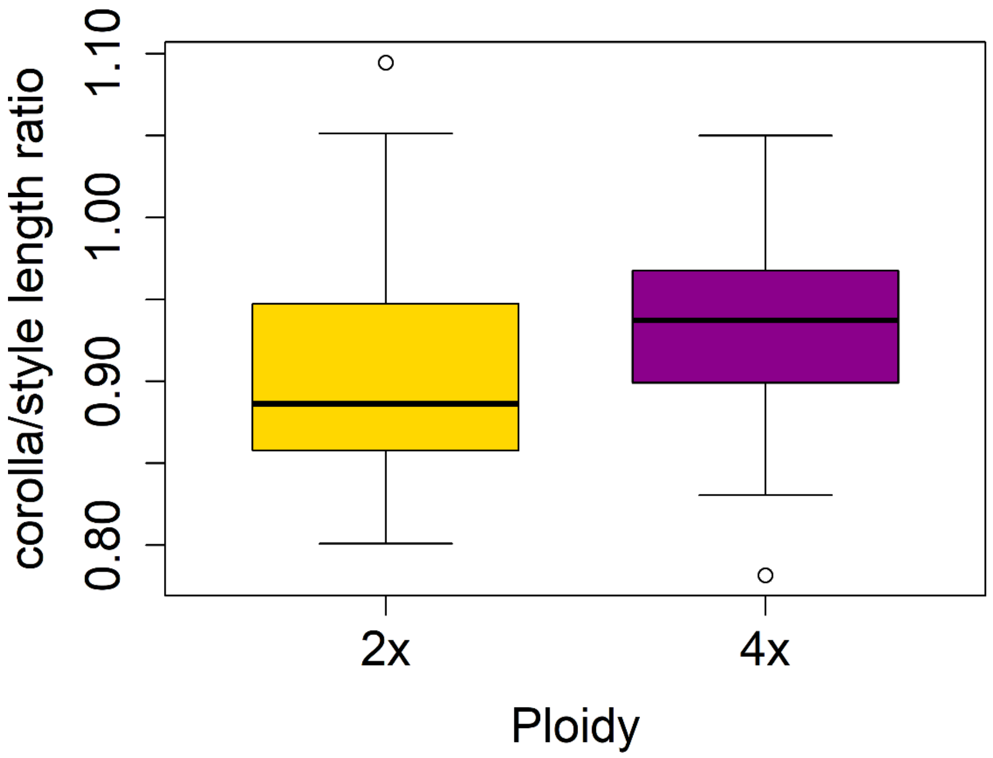

corolla/style length ratio
0.80
0.90
1.00
1.10
2x
4x
Ploidy

Table S8. Total canonical structure expressing correlations of traits with canonical axes in discriminate analysis (CDA). Values that exceed the level of 0.2 are in bold.

Character	CCA1
height of stem	0.050031
width of stem	-0.19564
width of stem cavity	-0.02289
width of stem wall	**-0.24566**
stem/cavity width ratio	0.037163
colour of plant	**0.353127**
number of branches	-0.19482
length of rosette leaf	-0.07854
length/width ratio of rosette lamina	0.127397
width of lower petiole wing	-0.13158
length of lower leaf	0.056914
length/width ratio of lower leaf lamina	-0.00198
internode length/wing width below lower leaf ratio	0.01215
width of wing below lower leaf	-0.19421
length of middle leaf	0.006088
length/width ratio of middle leaf lamina	-0.10595
internode length/wing width below middle leaf ratio	0.069246
width of wing below middle leaf	**-0.31454**
length of upper leaf	0.056715
length/width ratio of upper leaf lamina	-0.13609
internode length/wing width below upper leaf ratio	0.147539
width of wing below upper leaf	**-0.25331**
number of flowers	-0.09825
length of peduncle	**-0.51534**
length of calyx	**-0.57035**
width of calyx lobe	**-0.30067**
length of corolla	**-0.43128**
width of corolla	**-0.40847**
width of lowered corolla part	**-0.46736**
length of style	**-0.32808**
corolla/style length ratio	-0.12167
shape of style	0.177798
