# Supplementary material for: Morphological, ecological and geographic differences between diploids and tetraploids of Symphytum officinale (Boraginaceae) justify both cytotypes as separate species
Source: AoB Plants. 2022 Jun 21;14(4):plac028. doi: 10.1093/aobpla/plac028 (PMC9297162; doi:10.1093/aobpla/plac028)

**Supporting Information**

**Article title:** Morphological, ecological and geographic differences between diploids and tetraploids of *Symphytum officinale* (Boraginaceae) justify both cytotypes as separate species

**Authors:** Lucie Kobrlová, Martin Duchoslav & Michal Hroneš

**Figure S1.** Response curves that show how each environmental variable affects the Maxent prediction.

Below response curves of selected environmental variables for diploids (2*x*) and tetraploids (4*x*) of *Symphytum officinale* s.l. are reported. The curves show the mean response of the 10 replicate Maxent runs (red) and ± 1 SD (blue).

Environmental variable (abbreviation, x-label in the graphs)

Mean annual solar radiation (SRAD; SRAD)

Annual Mean Temperature (bio1; wcbio1)

Max Temperature of Warmest Month (bio5; wcbio5)

Temperature Annual Range (bio7; wcbio7)

Precipitation Seasonality (bio15; wcbio15)

Volumetric percentage of coarse fragments (>2 mm) (crfvol; crfvol_sr2)


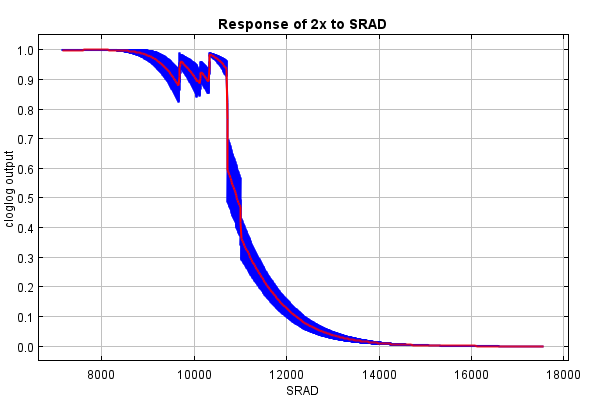


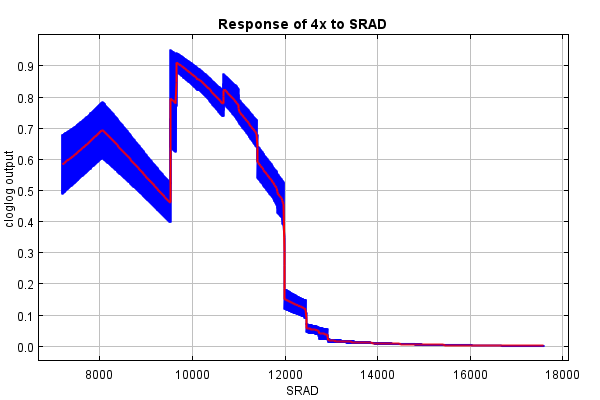


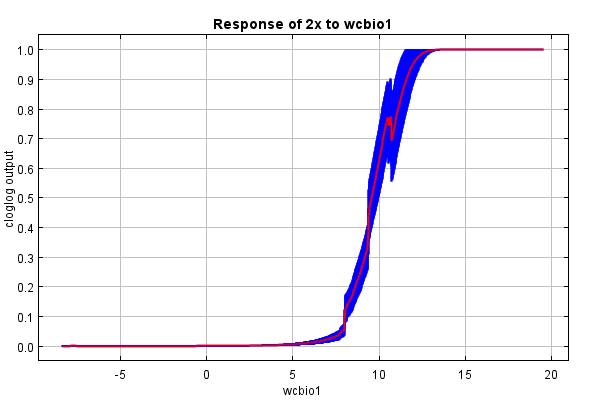


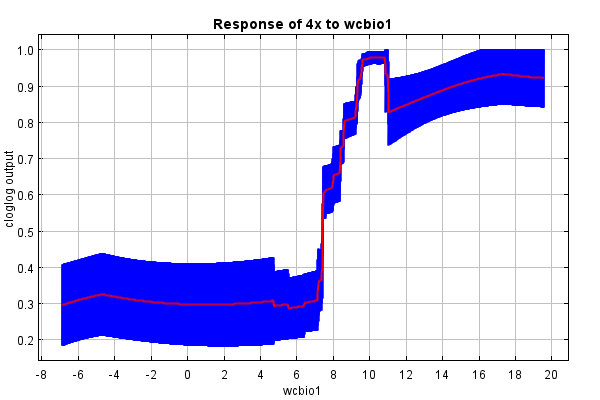


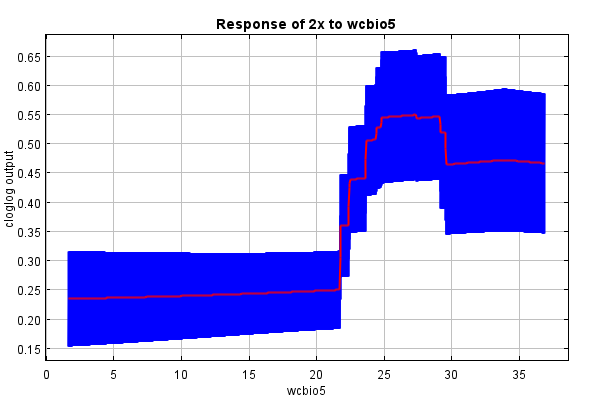


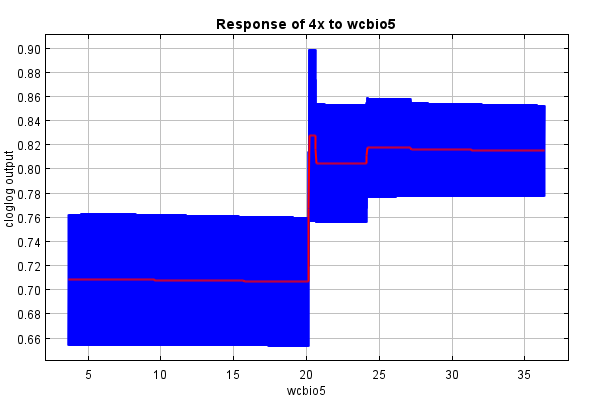


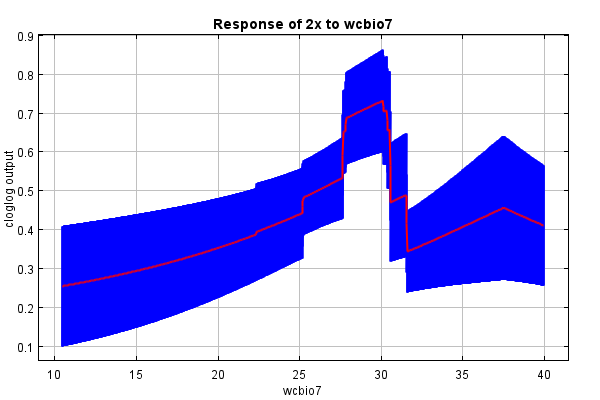

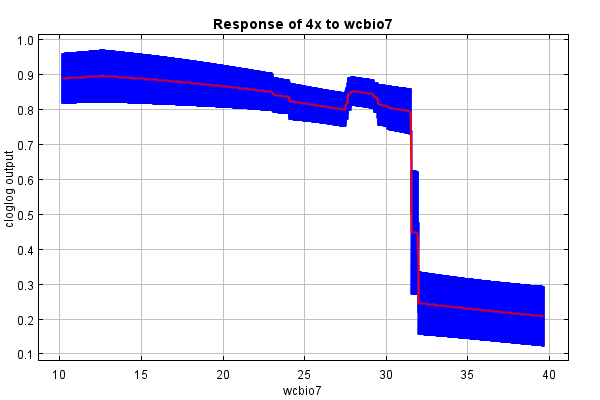


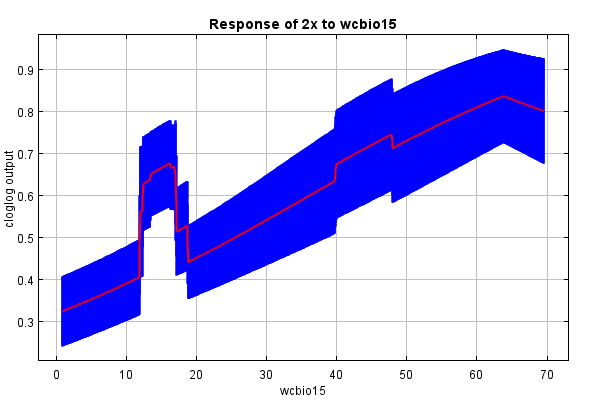


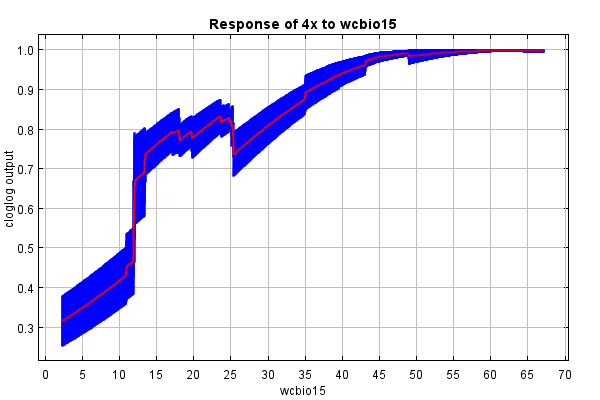


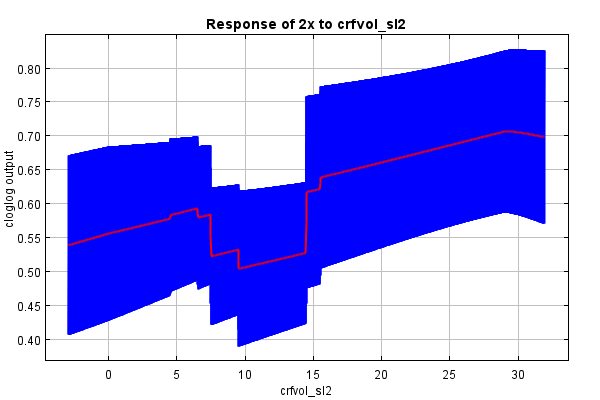


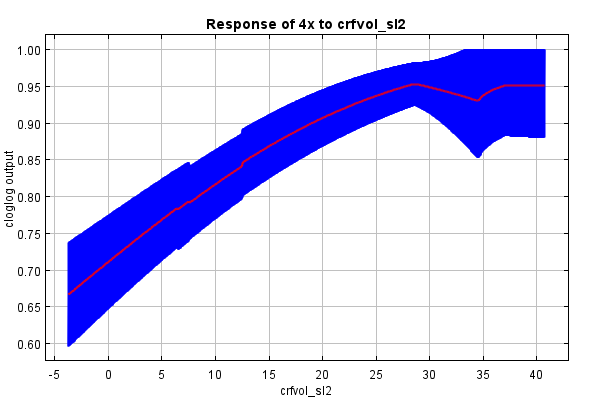


**Figure S2.** Variation of the mean site EIVs derived from phytocoenological relevés with results of one-way ANOVA with the modified permutation test with 499 permutations.


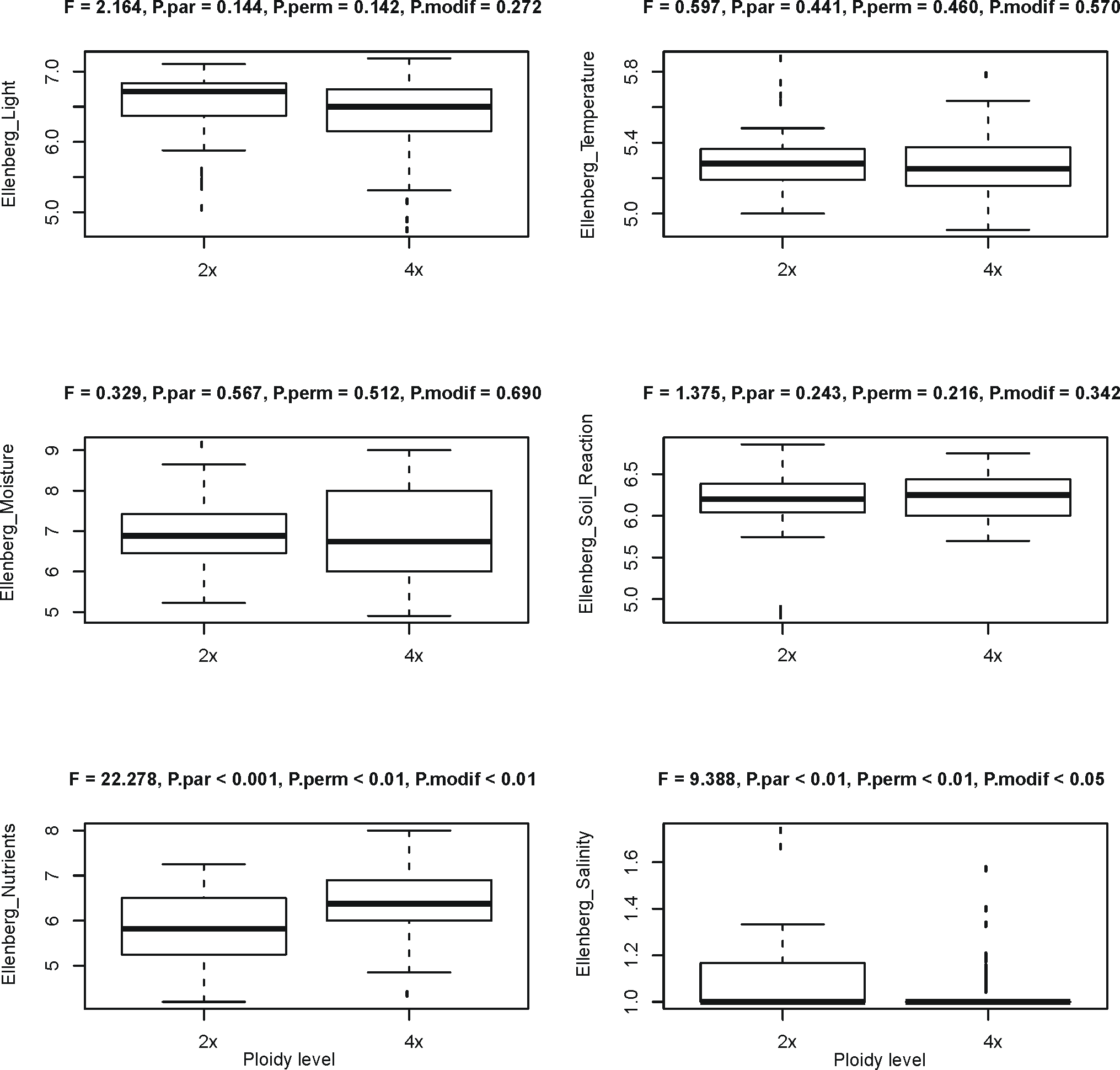


**Figure S3.** Box plots of quantitative morphological characters and their ratios. Box plot body define the 25th and 75th percentiles, horizontal lines show the median, whiskers are from the 10 to 90 percentiles, circles show extreme values).


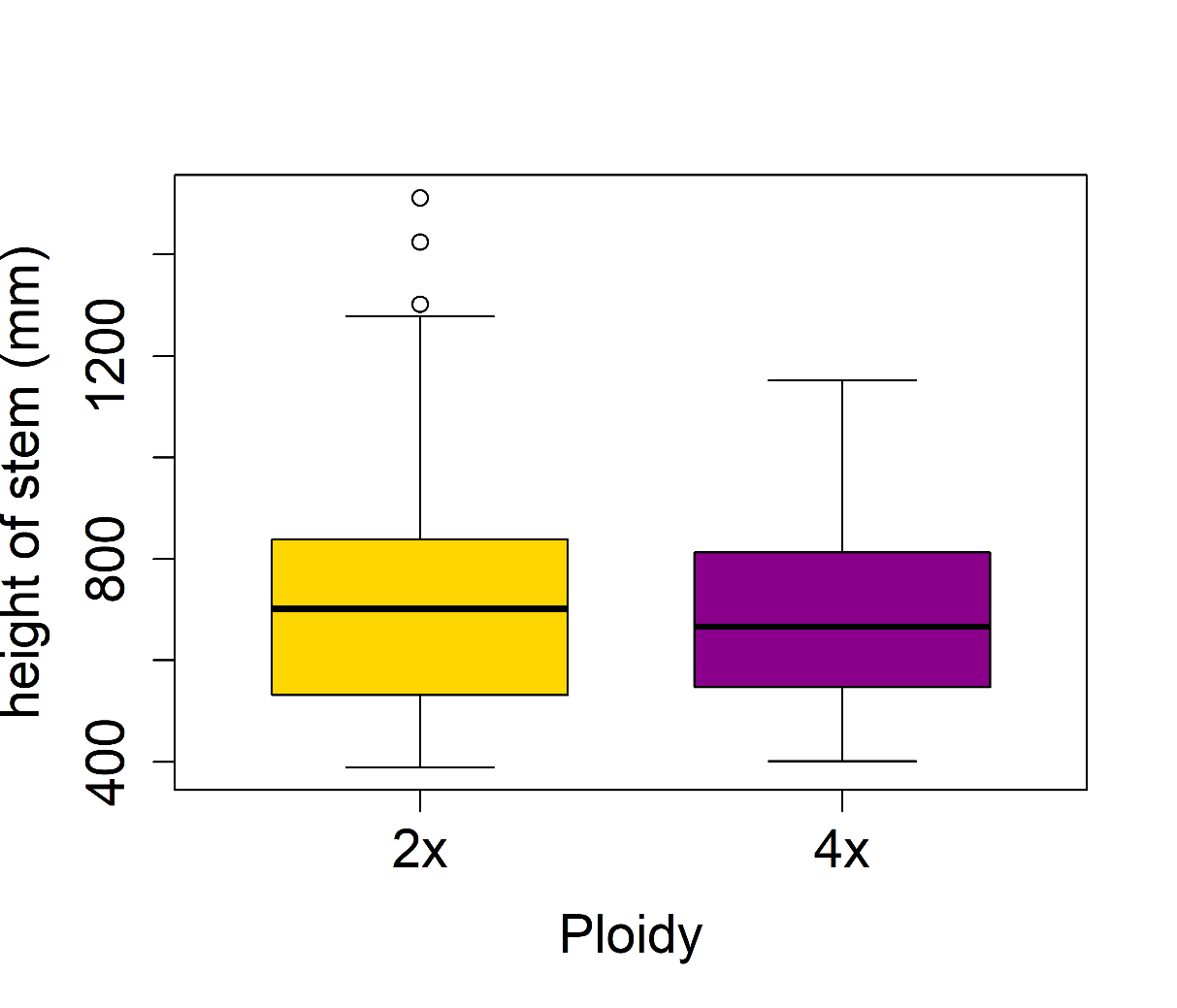


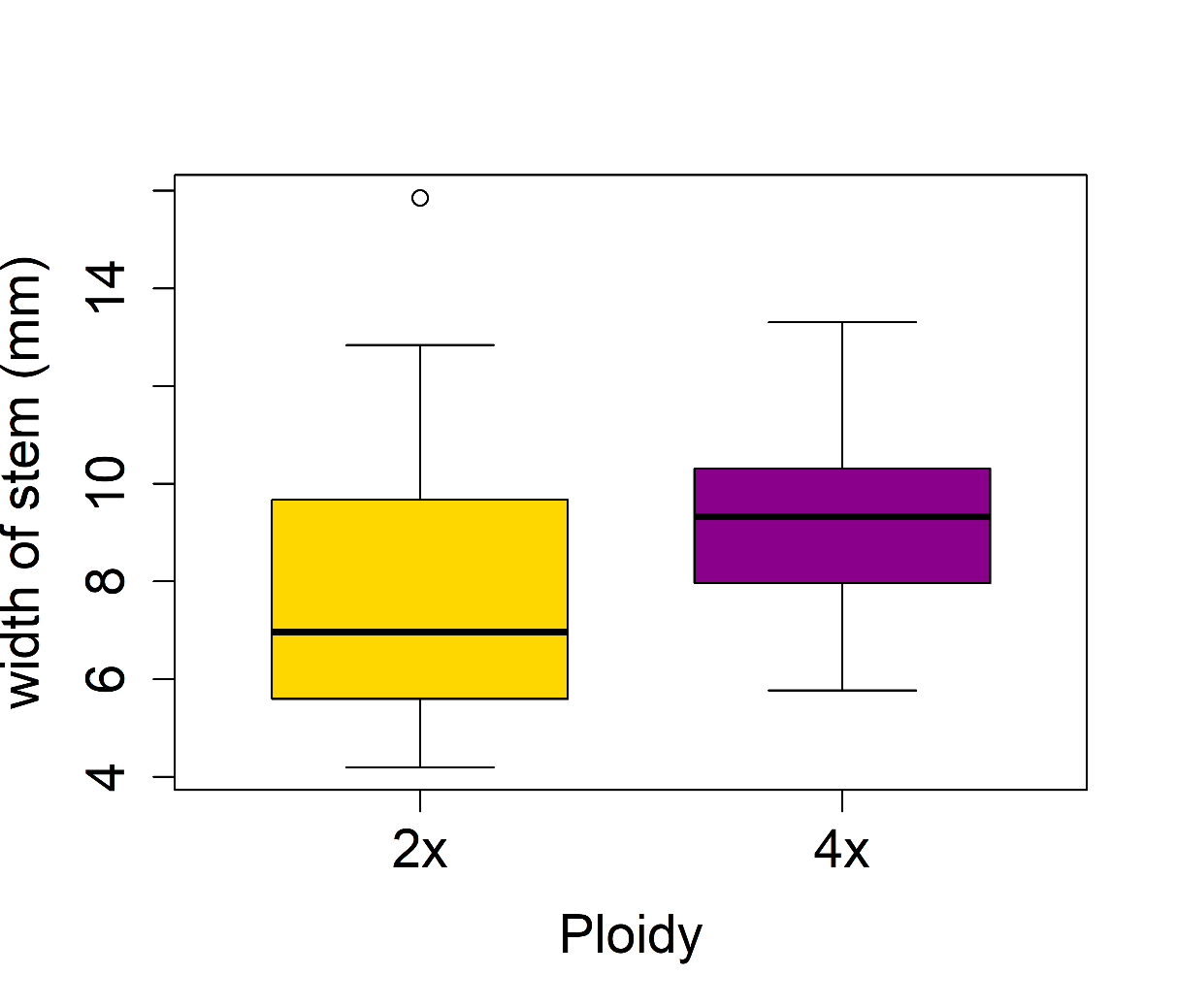


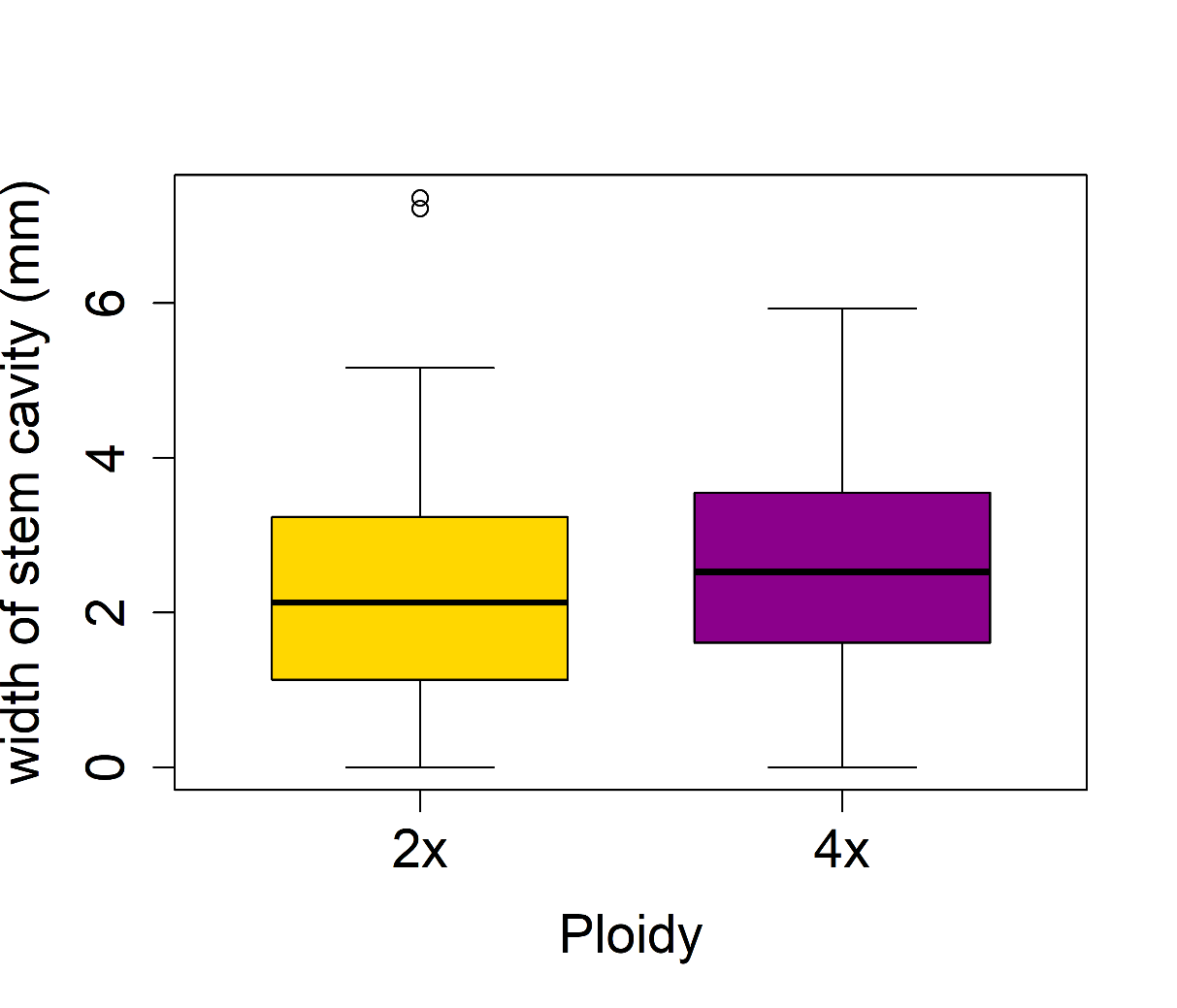

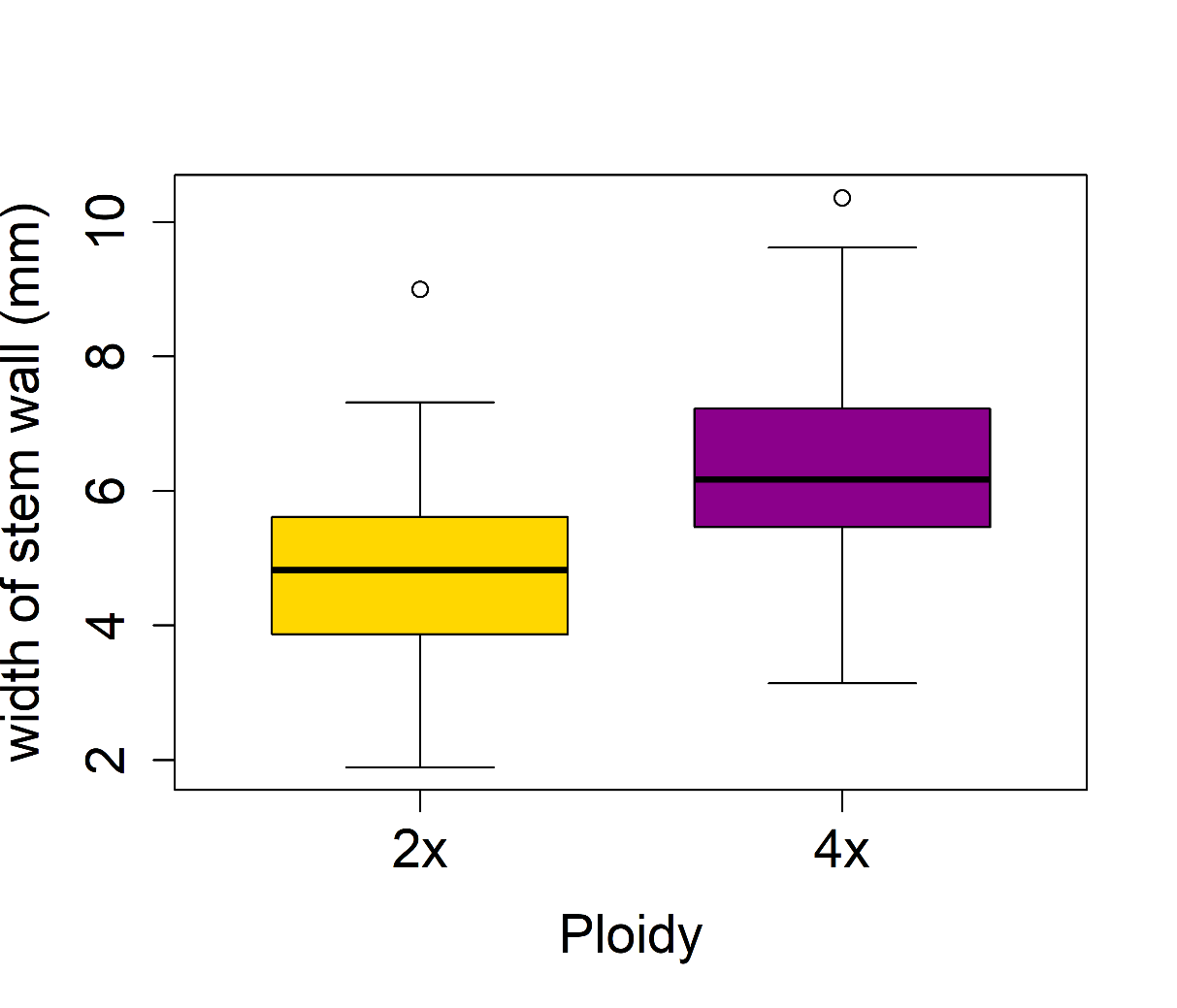

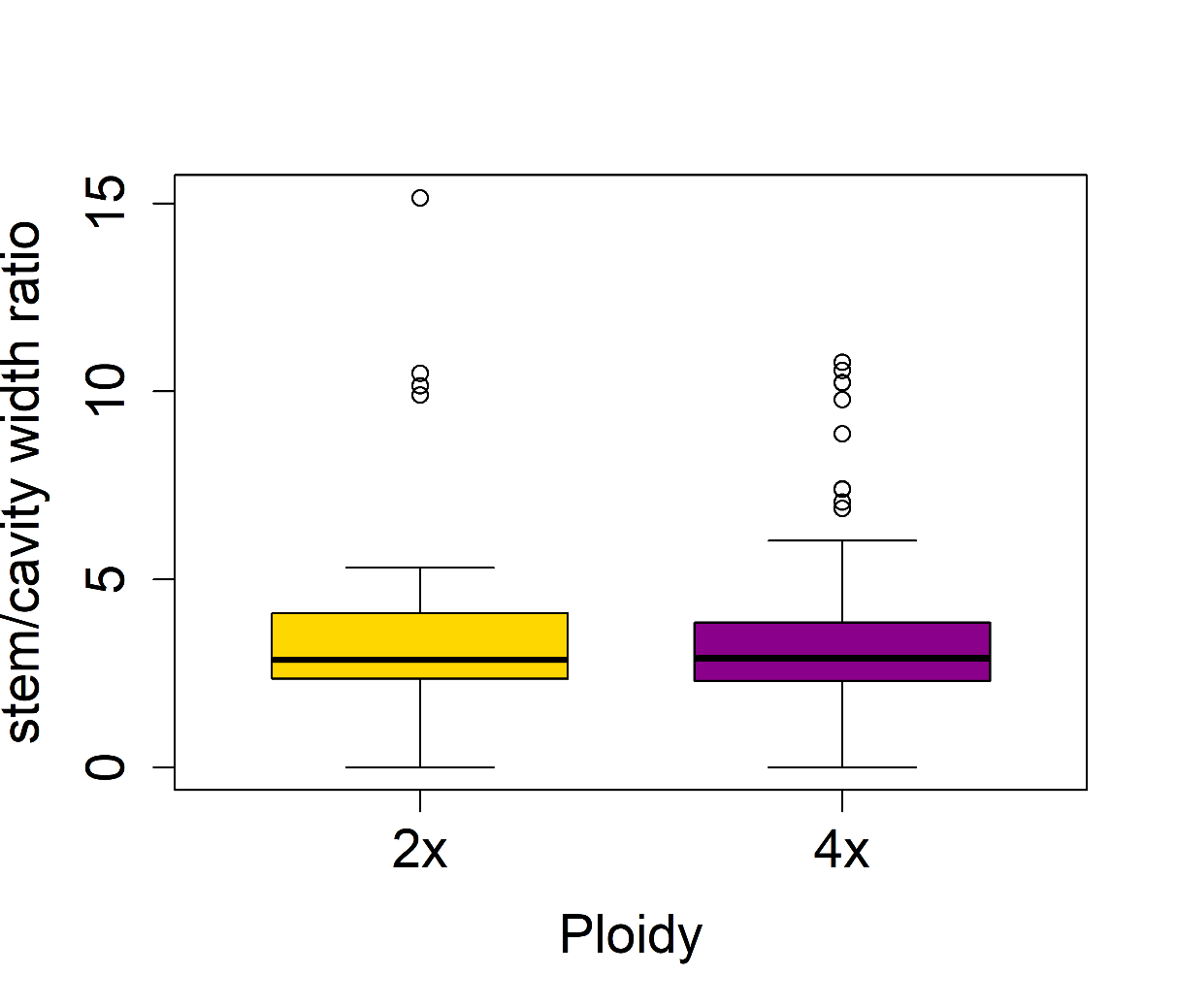

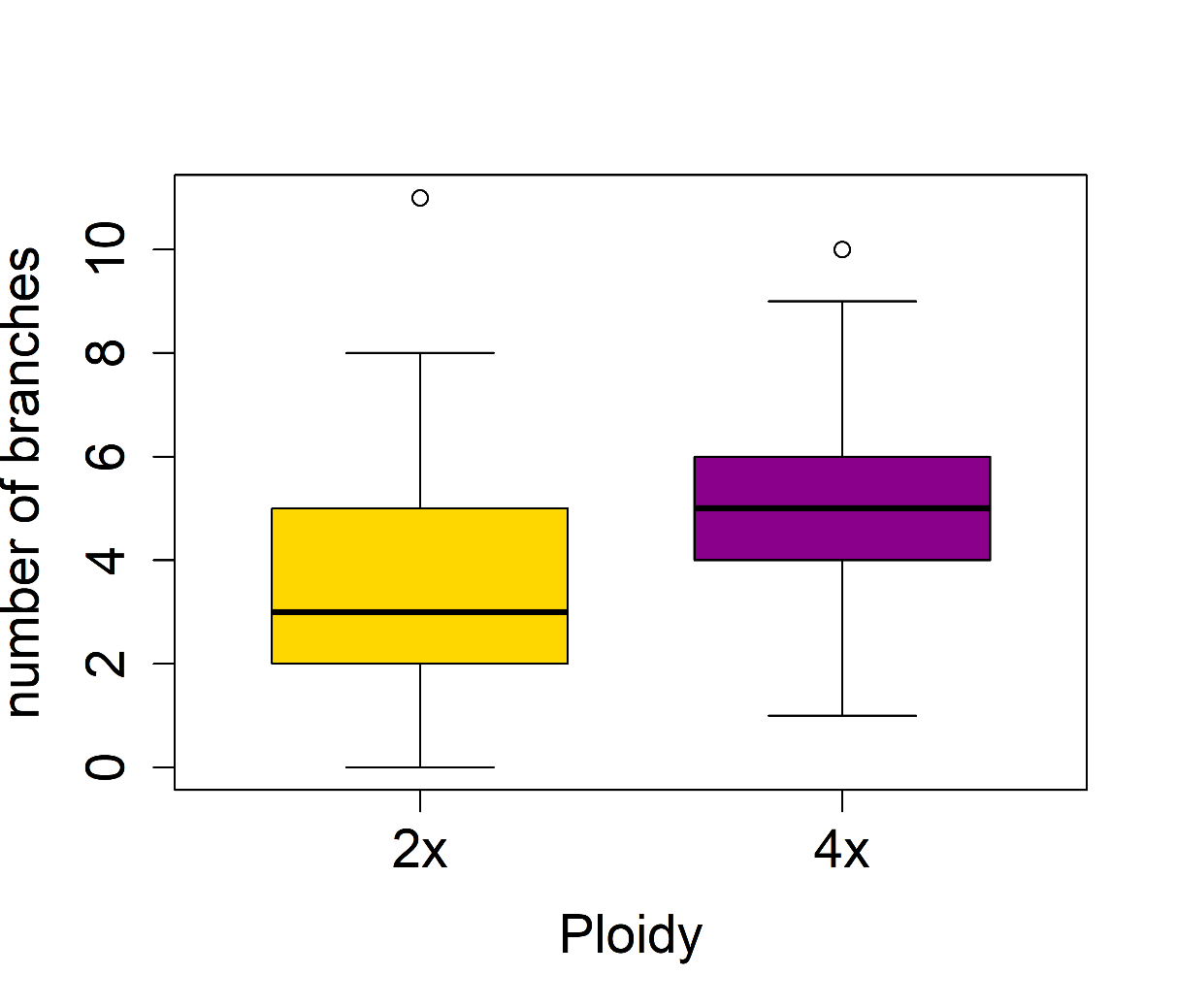

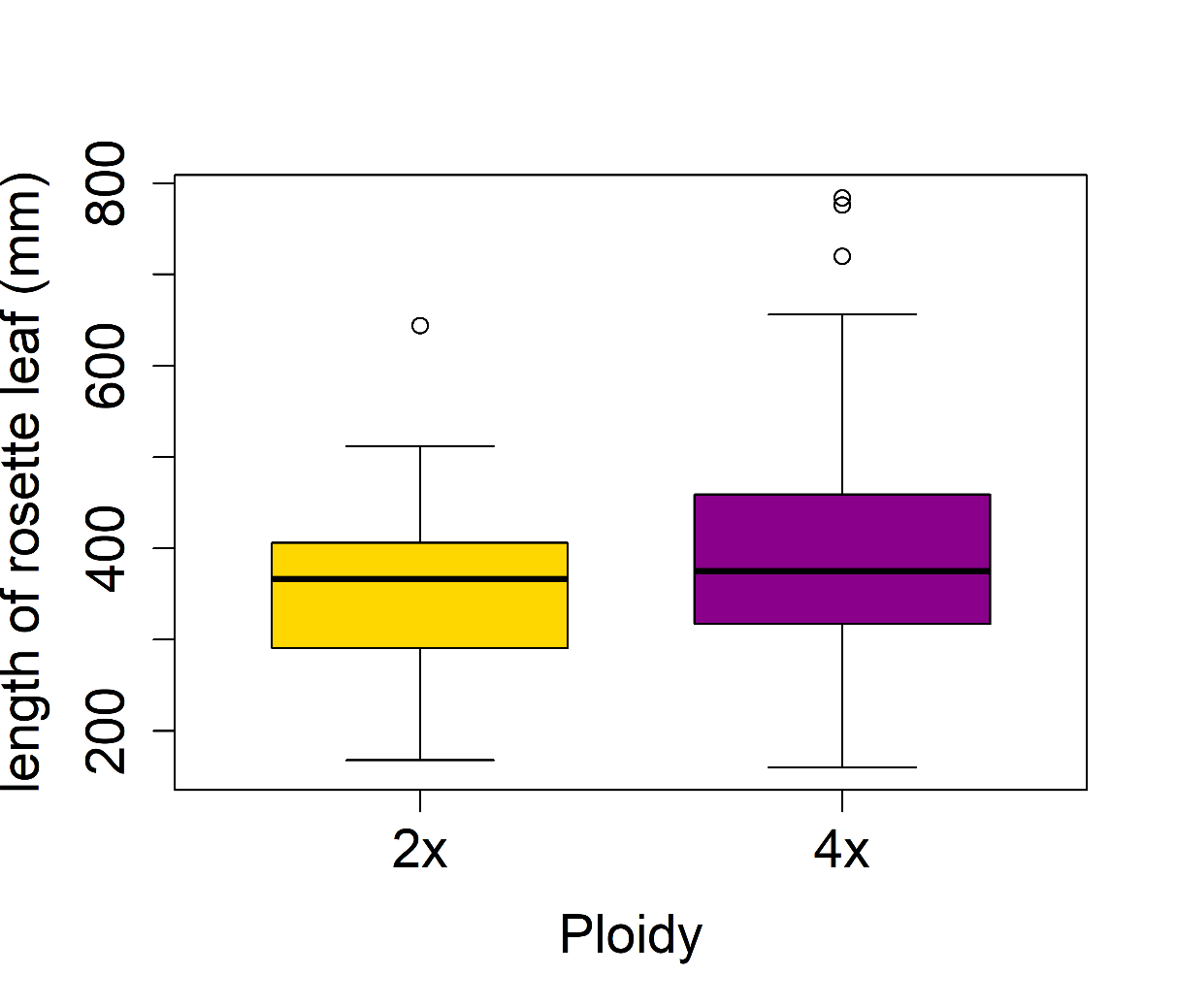

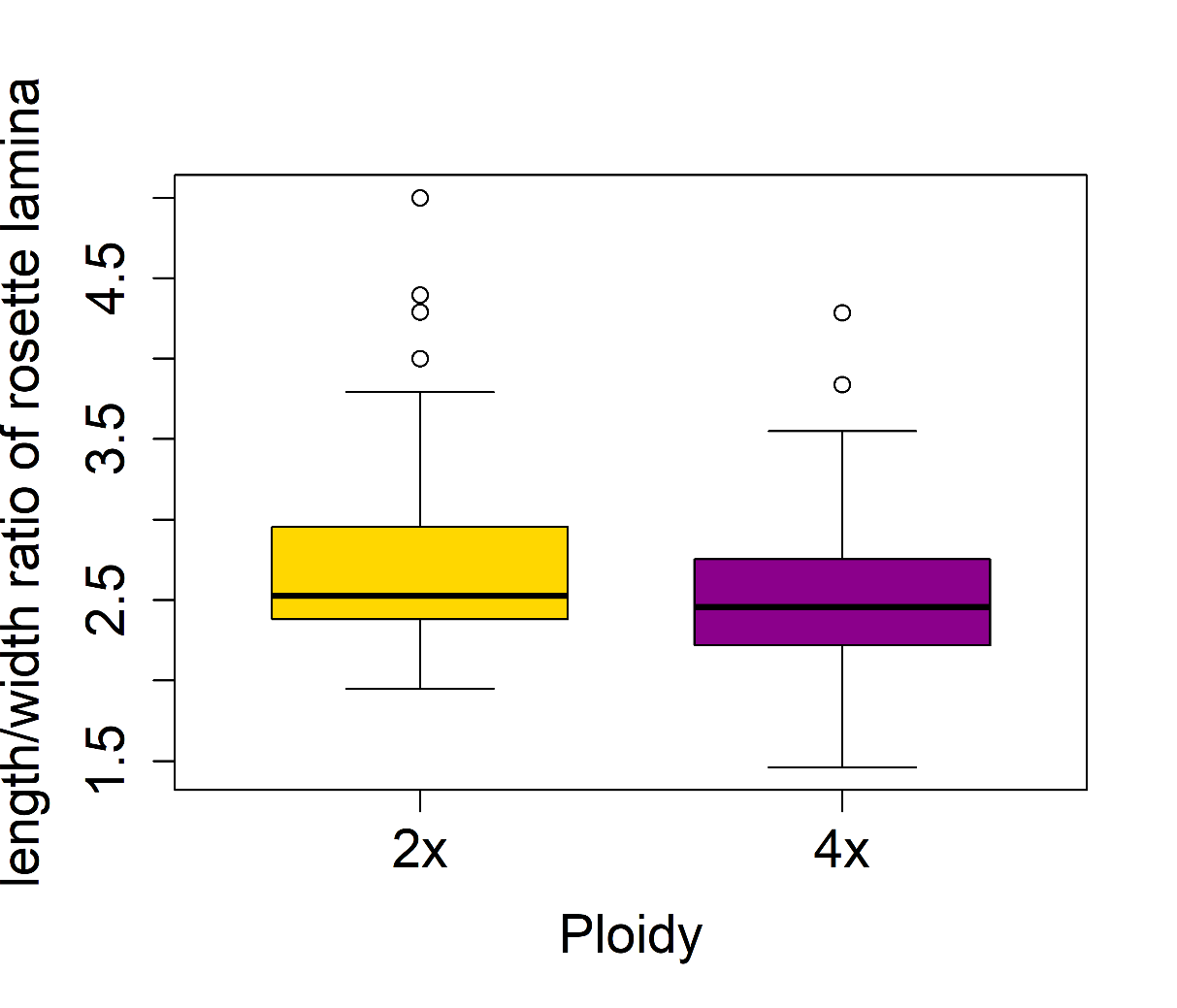

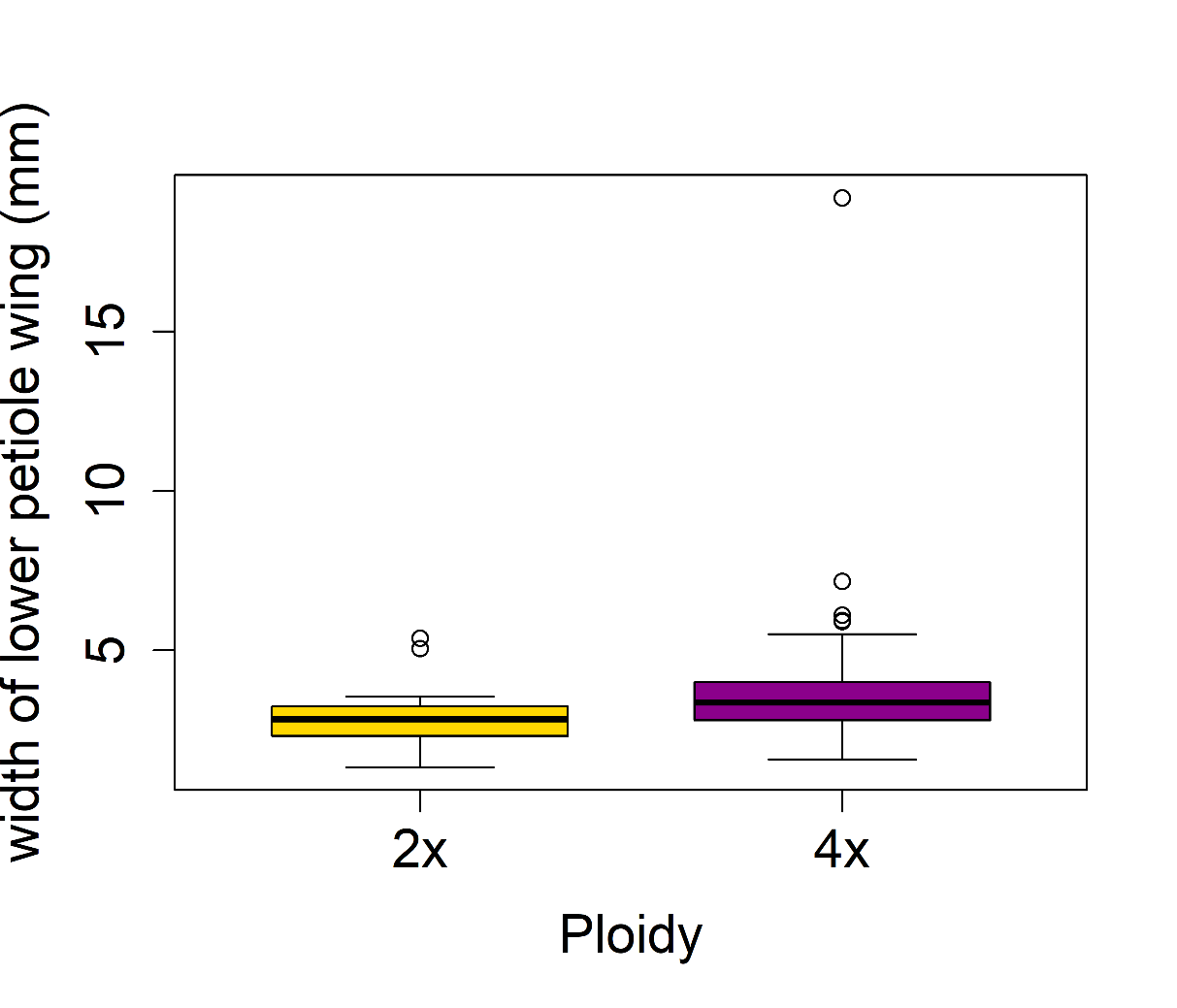

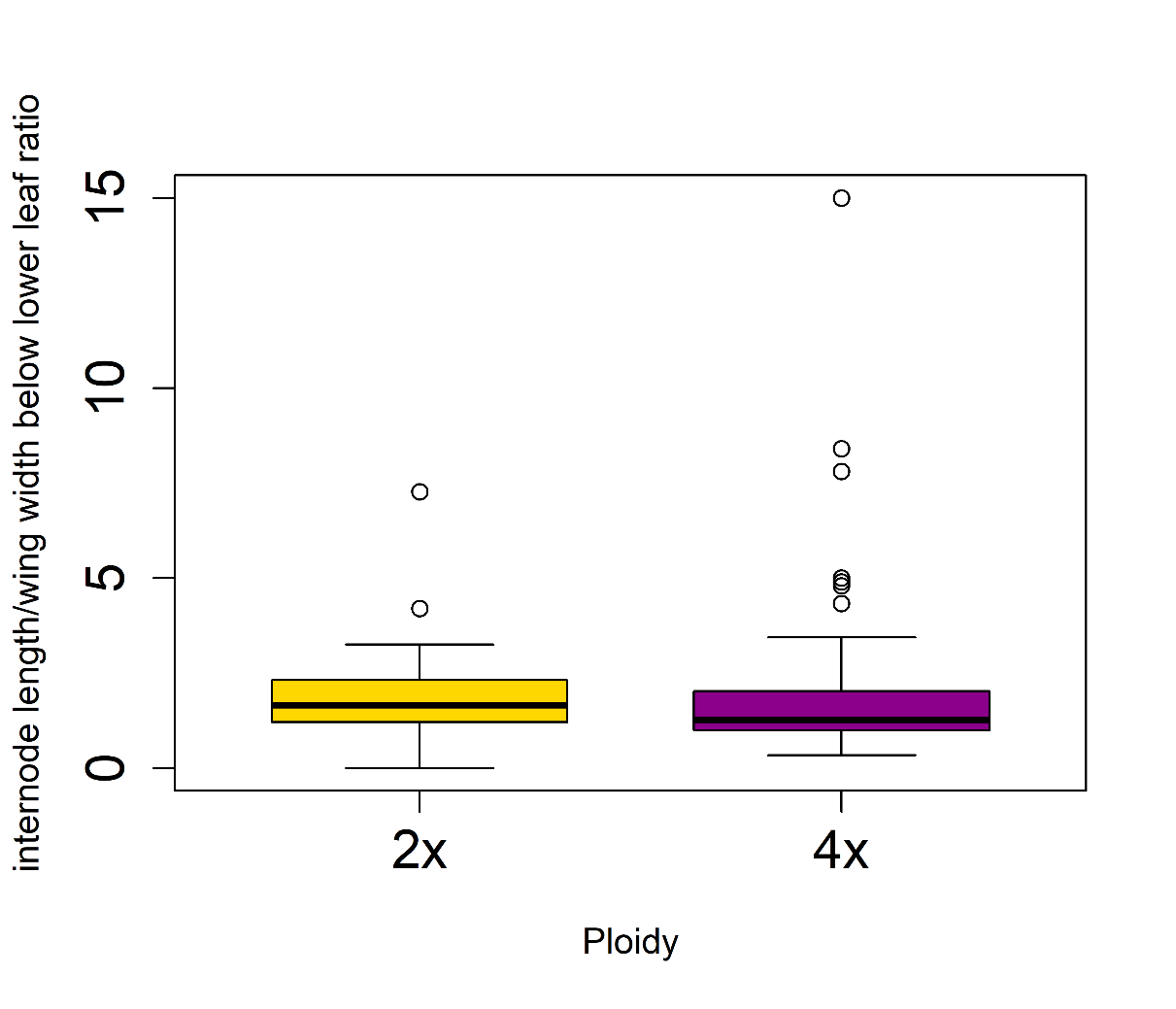

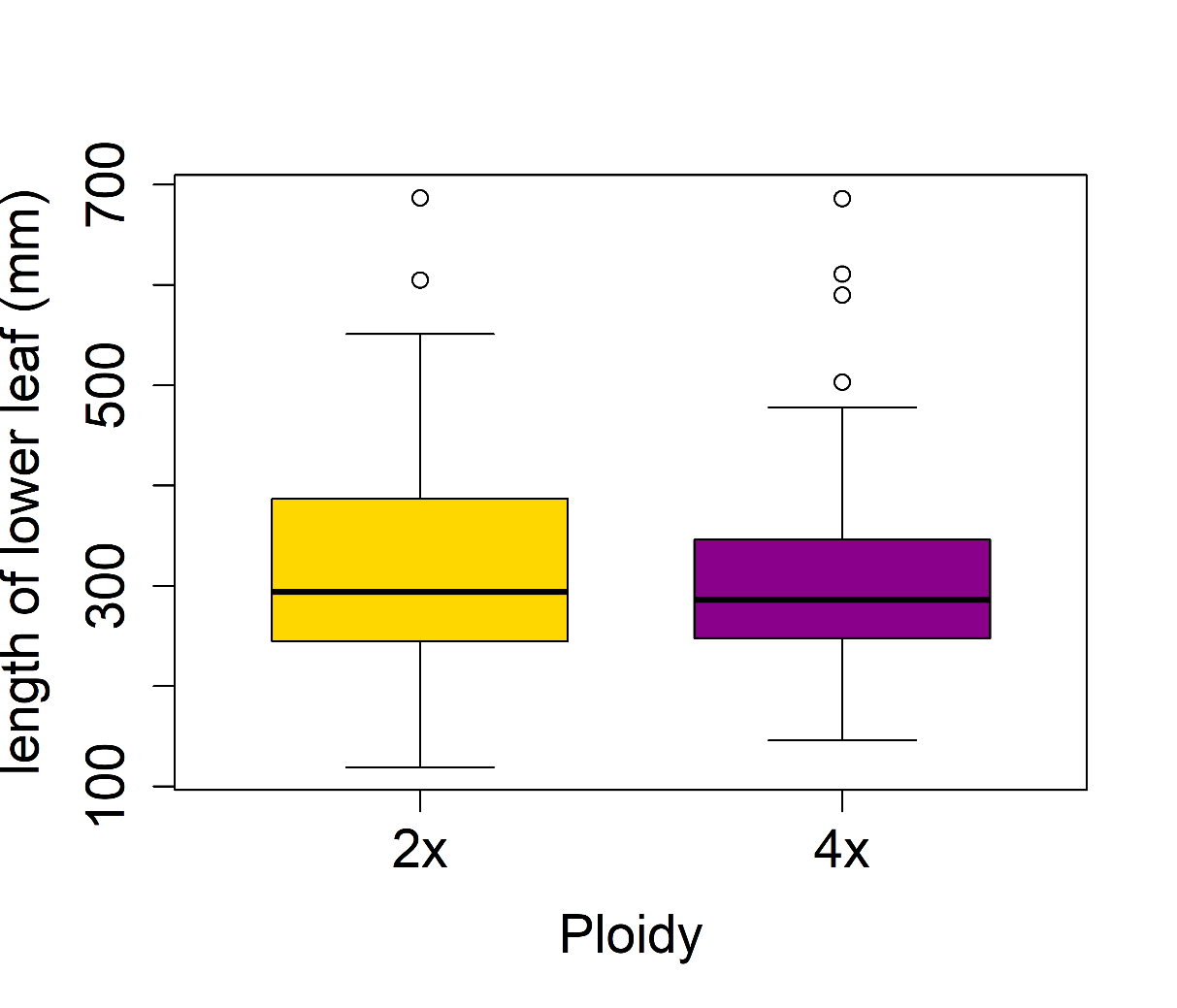

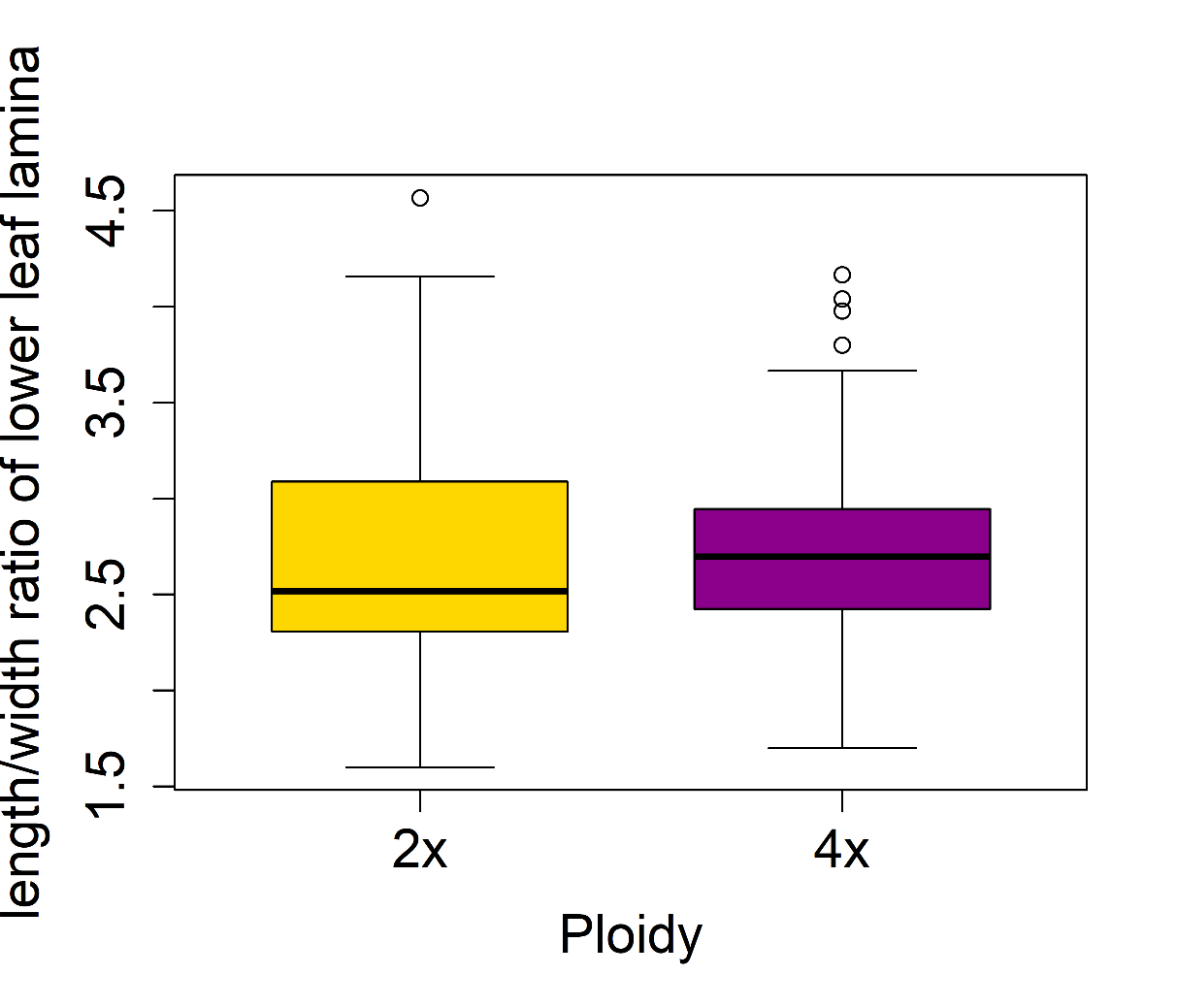

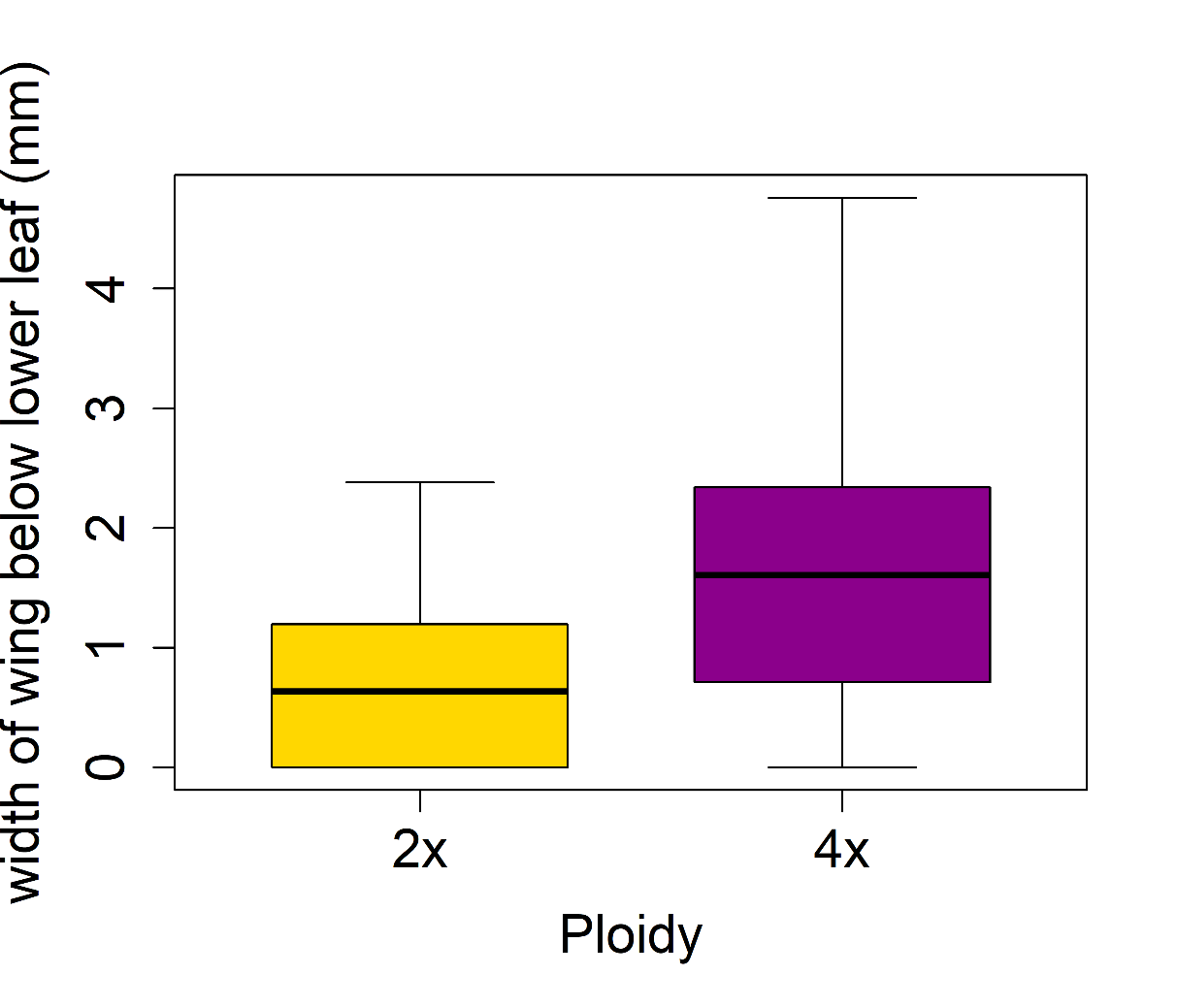

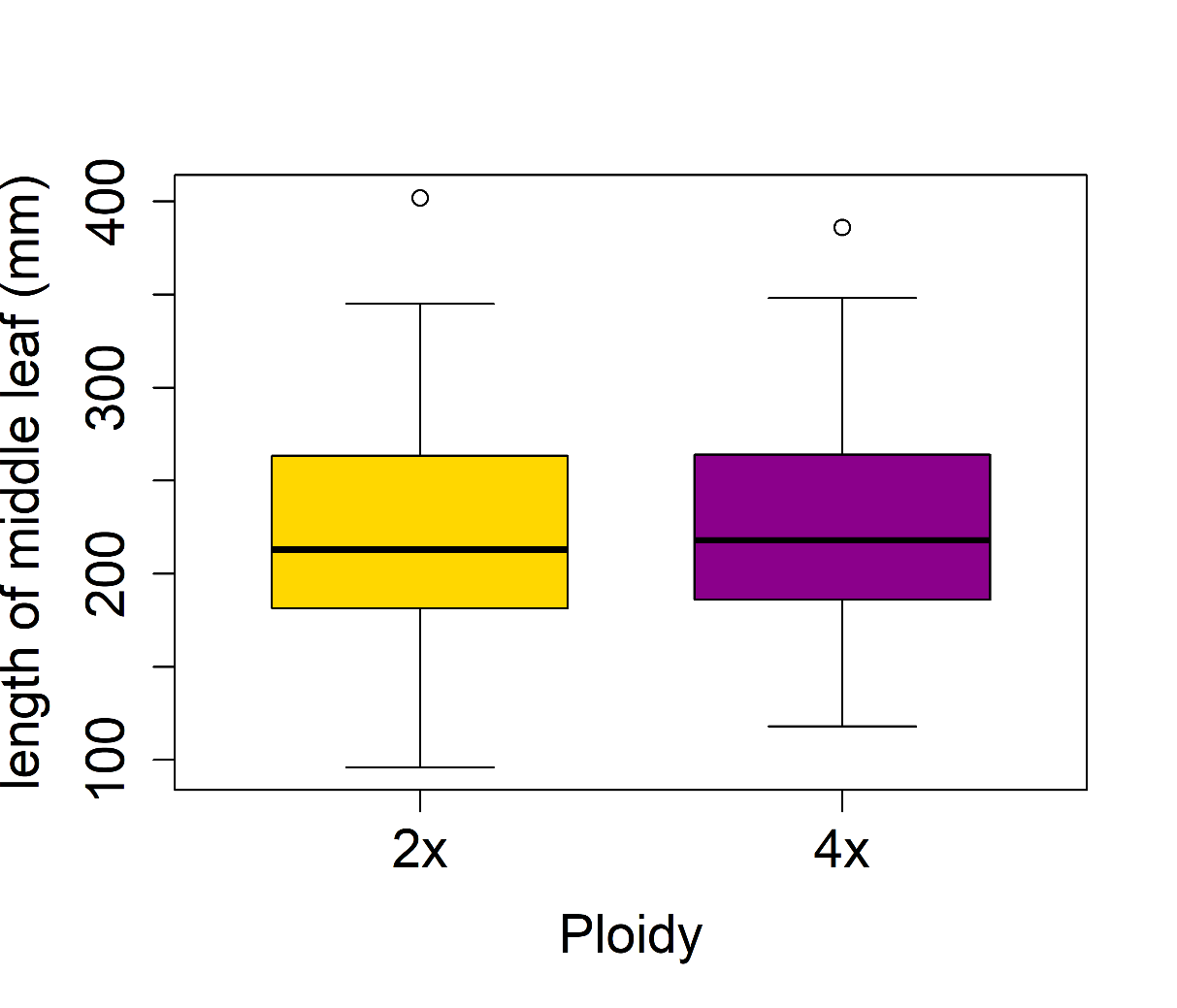

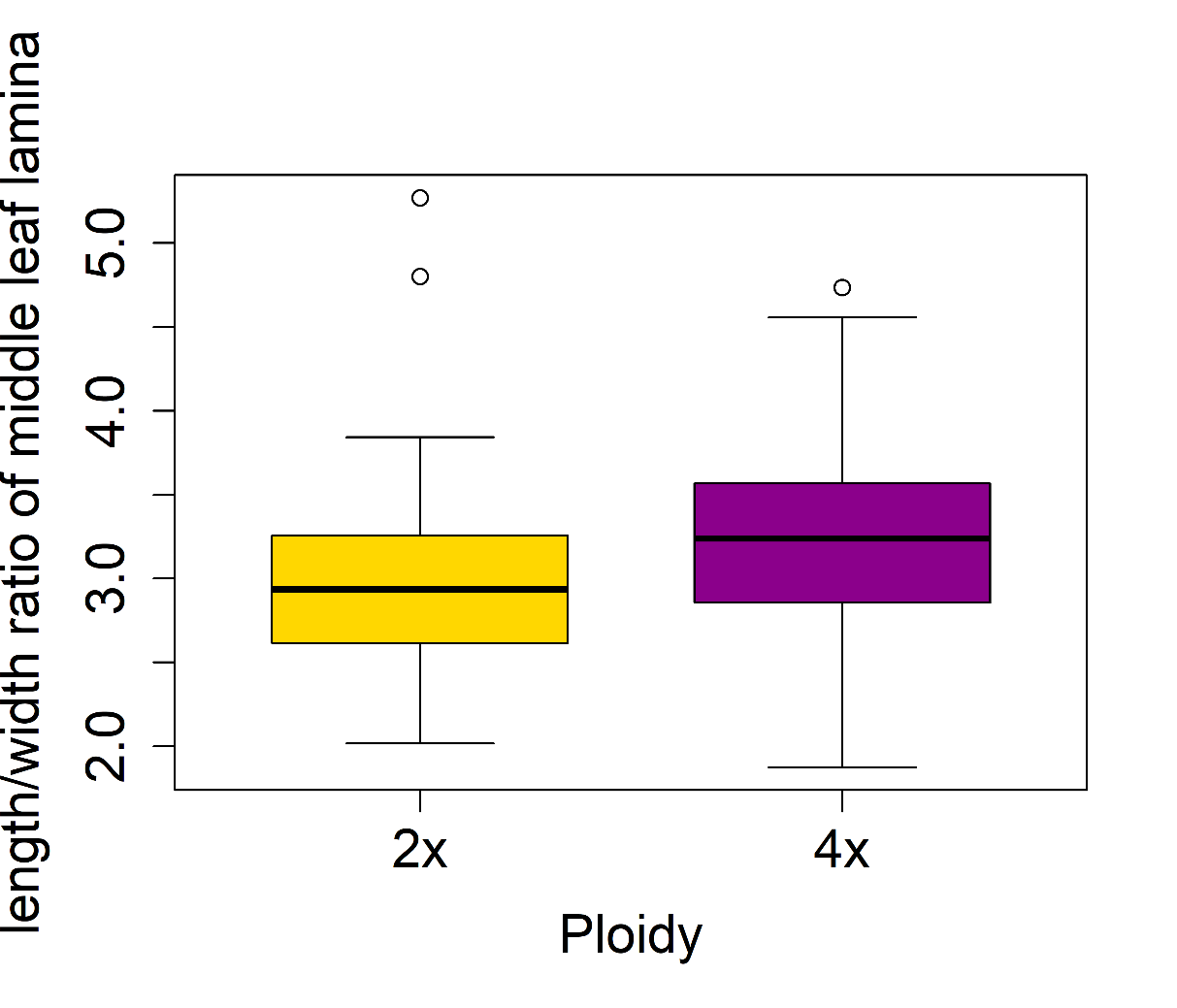

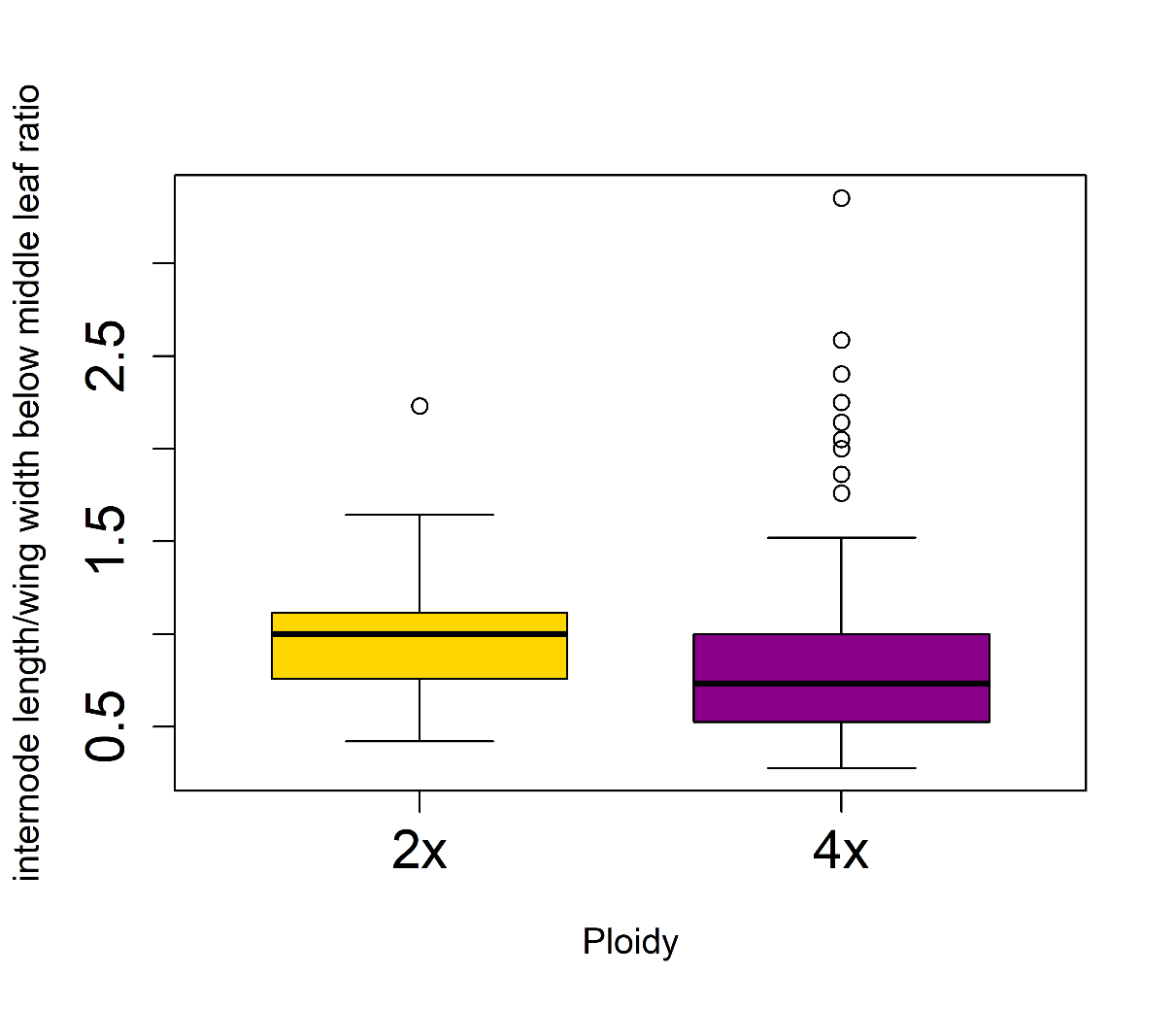

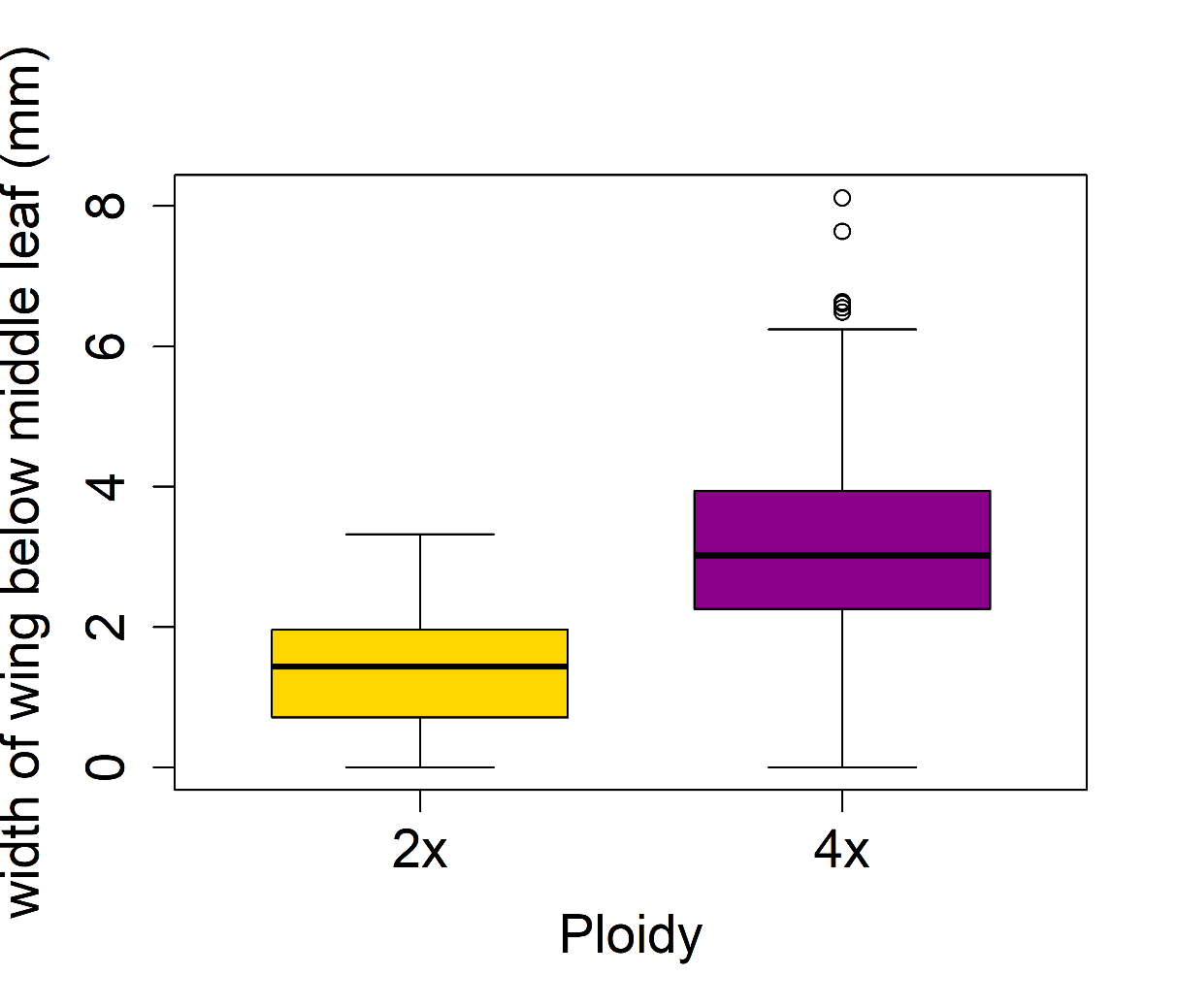

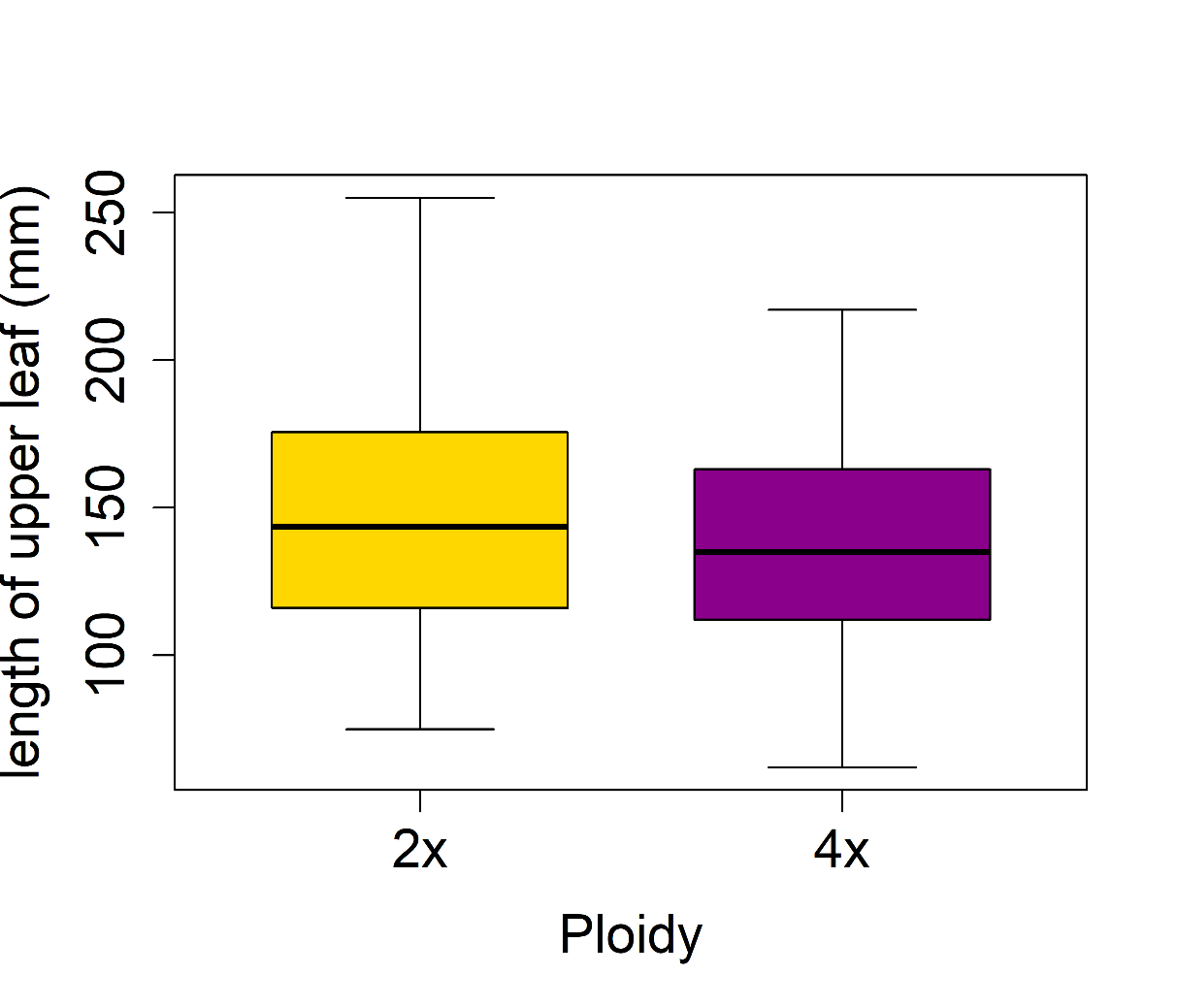

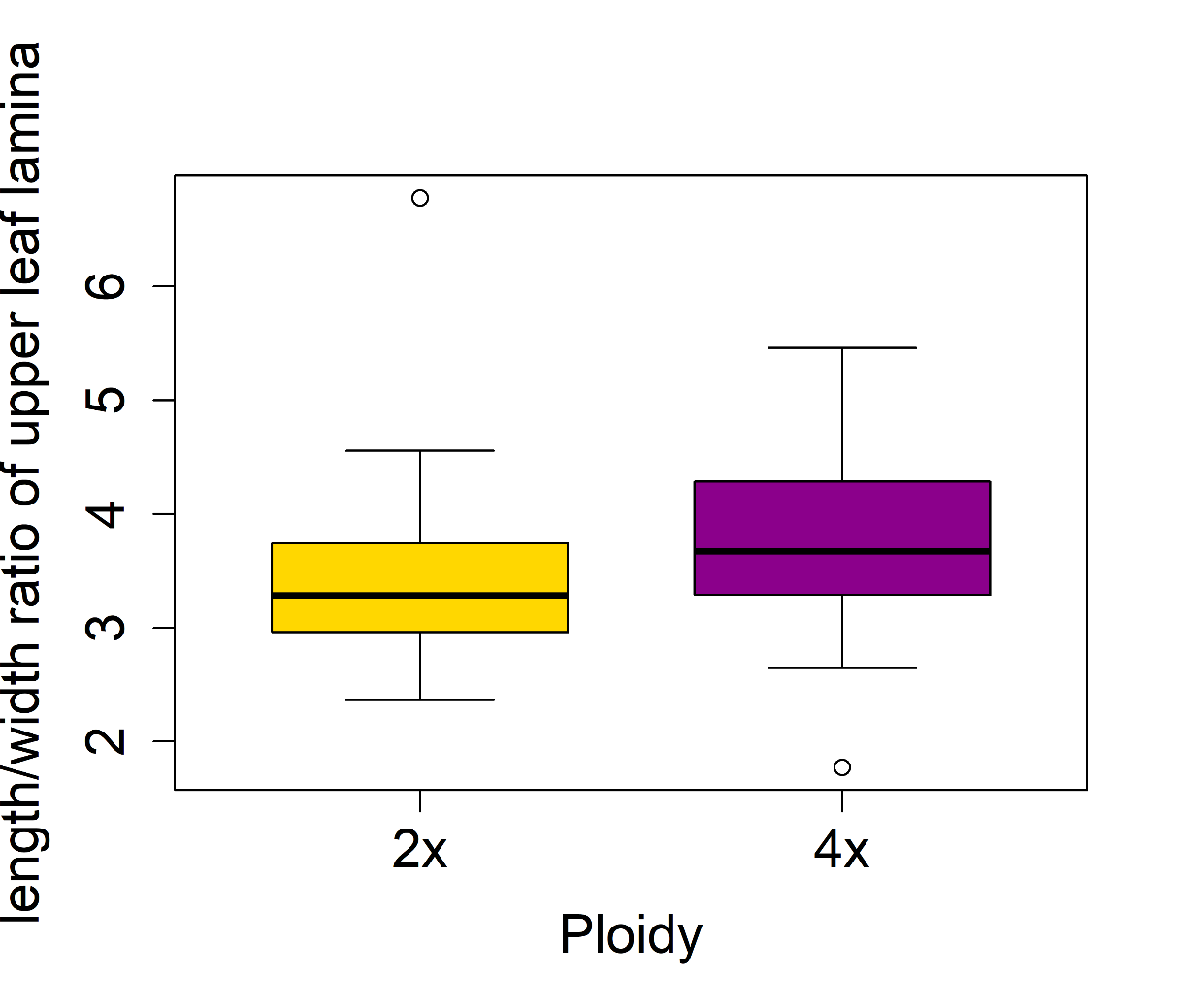

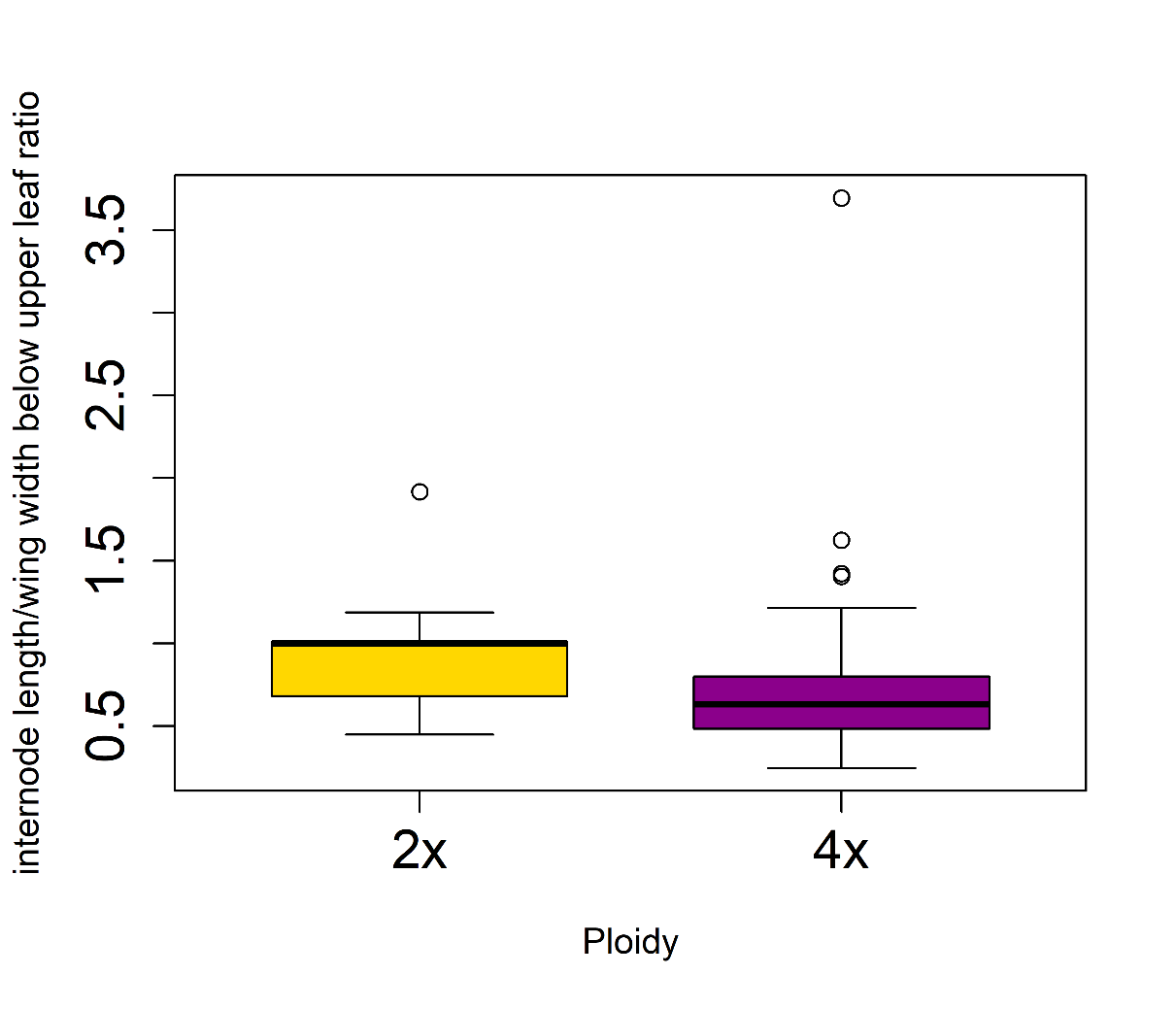

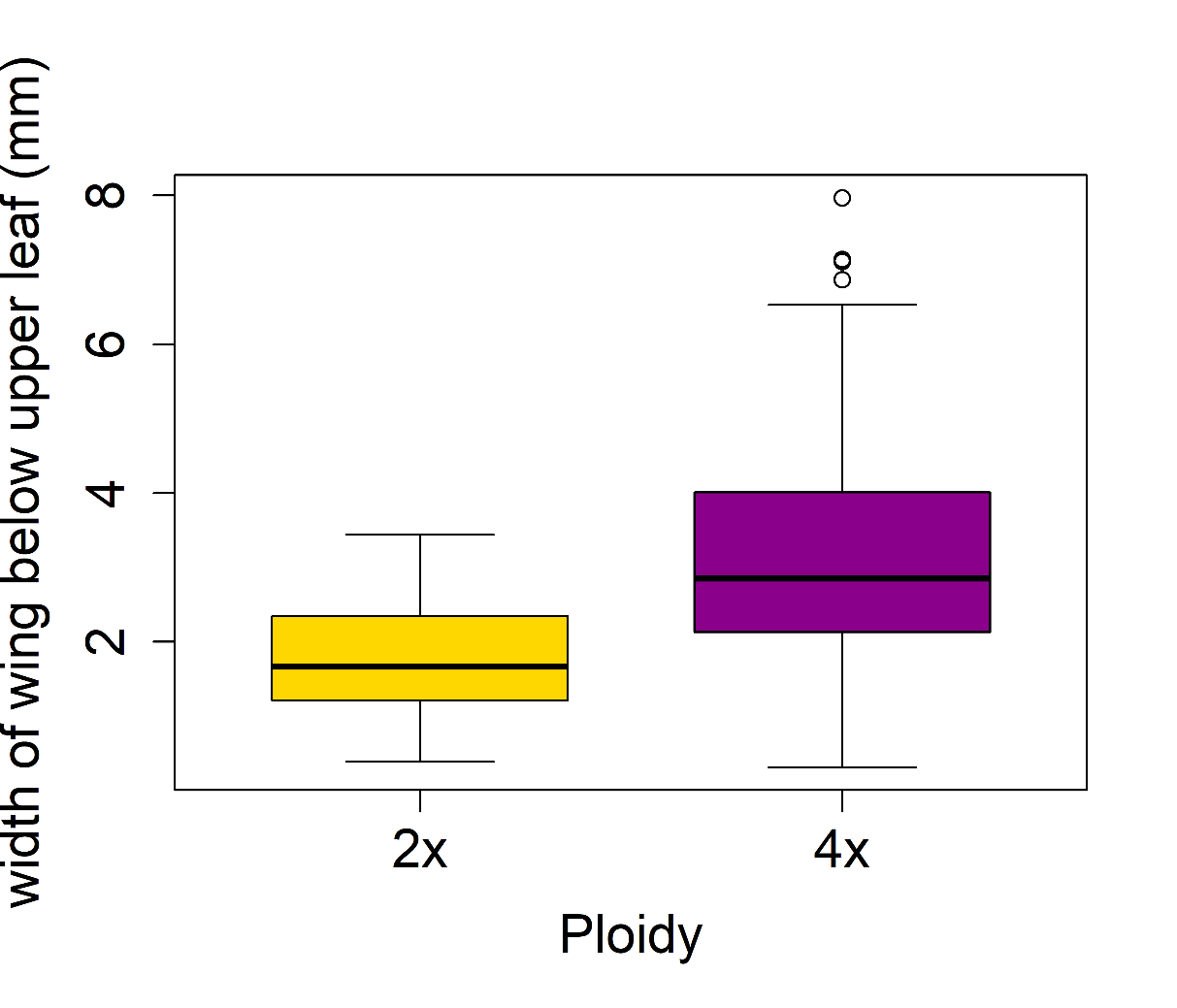

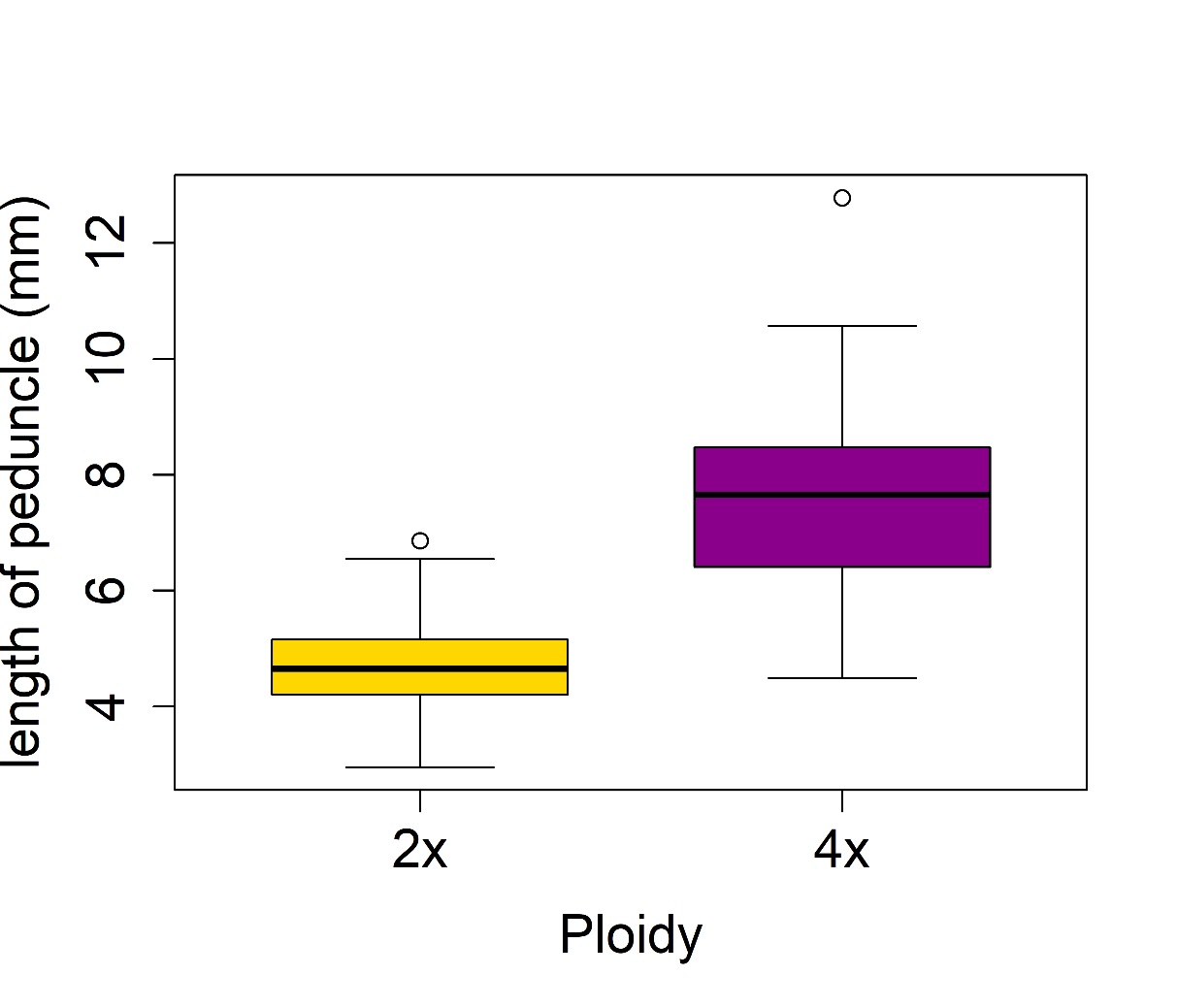

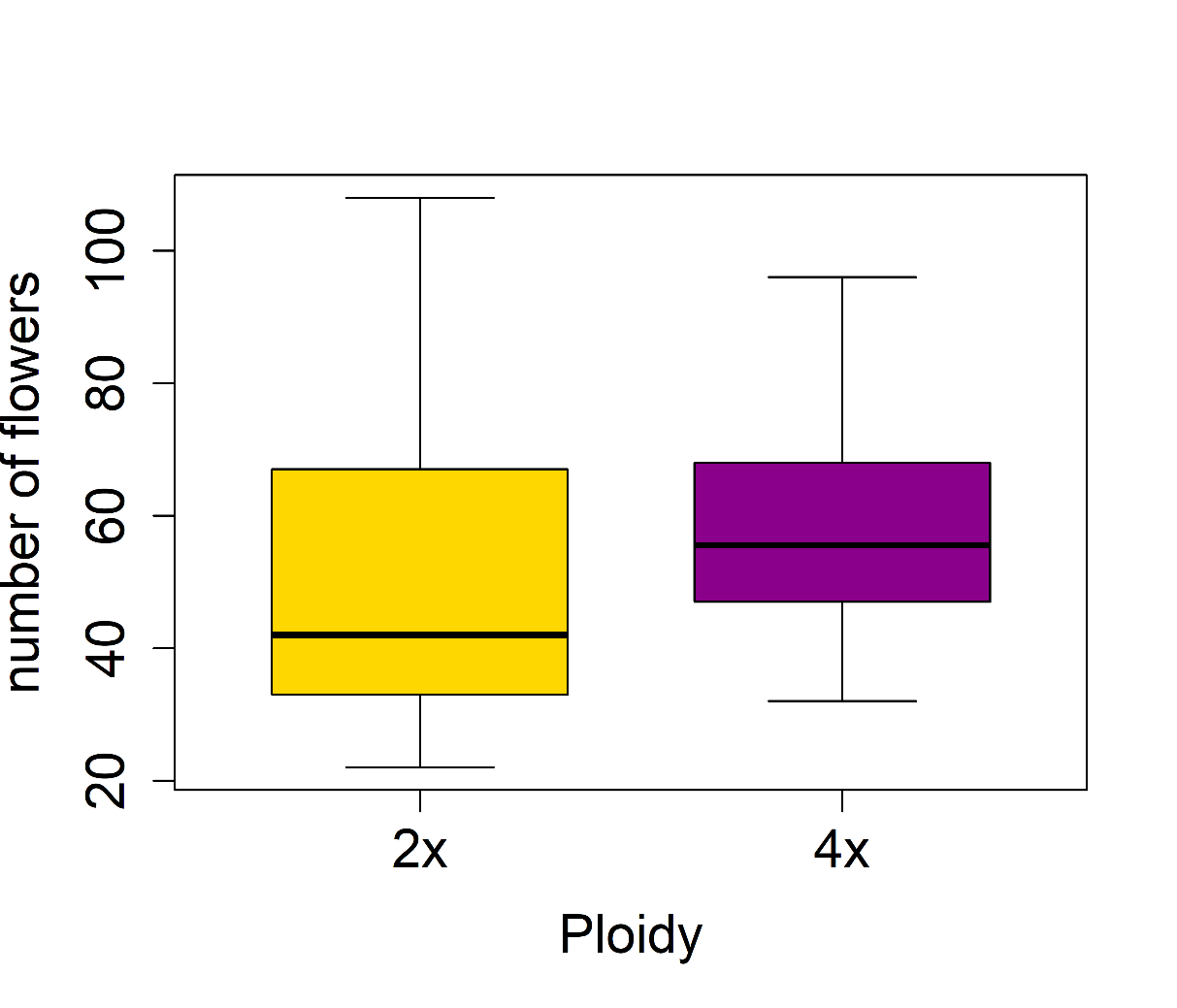

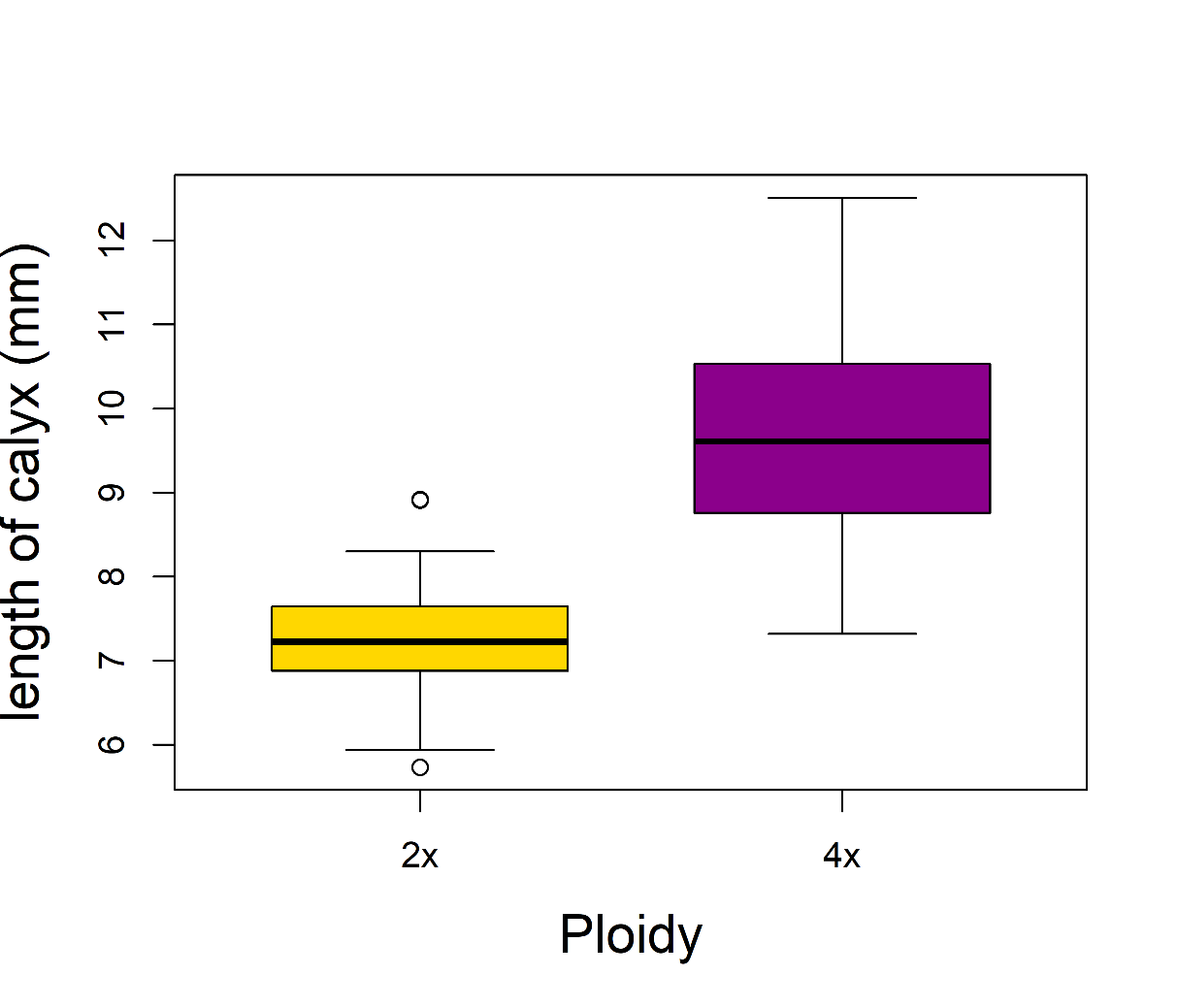

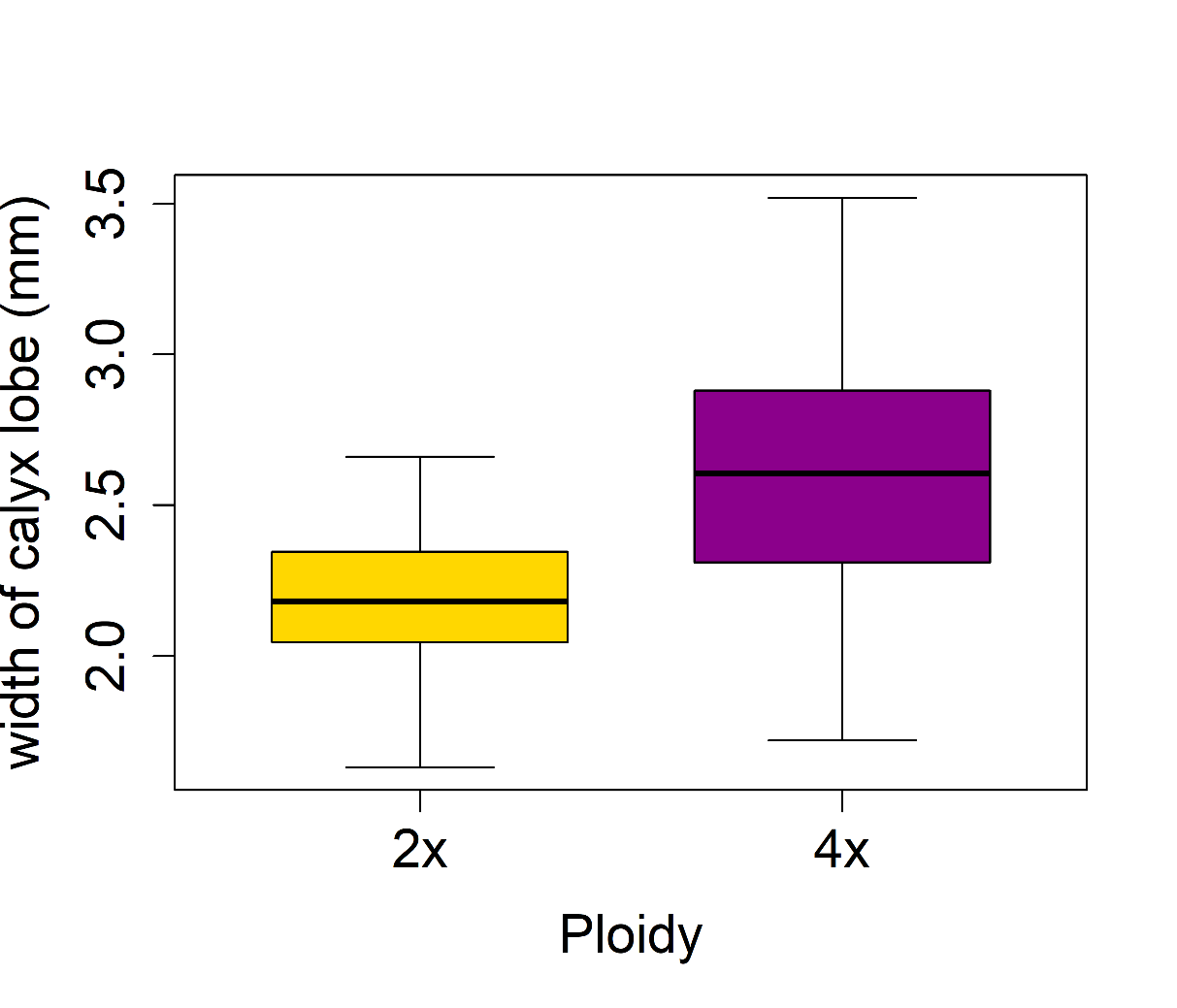

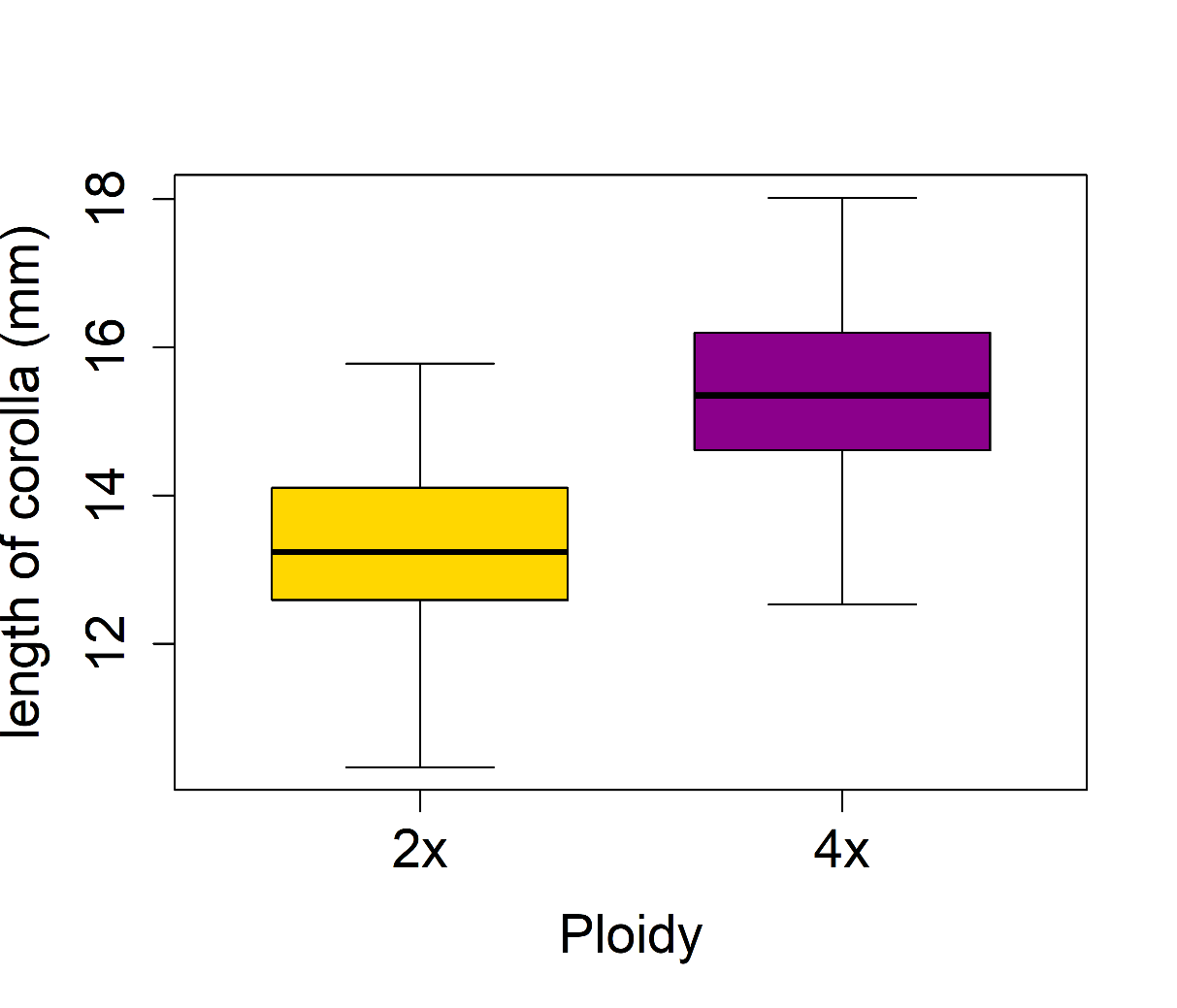

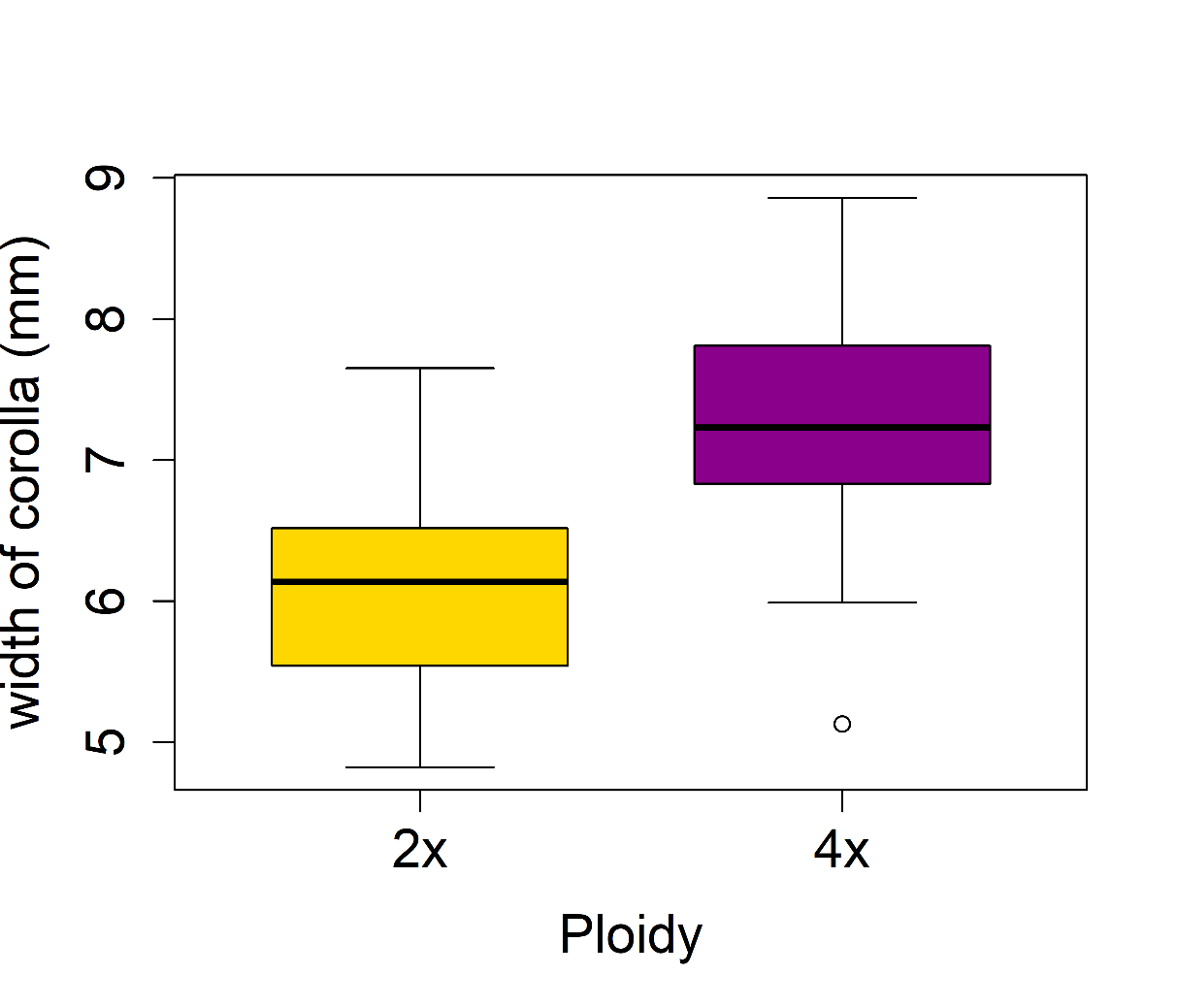

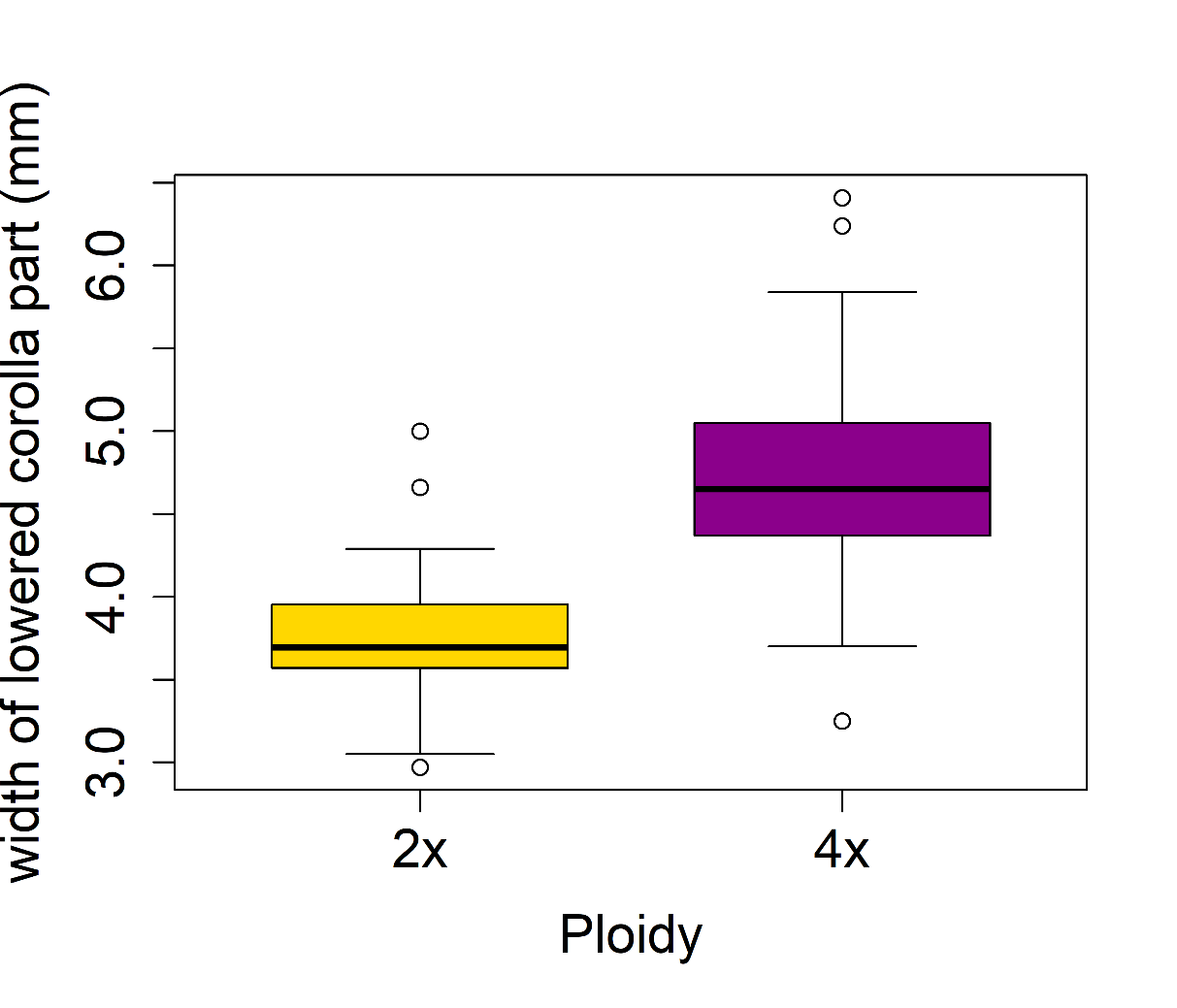

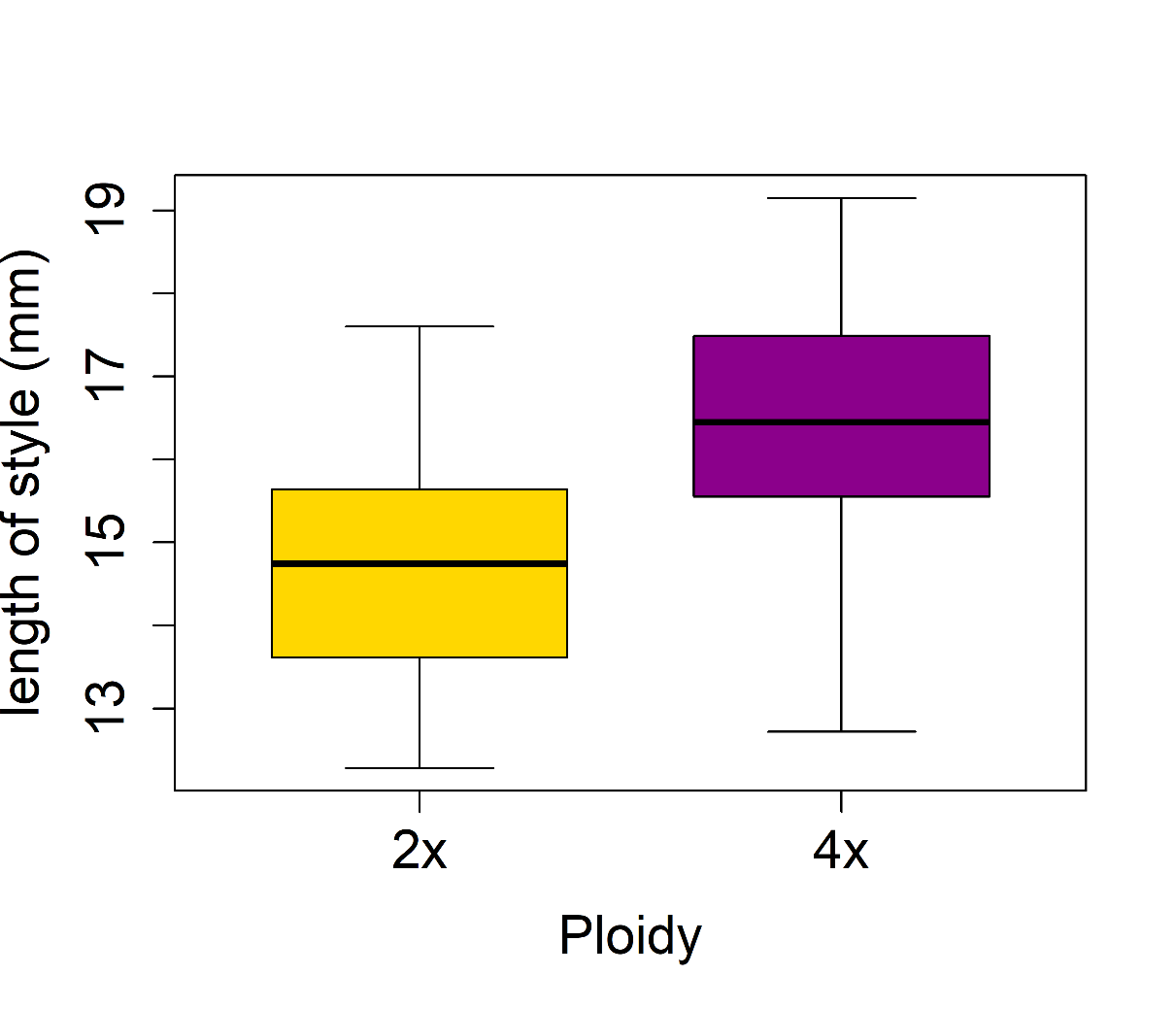

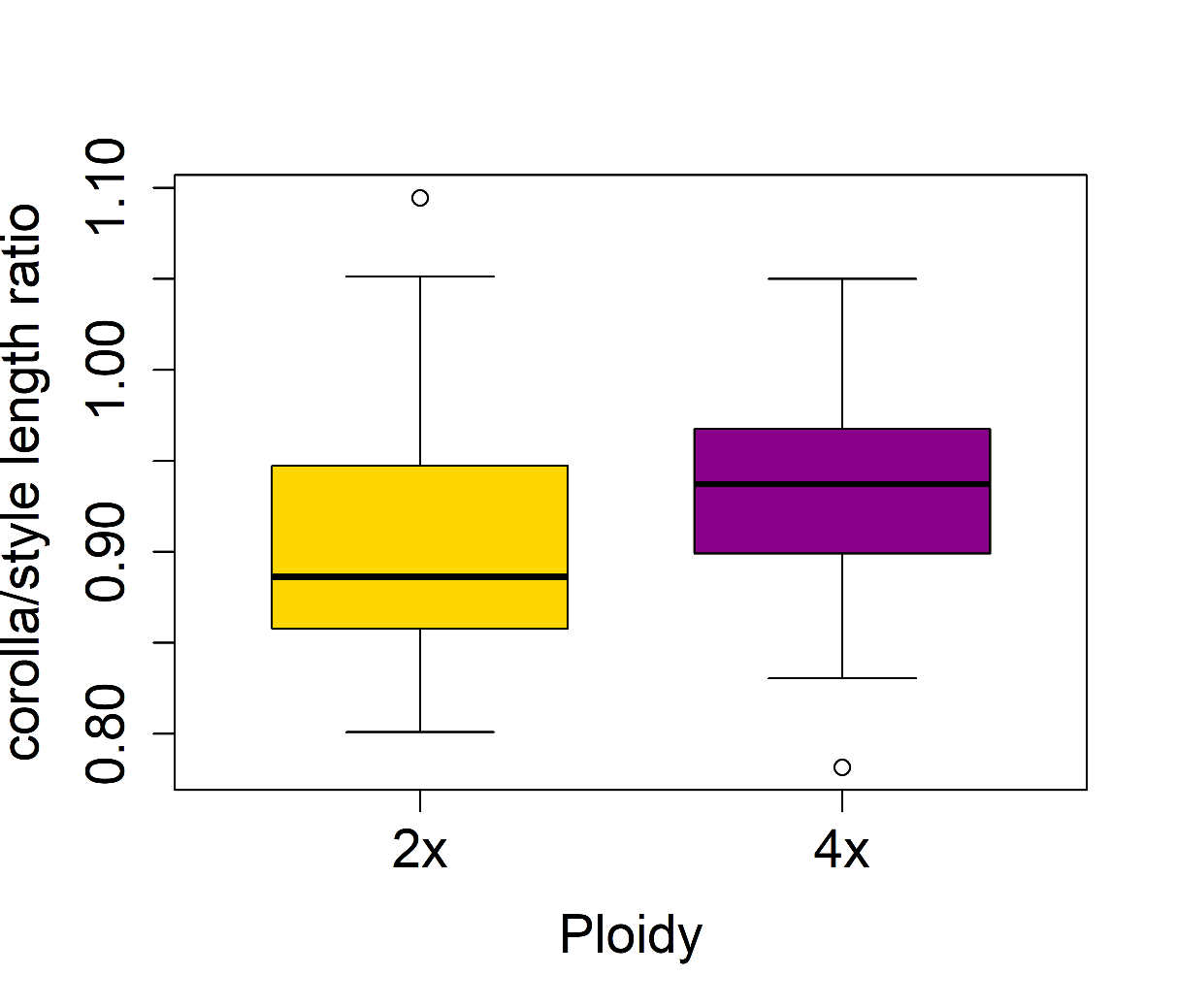

Supplement: plac028_suppl_Supplementary_Materials [file plac028_suppl_supplementary_materials.zip › plac028_suppl_Supplementary_Figures_LK.docx]
